# Mössbauer-based molecular-level decomposition of the *Saccharomyces cerevisiae* ironome, and preliminary characterization of isolated nuclei

**DOI:** 10.1093/mtomcs/mfac080

**Published:** 2022-10-10

**Authors:** Paul A Lindahl, Shaik Waseem Vali

**Affiliations:** Department of Chemistry, Texas A&M University, College Station, TX,USA; Department of Biochemistry and Biophysics, Texas A&M University, College Station TX,USA; Department of Chemistry, Texas A&M University, College Station, TX,USA

**Keywords:** cytosol, endoplasmic reticulum, iron–sulfur clusters, labile iron pool, mitochondria, nucleus isolation, vacuoles

## Abstract

One hundred proteins in *Saccharomyces cerevisiae* are known to contain iron. These proteins are found mainly in mitochondria, cytosol, nuclei, endoplasmic reticula, and vacuoles. Cells also contain non-proteinaceous low-molecular-mass labile iron pools (LFePs). How each molecular iron species interacts on the cellular or systems’ level is underdeveloped as doing so would require considering the entire iron content of the cell—the *ironome*. In this paper, Mössbauer (MB) spectroscopy was used to probe the ironome of yeast. MB spectra of whole cells and isolated organelles were predicted by summing the spectral contribution of each iron-containing species in the cell. Simulations required input from published proteomics and microscopy data, as well as from previous spectroscopic and redox characterization of individual iron-containing proteins. Composite simulations were compared to experimentally determined spectra. Simulated MB spectra of non-proteinaceous iron pools in the cell were assumed to account for major differences between simulated and experimental spectra of whole cells and isolated mitochondria and vacuoles. Nuclei were predicted to contain ∼30 μM iron, mostly in the form of [Fe_4_S_4_] clusters. This was experimentally confirmed by isolating nuclei from ^57^Fe-enriched cells and obtaining the first MB spectra of the organelle. This study provides the first semi-quantitative estimate of all concentrations of iron-containing proteins and non-proteinaceous species in yeast, as well as a novel approach to spectroscopically characterizing LFePs.

## Graphical Abstract

**Graphical Abstract:**
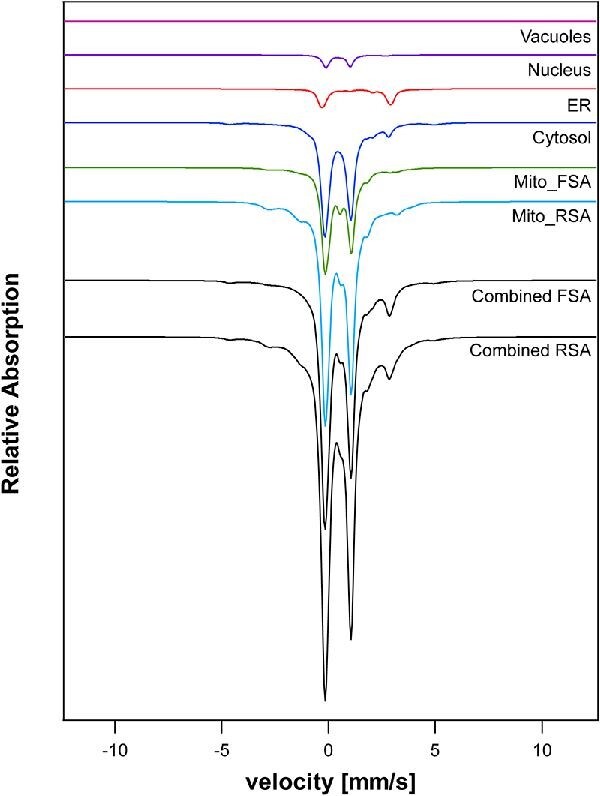
Lindahl and Vali consider all iron-containing proteins in a yeast cell in simulating the expected Mössbauer spectrum of whole yeast cells.

## Introduction

Iron is essential for all eukaryotic cells, from yeast to humans.^1,2^ This *d*-block transition metal possesses unique redox and substrate-binding properties that make it essential for catalyzing difficult cellular reactions. Iron is found in proteins as hemes, iron–sulfur clusters (ISCs), diiron-oxo centers, and mononuclear iron centers.^3^ Mitochondria contain respiration-related proteins rich in hemes and ISCs.^4^ Iron-containing enzymes in the cytosol catalyze myriad reactions, often involving primary metabolism. Endoplasmic reticula contain iron enzymes involved in the biosynthesis of membrane components, including fatty acids and ergosterol, a cholesterol analogue. The nucleus contains many DNA replication and repair enzymes that house [Fe_4_S_4_] clusters. Under iron-replete conditions, vacuoles sequester and store iron as Fe^III^ polyphosphate complexes.^5^ The cytosol also contains a non-proteinaceous labile iron pool (LFeP) that is involved in trafficking, signaling, and/or regulation.^6^

Ironically, the same properties that render iron essential to cells also make it dangerous for them. Iron can participate in Fenton chemistry to generate reactive oxygen species (ROS) that can damage cellular macromolecules including DNA, proteins, and membranes.^7^ Thus, nutrient iron entering cells must be carefully trafficked to various locations for installation into client apo-proteins.

Although many molecular-level players involved in iron metabolism have been identified and characterized, a systems’ level understanding of iron trafficking and homeostatic regulation is lacking since such a characterization would require consideration of the entire iron content of the cell—the *ironome*. Here we offer a novel albeit imperfect approach to study the ironome of a yeast cell, involving Mössbauer (MB) spectroscopy.

MB is the most powerful spectroscopic tool to study iron, as it can distinguish between different types of iron centers, different iron oxidation states, and different magnetic spin states.^8^ The MB-active stable isotope ^57^Fe possesses nuclear spin I = ½; all other iron isotopes, which collectively represent ∼98% of natural-abundance iron, are MB-silent. The MB spectral intensity associated with each ^57^Fe atom in a sample is approximately the same, such that the percentage of a particular spectral feature can be approximately equated to the percentage of the corresponding iron-containing molecular species in the sample. For these reasons, MB can be used to decompose and quantify the cell's ironome. Decomposing the ironome into individual iron centers is not possible using MB spectroscopy due to its limited resolution. Rather, MB can only generate a supramolecular or coarse-grain analysis of a cell's iron content. Nevertheless, no other spectroscopic technique can do better.

The objective of this study was to simulate the MB spectral patterns observed for whole cells and isolated organelles by summing the spectral contribution of each iron-containing species in the cell. First, we updated a published list of iron-containing proteins in *Saccharomyces cerevisiae* and organized the resulting proteins into five cellular compartments, including cytosol (cyt), mitochondria (mit), vacuoles (vac), nuclei (nuc), and endoplasmic reticula (er).^3^ Published electron microscopy results were used to estimate the volume of a cell and the fractional volume of each cellular compartment. Yeast proteomic data were used to estimate the cellular concentration of each iron protein.^9^ Existing spectroscopic data were used to assign the type of iron center to each iron protein. Thermodynamic reduction potentials for each center were then used to estimate the oxidation state of each iron center in each iron-containing protein. MB spectra for each iron center in their expected oxidation state were then simulated (using WMOSS software; www.wmoss.org) and summed, with relative intensities weighted according to the cellular concentration of each contributing protein. Composite simulations were compared to experimentally determined MB spectra of whole cells, as well as isolated mitochondria and vacuoles. Simulated MB spectra of non-proteinaceous iron species in the cell were added to account for differences between experimental spectra and simulated spectra of the iron-containing proteome. The iron content of isolated mitochondria and vacuoles have been analysed by MB spectroscopy, and these were used in the analysis.^5,10,11^ The first MB spectra of isolated nuclei were obtained. Experimental spectra were compared to those predicted by simulations, affording new insights into the ironome of yeast cells.

## Experimental procedures

### Isolation of nuclei

Nuclei were harvested from *S. cerevisiae* strain W303 (*MATa*/*MATa* {*leu2-2, 112 trp1-1 can1-100 ura3-1 ade2-1 his3-11, 15*} [*phi+*]). Single colonies were inoculated into 50 mL of complete synthetic media (CSM-Ura + 20mM Uracil, Sunrise Science Products). Cell growth was monitored by measuring optical density (OD). Once the cells reached an OD = 1.0 ± 0.1, they were transferred into a 2.5 L baffled flask containing 1 L of the same media. Cells in 50 mL and 1 L cultures were grown at 30°C in a shaker incubator (Amerex Instruments) at 130 rpm. At OD = 1.0 ± 0.1 cells were transferred into a glass/titanium custom-built bioreactor containing 24 L of the same media at 30°C. Air was bubbled through the medium at ∼1 L/min. Cells were harvested between OD 1.2 and 1.4.

Nuclei were isolated following a recent method (https://www.biorxiv.org/content/10.1101/162388v1) with some modifications. Cell pellets were combined into 1 pre-weighed centrifuge bottle. The bottle containing the weighed cell pellet was brought into an anaerobic glovebox (Mbraun Labmaster), washed with water, and resuspended in ∼500 mL of degassed 100 mM Tris buffer pH 7.4. DTT was added (10 mM final) and the suspension was placed in a shaker incubator at 30°C for 10 min at 100 rpm. Cells were spun at 5000 *×g* (Sorvall Evolution) for 5 min and returned to the box. The pelleted cells were washed twice with ∼250 mL ice-cold distilled degassed water. Cells were then resuspended in ice-cold SA buffer (100 mL per 20 g of cells) (SA buffer = 1.2 M sorbitol, 20 mM ammonium bicarbonate, 1 mM EDTA, 60 mM NaCl, pH 7.4) and centrifuged at 5000 ×*g* for 5 min. The resulting pellet was resuspended in SA buffer with 5 mM DTT (100 mL per 40 g of cells). A whole-cell aliquot was removed for western blot. Cells were digested using 3 mg zymolyase (AMBIO) and 5 mg lyticase (Sigma-Aldrich) per gram of cell pellet. A 1 mL aliquot was removed in an Eppendorf tube to monitor cell-wall digestion. The cell suspension was placed in a shaker incubator at 30°C for ∼1 hr at 130 rpm. Spheroplast formation was assessed by diluting 10 μL of the suspension from the aliquoted Eppendorf tube with 990 μL of DI water and measuring the OD of the mixture. After the OD reached ∼25% of its initial value, the suspension was pelleted and the pellet was resuspended in ∼200 mL of lysis solution. Lysis solution was obtained by mixing 245 mL of 8% polyvinylpyrrolidone, 12 mM KH_2_PO_4_, 8 mM K_2_HPO_4_, 7.5 mM MgCl_2_, pH 6.5 with 2 mL of solution P (90 mg of PMSF dissolved in 5 mL of EtOH), 625 μL (10% Triton X-100), and 5 mM DTT. The resulting mixture was homogenized inside the box, 40 mL at a time, using 25 strokes from a Dounce homogenizer (Type B pestle). The resulting homogenate was transferred into a 250 mL centrifuge bottle and centrifuged at 1500 *×g* for 5 min. The supernatant was collected and spun again for a total of four to five spins, each time discarding the pellet. An aliquot of this resulting cell lysate was used for western blot. Ten to twelve density gradients were prepared using 2.5 M sucrose and 1.80 M sucrose in 20 mM Bis-Tris, 20 mg MgCl_2_.6H_2_O, pH 6.5. Eight milliliters of the 2.5 M sucrose solution was placed at the bottom of a 50 mL plastic insert for a Sw32 Ti rotor, carefully overlaid with 8–10 mL of the 1.80 M sucrose solution followed by the lysate on top. The tubes were sealed in canisters, removed from the box, and spun six gradients at a time, at 103 000 *×g* for 30 min at 4°C in a Beckman Coulter Optima 90K ultracentrifuge. Canisters were returned to the box, and nuclei were collected at the interface of the sucrose layers using a pipette. Nuclei fractions were kept in a 2 M sucrose solution to maintain osmotic pressure. MB samples were prepared by pelleting the nuclei into a MB cup at 10 000 *×g* for 10 min followed by freezing in liquid N_2_. The electron paramagnetic resonance (EPR) sample was prepared by pelleting the nuclei fraction into an EPR tube (4 mm Thin-wall Precision Suprasil, Wilmad-LabGlass) using a custom SLC-1500 rotor in a Sorvall Centrifuge at 4000 *×g* for 30 min followed by slowly freezing in LN_2_.

### Liquid chromatography-inductively coupled plasma-mass spectrometry (LC-ICP-MS) analysis analysis

MB samples of isolated nuclei were thawed inside the glovebox and washed with ice-cold degassed DDDI water (double distilled and deionized using a sub-boiling distillation apparatus). The suspension was transferred to an epi-tube, and centrifuged at 15 000 ×*g* (Sorvall Legend micro21R) for 10 min outside the box. The sample was returned into the box and the volume of the pellet was determined. For nuclei, 70% of the packed volume of the pellet was assumed to correspond to the volume of the nuclei. Pellets were resuspended in 2 mL of ammonium acetate buffer (20 mM ammonium acetate pH 6.5) and 2% Triton X-100 along with a few grains of DNase (Thermofisher) powder. The solution was vortexed for 2 min followed by 5 min rest. The vortexing cycle was carried out 4*×* followed by centrifugation (sealed, outside the box) at 12 000 *×g* for 15 min. The resulting supernatant was returned to the box, loaded into a filtration apparatus (10 kDa cutoff membrane Amicon Ultra Filters 2mL; Millipore) and spun by centrifugation (again sealed, outside the box). Flow-through solution (FTS) from the filtration step was analysed in the box using the LC–ICP–MS (Agilent 7700x) in He collision mode as described.^11^

### Metal analysis

Metal concentrations of isolated nuclei were obtained by ICP–MS. After collecting MB/EPR data, some samples were thawed and packed into EPR tubes to measure sample volumes (150–300 μL) and then diluted with 250 μL of DDDI water. Other samples were used directly after isolation. 100 μL aliquots of resulting suspensions were transferred into 15 mL Falcon tubes and 250 μL of concentrated trace-metal grade HNO_3_ was added. Each tube was sealed and heated overnight (14–16 hr) at 80°C. Digested samples were diluted to a final HNO_3_ concentration of 5% and analysed using ICP–MS. Concentrations were calibrated using primary P, S, Mn, ^56^Fe, ^57^Fe, Cu, and Zn standards (Inorganic Ventures, 5000 μg of metal/L each). Secondary standards (0, 10, 50, and 100 μg/L for each metal and 0, 1000, 5000, and 10 000 μg/L for P and S) were used for calibration.

### Microscopy and western blots

A 2-well Lab-Tek Chambered Borosilicate coverglass (Fisher Scientific) was coated with 20 mL of 0.1% (w/v) poly-L-Lysine solution (Sigma-Aldrich) for 30 min and dried. A yeast strain (Nup49p-GFP; Yeast GFP Clone Collection; Thermofisher) in which GFP was tagged to the nucleolar protein Nup49 was used. Stock solutions of Dil Stain (1,1′-Dioctadecyl-3,3,3′,3′-Tetramethylindocarbocyanine Perchlorate; Invitrogen), 250 μg/mL, and DAPI (200 μM) (Invitrogen) were prepared in dimethyl sulfoxide and phosphate-buffered saline, respectively. Freshly isolated nuclei (20 μL) were mixed with 2 μL of Dil Stain and 2 μL of DAPI followed by incubation for 10 min at RT. Fluorescent stained nuclei were then applied to the coated cover glass and imaged with a Zeiss LSM 780 confocal microscope. Western blotting was performed using nuclei from W303 cells as described.^12^ Anti-nop1p antibody (Santa Cruz Biotechnologies) was used as a nuclear marker at 1:500 dilution.

## Analysis and results

### Description of iron-containing proteins in yeast

We have identified 100 iron-containing proteins in *S. cerevisiae* (Supplementary Table S1), including 37 in mitochondria, 34 in the cytosol, 15 in the ER, 13 in the nucleus, and 1 in vacuoles. The cytosol imports iron from the environment, and sends much of it to the mentioned organelles. Some iron is installed into iron-containing cytosolic proteins, and some flows into mitochondria and perhaps the ER. Some mitochondrial iron might flow back into the cytosol, possibly as glutathione-bound [Fe_2_S_2_] clusters.^13^ Some such clusters are assembled into [Fe_4_S_4_] clusters by cytosolic ISC assembly (CIA) proteins, and these are installed into target apo-proteins mainly in the cytosol and nucleus.^14^ Other mitochondria-exported [Fe_2_S_2_] clusters might help regulate the “iron regulon” genes that are involved in iron homeostasis. **Aft1** and **Aft2** are paralogous transcription factors that, in their apo-forms, locate in the nucleus where they bind DNA and promote expression of the iron regulon.^15,16^ (Each iron-containing protein is highlighted in bold when first introduced. Associated references are not comprehensive but were selected to introduce unfamiliar readers to each protein. Some references may refer to homologs from other organisms.) Under iron-replete conditions, these proteins accept a [Fe_2_S_2_] cluster to form Aft1[Fe_2_S_2_]Aft1 and Aft2[Fe_2_S_2_]Aft2 dimers, with the cluster bridging the two subunits. They receive their cluster from **Grx3**[Fe_2_S_2_]**Bol2** and **Grx4**[Fe_2_S_2_]Bol2 heterodimers comprised of the Bol2 protein and either monothiol glutaredoxin Grx3 and Grx4, again with the cluster bridging.^17–20^ The cluster in Aft1/2[Fe_2_S_2_]Aft1/2 is coordinated by four cysteine exoligands, while that in Grx3/4[Fe_2_S_2_]Bol2 are coordinated by two cysteines from Grx3/4, one histidine from Bol2, and an S donor perhaps GSH.^21^**Apd1** is a cytosolic protein of unknown function that contains an [Fe_2_S_2_] cluster with bis–his bis–cys coordination.^22^

**Dre2** provides the electrons required by the CIA to reductively couple two [Fe_2_S_2_] clusters to form a [Fe_4_S_4_] cluster. Dre2 likely contains two [Fe_2_S_2_] clusters each coordinated by four cysteines.^23^ The Grx3/4[Fe_2_S_2_]Bol2 cluster is transferred to a heterotetrameric complex composed of CIA members **Cfd1** and **Nbp35**. Cfd1 and Nbp35 have cysteines that bind a [Fe_4_S_4_] cluster bridged between monomers.^14^ Nbp35 has additional cysteines that bind a second [Fe_4_S_4_] cluster. Thus, the (Cfd1)_2_(Nbp35)_2_ heterotetramer contains four [Fe_4_S_4_] clusters overall. Cfd1 binding may destabilize the bridging ISCs on Nbp35 to facilitate cluster transfer to clients. **Nar1**, another CIA agent that contains two [Fe_4_S_4_] clusters, helps transfer a [Fe_4_S_4_] cluster on the (Cfd1)_2_(Nbp35)_2_ heterotetramer to apo-protein targets.^24^

The CIA probably installs [Fe_4_S_4_] clusters into all of the [Fe_4_S_4_]-containing proteins found in the cytosol.^25^ Three such enzymes, including **Leu1, Glt1**, and **Met5**, are involved in amino acid biosynthesis. Leu1, isopropyl malate isomerase, is a [Fe_4_S_4_]-containing enzyme that helps synthesize leucine.^26,27^ Glt1, NADH-dependent glutamate synthase, is involved in glutamate biosynthesis.^28^ It contains two [Fe_4_S_4_] clusters and one [Fe_3_S_4_] cluster. The [Fe_3_S_4_] cluster is likely generated from a [Fe_4_S_4_] cluster that lost an iron ion.^29,30^ Met5 is the sulfite reductase subunit that contains a [Fe_4_S_4_] cluster coupled to a siroheme and is involved in methionine biosynthesis.^31–33^

Five cytosolic [Fe_4_S_4_]-containing proteins are involved in transcription and translation processes. **Rli1** contains two redox-inactive [Fe_4_S_4_]^2+^ clusters and functions to initiate translation and the biosynthesis of ribosomes.^34–^^36^**Elp3** is the catalytic subunit of the elongator complex, which modifies tRNAs in their wobble base position to regulate protein synthesis.^37–39^ Elp3 contains a radical-SAM [Fe_4_S_4_] cluster (coordinated by three cysteine ligands) that receives electrons from **Dph3**, a protein that contains a mononuclear Fe ion bound to four cysteines similar to that in rubredoxin.^40^ The **Dph1**:**Dph2** heterodimer catalyzes the first step in the synthesis of diphthamide, a modified histidine residue found in elongation factor 2.^41^ Like Elp3, the Dph1:Dph2 heterodimer contains a radical-SAM [Fe_4_S_4_] cluster, which receives electrons from Dph3.^42^ Dph3 also donates its iron to this cluster when the cluster is oxidatively deactivated into the [Fe_3_S_4_] form.^43^**Dph4** is a J-protein that stimulates and regulates the ATPase activity of Hsp70.^44^ Like Dph3, it binds a mononuclear Fe ion but whether it functions in diphthamide biosynthesis is uncertain. Dph4 may store iron (to avoid iron toxicity) or donate iron for cytosolic ISC assembly. **Tyw1** catalyzes the second step in wybutosine biosynthesis, which involves modifying Phe-tRNA. It contains two [Fe_4_S_4_] clusters, including a radical-SAM cluster that interacts with the other [Fe_4_S_4_] cluster that binds pyruvate.^45^**Ncs6**, a cytosolic protein involved in tRNA thiolation, contains an [Fe_3_S_4_] cluster.^46^**Lia1** contains a single Fe^II^ center that catalyzes the final step in hypusine biosynthesis, the hydroxylation of deoxyhypusine (post-translational modification of lysine) in translation initiation factor 5A.^47^**Dbr1**, a lariat debranching enzyme involved in intron degradation, contains an Fe^II^ center.^48^

**Rnr2** is the subunit of cytosolic ribonucleotide reductase, and it contains an Fe–O–Fe center.^49^ This enzyme synthesizes the deoxynucleotide triphosphates that are used in DNA replication and repair. **Bna1** is an Fe-containing cytosolic enzyme (3-hydroxyanthranilate 3,4-dioxygenase) that activates O_2_ and inserts both oxygen atoms into 3-hydroxyanthranilate.^50^ Bna1 is part of the kynurenine pathway, which is used in tryptophan degradation and *de novo* NAD biosynthesis. Its active site consists of an Fe^II^ ion coordinated by two histidine groups and one (bidentate) carboxylate group, a common motif of extradiol dioxygenases.

Hemes in yeast are exclusively synthesized in mitochondria, and are exported (via an unknown pathway) into the cytosol. The cytosol contains two heme proteins, namely **Ctt1** and **Yhb1**. Both protect the cell against oxidative damage. Catalase Ctt1 catalyzes the disproportionation of H_2_O_2_ and contains a high-spin Fe^II^ heme *b*.^51^ Yhb1 is nitric oxide (NO) dioxygenase (also called flavohemoglobin) that protects the cell against reactive nitrogen species. It does this by reacting NO with O_2_ and NADPH to generate harmless nitrate ions. Yhb1 also contains a HS Fe^II^ heme *b*.^52^ Catalase **Cta1** will be included in this group even though it is located in peroxisomes. It catalyzes the same reaction as Ctt1 and contains a similar active site heme *b* center.^53^

Five ferric reductases (**Fre1, Fre2, Fre3, Fre4**, and **Fre7**) are included in the list of cytosolic iron-containing proteins—even though Fre1 is located in the plasma membrane, and the locations of other ferric reductases are uncertain. All ferric reductases are presumed to contain a “bis-heme” center composed of two low-spin heme *b* centers in which each heme *b* is coordinated by two His axial ligands.^54^ A similar bis-heme motif is found in *b* cytochromes of mitochondria. **Tdh3** is glyceraldehyde-3-phosphate dehydrogenase. This glycolytic enzyme moonlights as a heme chaperone, delivering labile hemes to Hap1 (see following text) and other proteins. Although Tdh3 is present in the cytosol at high concentrations, only a tiny fraction binds cytosolic labile heme exported from mitochondria.^55^

Many iron-containing proteins in mitochondria are involved in respiration. Three respiratory complexes, including cytochrome *c* oxidase (cco), cytochrome bc_1_, and succinate dehydrogenase contain hemes and/or ISC centers. **Cox1** is the only iron-containing subunit of cco.^56,57^ It contains two heme *a* centers that are synthesized and installed by **Cox10** and **Cox15**.^58^**Mss51** is a heme-binding protein that regulates the installation of the heme *a* centers into cco, according to the availability of heme.^59^**Cob1, Cyt1**, and **Rip1** are iron-containing subunits of cytochrome bc_1_. Cob1 contains two low-spin heme *b* centers, including a high-potential b-562 (also called b_H_) and low potential b-566 (b_L_).^60^ Cyt1 contains 1 heme *c*, and Rip1 contains a Rieske [Fe_2_S_2_] cluster in which two of the four exoligands that coordinate the cluster are histidine rather than cysteine ligands.^61^**Sdh2, Sdh3**, and **Sdh4** are the iron-containing subunits of succinate dehydrogenase; Sdh2 houses one each of [Fe_4_S_4_], [Fe_3_S_4_], and [Fe_2_S_2_] clusters; a heme center bridges Sdh3 and Sdh4 subunits.^62^**Aco1** and **Aco2** are two isoforms of aconitase, an [Fe_4_S_4_]-containing enzyme of the citric acid cycle with a unique iron that binds substrate.^27,63^**Cyc1** and **Cyc7** are isoforms of cytochrome *c*, a heme c-containing protein that transfers electrons from cytochrome bc_1_ to cco.^64,65^**Cyb2** is L-lactate cytochrome *c* oxidoreductase, a heme *b* containing enzyme that catalyzes the oxidation of L-lactate to pyruvate while donating electrons to cytochrome *c*.^66^

Much of the cytosolic iron that flows into mitochondria is assembled into ISCs, a process requiring iron-containing proteins **Isu1, Isu2, Isa1, Isa2, Nfu1, Yah1**, and **Yfh1**.^67–71^ Isu1/2 are scaffold proteins on which [Fe_2_S_2_] clusters are assembled; they use iron donated either by Yfh1 or the LFeP in mitochondria (Fe^II^_mit_). Two [Fe_2_S_2_] clusters appear to be reductively coupled (using electrons transferred from [Fe_2_S_2_]-containing Yah1), to form [Fe_4_S_4_] centers on Isa1/2 proteins. With help from Nfu1, these clusters are installed into various apo-client proteins. [Fe_2_S_2_] clusters on Isu1/2 are transferred to **Grx5, Bol1**, and **Bol3**, and the resulting Grx5[Fe_2_S_2_]Bol1/3 heterodimers deliver these clusters to various client apo-proteins in the mitochondria, possibly including to the inner membrane protein Atm1, which is thought to export an iron–sulfur species to the cytosol.^72,73^ Grx5 binds [Fe_2_S_2_] clusters using cysteine and GSH; Bol1 binds the cluster via two His ligands.^69,74^

Mitochondrial enzymes **Bio2, Lip5**, and **Coq7** catalyze the synthesis of biotin, lipoic acid, and coenzyme Q, respectively.^75–77^ Bio2 and Lip5 contain ISCs while Coq7 contains a carboxylate-bridged Fe–O–Fe center. Thiazole synthase, **Thi4** and pyrimidine synthase, **Thi5**, are both involved in thiamine biosynthesis, and both contain mononuclear Fe^II^ centers.^78,79^ Two mitochondrial iron-containing enzymes help synthesize amino acids. **Lys4** (homoaconitase) contains a [Fe_4_S_4_] cluster and is involved in lysine biosynthesis.^80^**Ilv3** (dihydroxy-acid dehydratase) contains a [Fe_2_S_2_] cluster and helps synthesize valine.^81^**Aim32** contains a [Fe_2_S_2_] cluster with bis–his bis–cys coordination, localizes to the matrix and intermembrane space (IMS), and is involved in redox homeostasis.^22,82^

The only iron-containing protein in the heme biosynthetic pathway is ferrochelatase (**Hem15**), which installs Fe^II^ (donated by either Yfh1 or by Fe^II^_mit_) into porphyrin rings.^83^**Ccp1** (cytochrome *c* peroxidase) and **Exo5** help the cell respond to ROS. Ccp1 contains a heme *c* center and catalyzes the two-electron reduction of H_2_O_2_ (an unwanted by-product of respiration) along with the oxidation of cytochrome *c*.^84^ Exo5 is a mitochondrial exonuclease that helps repair mitochondrial DNA and contains an [Fe_4_S_4_] cluster.^85^ The only remaining iron-containing protein in mitochondria is **Fre5**, a heme-containing ferric reductase that has been located in mitochondria by a single proteomic study.^86^ Ferric reductases like Fre1 on the plasma membrane are used to reduce Fe^III^ → Fe^II^ but the physiological role of mitochondrial Fre5 is uncertain.

Nine iron-containing proteins in the nucleus contain a single [Fe_4_S_4_] cluster; all are coordinated by four standard cysteine exoligands.^87^ The cellular function(s) of these clusters is(are) largely unknown although the clusters are essential.^88^ DNA may conduct electricity, and protein-bound clusters may alter this conductivity to accurately position the proteins on the DNA for splicing.^89^ Alternatively, the clusters may make DNA replication and repair processes sensitive to the oxidative stress level of the cell (clusters are easily damaged by ROS).^90^**Chl1** is a DNA helicase involved in sister chromatid cohesion and DNA repair.^91^**Dna2** is a nuclease–helicase involved in DNA replication and recombination repair.^92^**Ntg2** is an endonuclease III that helps repair DNA oxidative damage.^93^**Pol1, Pol2, Pol3**, and **Rev3** are the catalytic subunits of DNA polymerase alpha, epsilon, delta, and zeta, respectively.^94,95^**Pri2** is the catalytic subunit of DNA primase that catalyzes the *de novo* synthesis of short RNA primers that are extended by DNA polymerases during the initiation of DNA replication.^96^**Rad3** is the XPD (superfamily of 2 DNA helicases with 5^′^-3^′^ polarity) helicase component of transcription factor IIH (a multiprotein transcription factor from Humans involved in both RNA polymerase II transcription and DNA repair) that helps initiate transcription and nucleotide excision repair.^97^**Tpa1** is one of three nuclear iron-containing proteins that do not contain a [Fe_4_S_4_] cluster. This enzyme is a non-heme Fe^II^ 2-oxoglutarate-dependent dioxygenase involved in DNA repair.^98^ The Fe^II^ ion is bound by three conserved residues including two histidine and an aspartic acid/glutamic acid residue (similar to that of Bna1).^99^**Hap1** and **Hap4** are involved in heme homeostasis, and both bind heme reversibly.^100,101^**Yap5** regulates iron import into the vacuole. It contains at least one [Fe_2_S_2_] cluster and a second cluster that is probably of the [Fe_4_S_4_] type.^102,103^ However, the second cluster is unstable and difficult to characterize.

Six iron-containing proteins in the ER are involved in the biosynthesis of ergosterol, an essential membrane component similar to cholesterol in mammals.^104^**Cyp51** (lanosterol- C14α-demethylase) is a cytochrome P-450 monooxygenase that is activated by **Cyb5**, cytochrome *b*5.^105,106^ Cyp51 is stabilized and activated by **Dap1**, a heme-binding protein.^107^**Erg25**, C4-methyl sterol oxidase and **Erg3** (sterol Δ5,6-desaturase) contain Fe–O–Fe centers.^108,109^**Erg5** (C-22 sterol desaturase) is a cytochrome P450.^110^

Four iron-containing proteins in the ER are involved in synthesizing fatty acids and sphingolipids. **Sur2** and **Scs7** are hydroxylases that contain Fe–O–Fe centers. Scs7 additionally contains a heme *b* center.^111^**Ole1** is Δ9 fatty acid desaturase that contains an Fe–O–Fe center as well as a cytochrome b5–binding domain.^112^**Mpo1** is a Fe^II^-dependent dioxygenase involved in fatty acid metabolism.^113^

Other iron-containing proteins in the ER are involved in the ROS signaling and the cell's response. **Yno1** is an NADPH oxidase that overproduces ROS in respiration-deficient cells.^114^**Grx6** is a class I monothiol located in the Golgi/ER. It binds an ISC and regulates the reduced-to-oxidized ratio of glutathione to protect the cell against ROS damage.^115^**Sfh5** is a membrane-associated heme protein that might be involved in heme trafficking or the ROS response.^116^**Hmx1** is heme oxygenase in the ER, which serves to bind and degrade heme groups.^117^**Fre8** is a ferric reductase found in the ER.^54^

Vacuoles play a major role in iron metabolism in that they store non-proteinaceous iron (see following text). However, the only iron-containing protein in this organelle appears to be **Fre6**, a ferrireductase that reduces non-proteinaceous high-spin Fe^III^ polyphosphate to the Fe^II^ state, which allows its export into the cytosol.^118^ This protein likely contains a bis-heme center similar to that in other Fre proteins.^54^

### Morphology of cells and organelles

Jorgensen *et al.* reported the median volume of fermenting haploid *S. cerevisiae* cells to be 42 ± 2 *×* 10^–15^ L.^119^ This volume was assumed in one proteomics study upon which our analysis relied, and so it was assumed here.^9^ Cell volume was initially divided into six compartments, including cell wall, cytosol, endoplasmic reticulum, mitochondria, nucleus, and vacuoles {*V*_cell_ = *V*_cyt_ + *V*_wall_ + *V*_mit_ + *V*_vac_ + *V*_nuc_ + *V*_er_}. Fractional volumes were defined as *f_i_* = *V_i_*/*V*_cell_, where the index *i* refers to one of these compartments. However, little if any iron-related chemistry occurs in the cell wall during exponential growth, and so this compartment was not considered further.^120^ Fractional volumes of fermenting yeast cells have been determined from 3D microscopic images, and they were considered in deciding on the fractional volumes used in this study (Supplementary Table S2).^121–124^

One complication is that the fractional volume of mitochondria, *f*_mit_, is greater in respiring cells than in fermenting ones. Early in exponential growth phase, fermenting cells are largely devoid of mitochondria, whereas in later stages, the organelle occupies ∼3% of cell volume.^125^ In respiring cells, mitochondria represent ∼10% of cell volume. Using GFP-labeled mitochondria Egner *et al.* found that mitochondria are a large tubular network.^126^ The network from fermenting cells is 6% thinner and branching points are four times fewer than in respiring cells. The overall surface area of the mitochondrial network in respiring cells is 2.8 ± 0.2 times larger than in fermenting cells. The mean volume of mitochondria in respiring cells is 3.0 ± 0.2 times larger than those in fermenting cells. Thus, the yield of isolated mitochondria from respiring cells should be ∼3× greater than from fermenting cells, which is approximately what we have observed (data not shown). We ultimately selected *f*_mit_ = 0.033 for fermenting cells and 0.100 for respiring ones.

### Calculating the cellular ironome and decomposition into organelle contributions

Ho *et al*. determined copies per cell (CPC) for most proteins in fermenting *S. cerevisiae* by integrating results from 21 independent proteomics studies of glucose-grown cells.^9^ Morgenstern *et al*. report CPCs for each mitochondrial protein in fermenting (F) and respiring (R) yeast.^127^ CPCs for each iron-containing protein P were converted into cellular concentration (in micromolar) using

(1) \begin{equation*} {[P]}_{cell} = \frac{{copies}}{{cell}}\frac{{1\,\,mol}}{{6.022 \times {{10}}^{23}\,\,copies}}\frac{{1\,\,cell}}{{42 \times {{10}}^{ - 15}L}}\frac{{{{10}}^6\,\,\mu mol}}{{1\,\,mol}} \end{equation*}

Results are listed in Supplementary Table S1. Local concentrations (within a cell compartment) were obtained by dividing cellular concentrations by the fractional volume associated with the compartment. For example, a mitochondrial protein from fermenting cells with 1 μM cellular concentration would have a local (i.e. mitochondrial) concentration of 30 μM (*f*_mit(F)_ = 0.033; 1 μM/0.033). Protein expression levels were presumed to be 3× higher in R than in F cells, due to a 3× higher fractional volume of mitochondria in respiring yeast cells. Thus, the cellular concentration of the same protein P in respiring cells would be 3 μM, whereas its local concentration would remain 30 μM (illustrated in Supplementary Fig. S1).

Supplementary Table S3 lists the iron center(s) found in each iron-containing protein along with the contribution that each protein makes towards the overall iron concentration in the cell. Eight types of centers were considered, including [Fe_4_S_4_], [Fe_3_S_4_], [Fe_2_S_2_] clusters, high- and low-spin hemes, mononuclear iron centers with primarily sulfur ligands, mononuclear iron centers with primarily O/N ligands, and Fe–O–Fe dimers. The concentration of iron associated with each center was estimated by multiplying the concentration of the protein by the number of irons in the center.

**Fig. 1:**
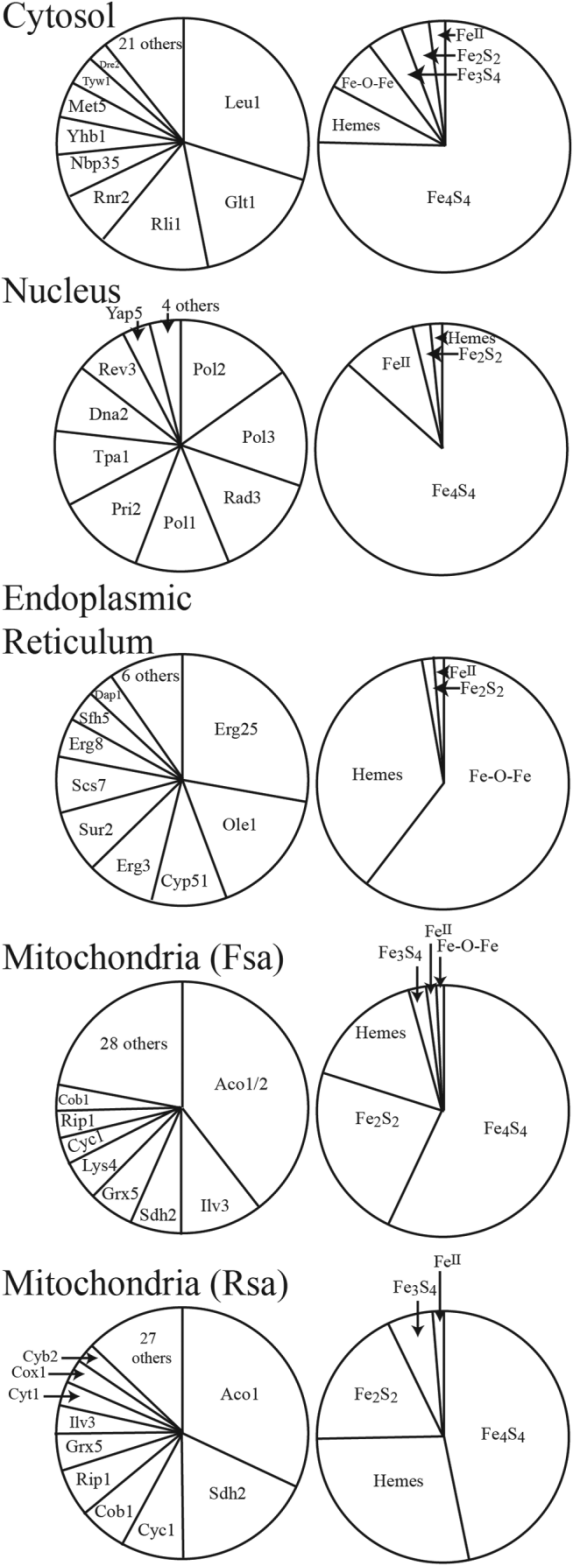
Pie-chart distributions of iron in each compartment of a yeast cell. Charts on the left indicate the major proteins in each considered compartment, given as a percentage of iron contribution. Charts on the right show the percentage of different types of iron centers in the compartment, again as a percentage of iron contribution.

Our analysis indicated that the cytosol of both F and R cells grown in iron-replete media should contain 60 μM of protein-bound iron. Approximately 72% of this should be in the form of [Fe_4_S_4_] clusters, with hemes and Fe–O–Fe centers each contributing ca. 7% of the iron. See Fig. 1 for pie-chart distributions. [Fe_3_S_4_] and [Fe_2_S_2_] clusters collectively contribute another 8%, followed by mononuclear Fe^II^ (2%). More than 60% of cytosolic protein-bound iron should be associated with just three proteins, namely Leu1 (30%), Glt1 (17%), and Rli1 (14%), all of which contain [Fe_4_S_4_] clusters. Leu1 and Glt1 are involved in amino acid biosynthesis while Rli1 is involved in ribosome biosynthesis.

The protein-bound iron concentration in the nucleus should be ∼30 μM. Of this, 87% should be [Fe_4_S_4_] clusters and 10% non-heme high-spin Fe^II^ ions. The remaining few percentages should be hemes and [Fe_2_S_2_] clusters. Protein-bound iron in the nucleus distributes rather evenly among eight proteins all of which are involved in DNA replication and repair.

The ER should contain ∼460 μM of protein-bound iron. This local concentration is surprisingly high, perhaps because the absolute amount of protein-bound iron in the ER is minuscule on a per cell basis. The ER is essentially devoid of ISCs (apart from Grx6, which may actually be located in the Golgi apparatus). Sixty percentage of protein-bound iron in the ER should be [Fe–O–Fe] centers; 37% should be hemes (Fig. 1 and Supplementary Table S3). Over half of protein-bound ER iron should be found in three proteins, namely Erg25, Ole1, and Cyp51, but other proteins should make significant contributions. Vacuoles should contain virtually no proteinaceous iron (and so no pie charts are shown). This was unexpected because the organelle plays a major role in cellular iron metabolism by storing the metal. However, the iron is stored in a non-proteinaceous form (see following text) and so was not included at this stage of analysis.

The situation was more complicated for mitochondria, in that the iron content of the organelle isolated from F cells differs from that isolated from R cells. For example, Morales *et al*. reported 8 and 30 μM for the concentration of cytochrome *c* oxidase in mitochondria isolated from F and R cells, respectively.^128^ The averaged Cox1 local concentrations predicted by Ho *et al*. and Morgenstern *et al*. were 2 and 4 μM for F and R conditions, respectively.^9,127^ We attempted to redress this discrepancy by averaging the subunit concentration for the respiratory complex. Cytochrome *c* oxidase consists of one copy of each subunit listed in Supplementary Table S4 except for Cox8 for which two copies are included. Thus, in principle the concentration of each subunit (and Cox8 divided by 2) ought to be the same assuming that no subunits are in excess. The average local concentration of all cytochrome *c* oxidase subunits was 8 and 11 μM for F and R conditions, respectively, closer to the values reported by Morales.^128^ This suggested that it would be more accurate to employ the average concentration of all cytochrome *c* oxidase subunits, not just that of Cox1, in estimating the concentration of the respiratory complex, and so Fsa and Rsa (sa = subunit averaged) concentrations for cytochrome *c* oxidase were used in Supplementary Table S3. We also used subunit-averaged concentrations for cytochrome bc1 and succinate dehydrogenase.

Calculated concentrations for Fsa and Rsa mitochondria were 1030 and 750 μM, respectively. Observed concentrations range from 480 to 840 μM for F, and from 690 to 840 μM for R (Supplementary Table S5). Thus, calculated and observed concentrations for R mitochondria agreed well, whereas that calculated for F cells were higher than observed. One contributor to this difference may have been our assumption that 100% of aconitase is metallated with an [Fe_4_S_4_] cluster. Aconitase is the most highly expressed iron-containing protein in mitochondria, representing nearly 40% of mitochondrial iron in F cells. However, at least 9% of aconitase in mitochondria is unmetallated.^129,130^ Including this assumption would have lowered the calculated Fsa mitochondria concentration below 1000 μM. Another correction that would have moved the calculated Fsa mitochondrial iron concentration in the “wrong” direction would have been to include the presence, in MB spectra of isolated F mitochondria, of “unassigned iron” (likely from non-proteinaceous nanoparticles), as well as a pool of non-proteinaceous non-heme high-spin (NHHS) Fe^II^, which is especially prevalent in F mitochondria.^128^ Neither correction was included in Supplementary Table S5 calculations.

The last adjustment made in our calculations was to include contributions from two non-proteinaceous LFePs, including a high-spin Fe^III^ pool in vacuoles and a non-heme high-spin Fe^II^ pool in cytosol. Including these pools rendered the calculated iron concentrations in F and R whole cells similar to observed concentrations (Supplementary Table S5). Simulated whole-cell iron concentrations were 300 μM and 340 μM for Fsa and Rsa cells, respectively, whereas observed concentrations ranged from 400 to 470 μM for F and 480 to 800 μM for R cells.

### Redox status of iron centers

Most iron centers in biology are redox active, and the corresponding MB spectrum will differ according to oxidation and spin state. To simulate the MB spectrum of a center, these properties must be estimated, including the concentration of reduced and oxidized states [(red) and (ox)]. If the standard thermodynamic reduction potential of each center *E*^0^′_center_ (at the pH of the cellular compartment) and the reduction potential of the compartment (*E*′_compartment_) were known, the Nernst equation {*E*_compartment_ = *E*^0^_center_^ −^ (RT/nF)ln{[red]/[ox]} could be used to determine the percentage of centers in each redox state—presuming that all the redox centers involved are in equilibrium with the redox buffers in their cellular compartment.^131^ In practice, the redox components of exponentially growing cells are not at equilibrium. Moreover, *E*^0^′_center_ have not been determined for every redox center. Thus, the percentages of [ox] and [red] for some centers had to be estimated for MB spectra to be simulated.

Due to the high cellular concentrations of glutathione (c-glutamylcysteinylglycine = GSH) (∼4.0 mM) and the oxidized disulfide GSSG (∼0.052 mM), the apparent redox potential of a compartment is largely dictated by the GSH/GSSH couple; *E*^0^′_GSSG/GSH_ ≈ −240 mV vs. NHE at pH 7.^131–133^ [GSH]/[GSSG] ratios have been determined for various cellular compartments, and they have been used (via the Nernst equation) to estimate *E*′_compartment_. *E*′_compartment_ is undoubtedly influenced by other redox couples, including the NADP^+^/NADPH and NAD^+^/NADH (≈ −315 mV). Also, since living systems are not at equilibrium, apparent redox potentials may also be influenced by the rates of various redox reactions, e.g. involved in the catabolic oxidation of nutrient carbon. Thus, the apparent redox potential of a compartment may vary according to whether the cell is respiring or fermenting. However, quantifying these influences is beyond the scope of this study.

Although different values for each compartment have been reported, we assumed those in Supplementary Table S6. We assumed −380 mV for the reduction potential of peroxisomes to estimate the oxidation state of Cta1. It is difficult to reconcile a potential of −255 mV for the IMS (based on the GSSG/GSH couple) with the redox reactions associated with the electron transport chain, e.g. involving cytochrome *c* (whose redox potential is +235 mV or thereabout). The free-energy change for the reaction {2Cytc(Fe^III^) + 2GSH → 2Cytc(Fe^II^) + GSSH} is ca. −90 kJ/mol, hugely downhill. That reaction competes with the redox reaction of cytochrome *c* with cytochrome bc_1_ (*E*^0^ = +285 mV for Rip1) and with cytochrome *c* oxidase (*E*^0^ = +255 mV for heme a) in which the overall free-energy change is minor. Perhaps the electron transfer kinetics are faster for the reactions involving minor free-energy changes and slower for the reaction with GSH/GSSG. In any event, for this paper, we relied mostly on the GSH/GSSG couple for assigning redox and MB properties for each iron protein in yeast (Supplementary Table S7).

### MB simulations of cytosol and isolated organelles

**Fig. 2:**
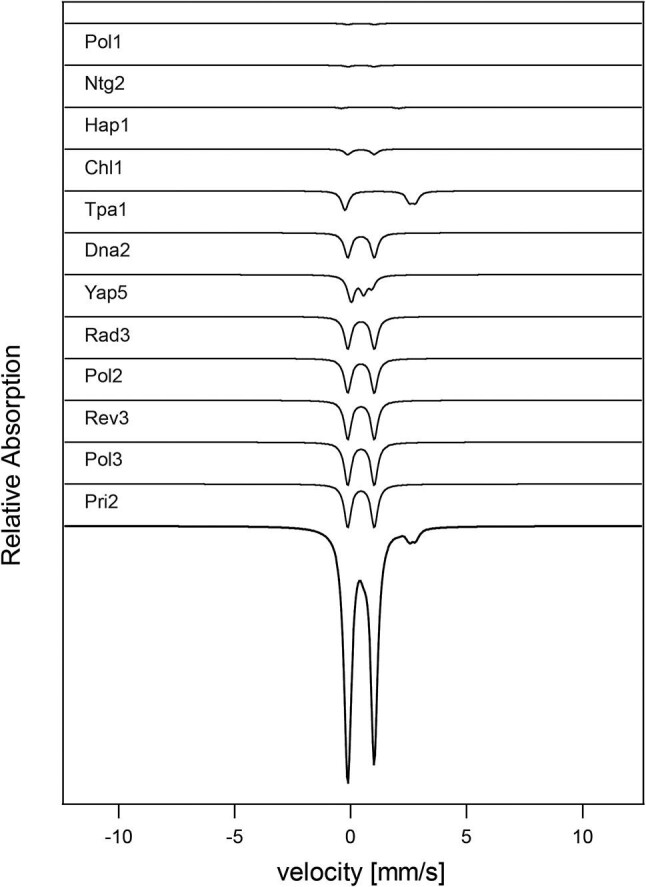
Simulated Mössbauer spectra of nuclei from *S. cerevisiae*. Temperatures of 4.2–5 K and parallel applied fields of ∼0.05 T were assumed (same for all figures). Individual spectra for each iron-containing protein in the nucleus, as indicated, were simulated and summed to generate the predicted spectrum (bottom) of isolated nuclei.

**Fig. 3:**
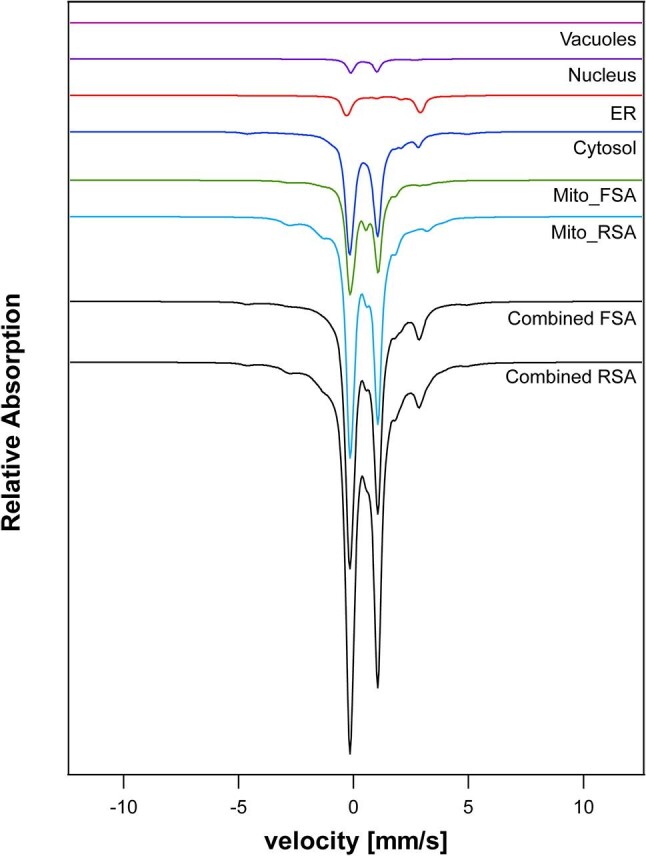
Simulated Mössbauer spectra of each cellular compartment and whole Fsa and Rsa yeast cells. Spectra were generated by summing the spectra from each of the five cellular compartments, weighted by the fraction of cellular iron in each compartment.

We next simulated the MB spectrum expected for each iron center in each iron-containing protein, and summed the simulations weighted according to the concentration of iron in each protein as given in Supplementary Table S3. We initially simulated the spectrum for each of the five isolated cellular compartments, as shown in Fig. 2 for the nucleus. Simulated spectra for this and three other compartments (cytosol, endoplasmic reticula, and vacuoles) are shown in Fig. 3. The iron contents of these compartments were presumed to be invariant to metabolic growth mode. In contrast, the iron content of mitochondria depends on whether cells are grown under fermenting or respiring conditions. Simulated spectra of mitochondria for these two metabolic states are shown in Fig. 3 (black lines). Spectral contributions and percentages for individual mitochondrial proteins are given in Supplementary Fig. S2 and Table S8. The simulated spectrum of fermenting mitochondria exhibited less intense central quadrupole doublet, relative to respiring mitochondria. These simulations should be compared to the experimental spectra shown in Supplementary Fig. S3. The simulated spectrum of F mitochondria should exhibit less resolution between the two “legs” of the central doublet. This may not have been evident in simulated spectra for three reasons. First, we did not include “unassigned” iron in calculating the iron content of F mitochondria. This material afforded residual spectral features that lacked sufficient resolution to allow assignment. Since this iron contributes spectral features between the two legs of the central doublet, its absence in simulations afforded greater resolution than is actually observed. Another contributing factor, mentioned earlier, may be that a significant fraction of aconitase in F mitochondria may not be metallated. And finally, the calculated concentration of [Fe_2_S_2_] clusters was somewhat higher than has been reported experimentally. Such clusters in the 2+ core oxidation state may have contributed to the unassigned iron.

Experimental spectra include 2–7% high-spin Fe^II^ hemes and 2–30% NHHS Fe^II^ (Supplementary Table S5) whereas simulations included ∼2% of each. The discrepancy with NHHS Fe^II^ is due to not including the labile Fe^II^ pool in simulations that has been detected experimentally.^11,128,134^ Morales *et al.* and Holmes-Hampton *et al*. report a similar percentage for fermenting mitochondria (but substantially less for respiring mitochondria). Some of the NHHS Fe^II^ in fermenting mitochondria is due to the LFeP in the organelle.^11^

### MB simulations of whole cells

**Fig. 4:**
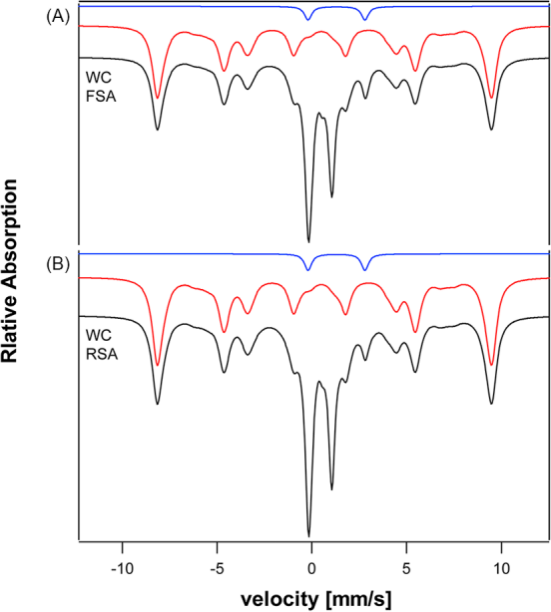
Simulated Mössbauer spectra of whole yeast cells with non-proteinaceous iron included. Simulations of: non-proteinaceous non-heme high-spin Fe^II^ (blue line); non-proteinaceous high-spin vacuolar Fe^III^ (red); iron-replete fermenting whole cells (upper black); and respiring (Rsa) whole cells (lower black).

We next simulated the MB spectrum of whole yeast cells grown under fermenting and respiring conditions. This included contributions from each organelle summed according to the concentration of iron contributed. The resulting iron-replete whole-cell simulated spectra in Fig. 3 (black lines, combined Fsa and Rsa) should be compared to the experimental spectra in Supplementary Fig. S4, B and C. The greatest difference was the lack of a dominant magnetic feature arising from *S* = 5/2 Fe^III^ species in the simulated spectrum. Reconciling this required that we included contributions from non-proteinaceous forms of iron. Cockrell *et al*. reported that isolated vacuoles from iron-replete cells exhibit a magnetic MB spectrum typical of high-spin *S* = 5/2 due to Fe^III^ ions bound to polyphosphate chains in the organelle, and subsequent chromatographic evidence for this has been reported.^5,12^ This feature was simulated and included at ∼60% spectral intensity in Fig. 4.

One objective of our study was to estimate the collective cellular concentration of LFePs in yeast. Such pools are present in the cytosol and mitochondria of yeast cells, as detected them using liquid chromatography.^6,11,12,135^ Other cellular compartments, including vacuoles, nuclei, and ER may also contain LFePs. There is substantial but indirect evidence that these pools consist of non-heme high-spin Fe^II^ complexes. Here we had the opportunity to assess the concentration of LFePs in whole intact yeast cells. Experimental spectra of iron-replete cells include a quadrupole doublet due to NHHS Fe^II^, which represents 5–11% of overall spectral intensity.^136^ According to Supplementary Table S3, ∼9 μM of this Fe should be protein-bound, including mononuclear NHHS Fe^II^ plus Fe–O–Fe species presumed to have irons in the ferrous state. This would correspond to ∼3% of cellular iron. In this estimate, the remaining 2–8% of NHHS Fe^II^ (10–40 μM) might correspond to the collective LFePs (in cytosol, mitochondria, etc.), with the majority being cytosolic. The simulated *S* = 2 quadrupole doublet in Fig. 4 reflects this approximate concentration (15 μM; blue line) for the LFeP. Concentrations of the LFeP as low as 10 μM and as high as 50 μM seem possible. The simulation parameters (δ = 1.31 mm/s; Δ*E*_Q_ = 3.0 mm/s) are typical of high-spin Fe^II^ complexes with primarily O/N ligands. We can exclude from consideration Fe^II^ complexes with mainly or exclusively coordinated with sulfur donors. With these modifications, simulated and experimental whole-cell spectra were similar (compare Fig. 4, Fsa and Rsa, to Supplementary Fig. S4B and C).

### Characterization of nuclei

**Fig. 5:**
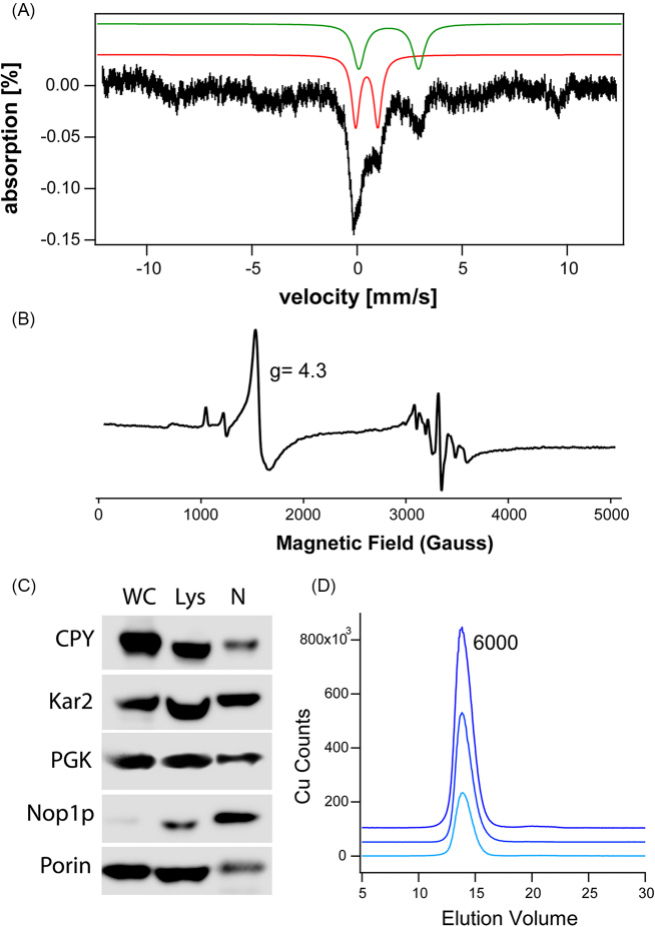
Characterization of yeast nuclei. **A**, Averaged Mössbauer spectrum of isolated nuclei from growth media containing 1 or 40 μM Fe. Solid lines are simulations of the CD (red), NHHS Fe^II^ (green), and NHHS Fe^III^ (teal). **B**, X-band EPR spectrum (5K, modulation amplitude—10 G, modulation frequency—100 kHz, power— 0.2 mW, frequency—9.36 GHz, and sweep time—300 s) of nuclei isolated from cells grown with 1 μM iron. **C**, Western blot of whole-cell (WC), lysate (Lys), and nuclei (N) against various antibody markers for CPY (vacuoles), Kar2 (ER), PGK (cytosol), Nop1 (nuclei), and porin (mitochondria). D, ^65^Cu-detected chromatograms of flow-through solution from soluble nuclear extracts from three independent preparations collected using LC-ICP–MS.

Previously we have isolated and characterized mitochondria, vacuoles, and cytosol from yeast cells, but not nuclei. Here, we report a preliminary characterization of nuclei isolated from yeast cells grown under low (1 μM) and high (40 μM) Fe concentrations in the growth media. Fluorescence microscopic images of isolated nuclei are given in Supplementary Fig. S5. A western blot of isolated nuclei (Fig. 5B) confirms significant purification with minor contamination of cytosol, vacuoles, and mitochondria. The average metal concentrations and uncertainties for five independent preparations were (in micromolar): Fe, 35 ± 15; Cu, 50 ± 20; Mn, 3 ± 2, and Zn, 35 ± 10. The Fe concentration was similar to that estimated by our bioinformatic approach (27 μM; Supplementary Table S3). Figure 5A shows the averaged MB spectrum of isolated nuclei from four independent batches. Individual spectra are given in Supplementary Fig. S6. We present the average here because the individual spectra were noisy due to the extremely low iron concentrations (despite collecting each spectrum for over 300 hr). The dominant feature was a quadrupole doublet with parameters typical of [Fe_4_S_4_]^2+^ clusters (δ = 0.42 mm/s, Δ*E*_Q_ = 1.0 mm/s). Also evident was a doublet typical of non-heme-iron Fe^II^ species bound primarily to O/N ligands (δ = 1.3 mm/s, Δ*E*_Q_ = 2.7 mm/s). The low-iron spectra included a magnetic feature typical of high-spin Fe^III^ ions (*D* = 0.5 cm^–1^, *E*/*D* = 0.33, Δ*E*_Q_ = 0.3 mm/s, *A_xyz_* = 31 MHz, δ = 0.55 mm/s). Consistent with that, an EPR spectrum of isolated nuclei exhibited a *g* = 4.3 EPR signal (Fig. 5B). Extracts of isolated nuclei were passed through a 10 kDa cutoff membrane. LC–ICP–MS analysis of the flow-through solution exhibited variable low-molecular-mass iron peaks (not shown) as well as a dominant copper peak at ca. 6000 Da (Fig. 5D). The apparent mass suggests that the peak might arise from copper metallothionein Cup1 but further studies are required to verify this. The possibility of a redox-active pool of labile Fe^II^/Fe^III^ in the nucleus should also be considered.

## Discussion

In this study, we developed a semi-quantitative model of the entire iron content of yeast cells grown under respiring and fermenting iron-replete conditions. This ironome model was developed by manually cataloguing all 100 known iron-containing proteins in yeast cells as described in the literature. The expected MB spectrum for each protein was predicted from the type of iron center present, the known MB-relevant parameters, and the known redox properties of the centers. The concentration of each protein was extracted from published mass spectrometry–based quantitative proteomics of yeast cells in which copy numbers per cell were calculated for each protein in fermenting and respiring yeast. Results include a look-up table of all iron-containing proteins in these cells, including both cellular and local concentrations for both fermenting and respiring metabolic states.

This is the second cataloguing of the iron-containing proteins in *S. cerevisiae*. The original list contained 144 proteins, including some that did not contain iron but were intimately involved in iron trafficking or regulation, and a few that were misassigned as being iron-containing (see Supplementary Table S1 legend).^3^

Simulated spectra based on our current model were compared to experimental spectra of fermenting and respiring iron-replete whole cells. The major difference was the absence of spectral features due to non-proteinaceous vacuolar Fe^III^ in the simulations. To reconcile this, we added such a feature. If vacuoles were empty (e.g. as expected for cells grown on 1 μM ^57^Fe), the total predicted fermenting and respiring cell concentrations would be 82 and 123 μM, respectively. Experimental cellular iron concentrations between 170 and 250 μM have been reported for iron-deficient fermenting and respirofermenting conditions.^136^

The model also predicts that the total concentration of hemes in fermenting and respiring cells should be 11 μM and 26 μM, respectively (Supplementary Table S3). Hanna *et al*. reported that the total heme concentration in fermenting yeast is 1.2–6.2 μM, determined using a fluorometric assay.^137^

Our analysis supports previous results that the fractional volume of mitochondria in respiring yeast cells (and the expression level of all mitochondrial proteins) are about 3× higher than in fermenting cells; representing 10% and 3.3% cellular volume, respectively. During respiration, the expression levels of respiration-related proteins appear to be boosted; 44% for cytochrome *c* oxidase, 32% for cytochrome bc1, and 98% for succinate dehydrogenase. Experimentally, Morales *et al*. report respiration-related boosts of ca. 200%.^128^

Hudder *et al*. also used CPC data to estimate iron concentrations in mitochondria, but calculated an overall protein-bound iron concentration of just 265 μM for the isolated organelle.^10^ Different assumptions were made regarding cell volume and fractional volume of mitochondria. If current assumptions were used, the Hudder concentrations would increase by 2.4×, affording a more comparative mitochondrial iron concentration (630 μM) to what was calculated in the current study (750 μM, Supplementary Table S5). This illustrates the sensitivity of modeling assumptions in estimating the iron concentration in the organelle.

The current study also provides estimates of non-proteinaceous iron pools in cells. The estimated spectral contribution due to non-proteinaceous non-heme high-spin Fe^II^ pool suggested a concentration of just 10–50 μM. Previous estimates of the size of LFeP pools were based on quantifying fluorescence quenching of chelator probes or back-calculating from the intensity of calibrated chromatography peaks obtained from diluted cell lysates. We are unaware of any chelator-based determinations of the LFeP in yeast cells, but in mammalian cells, LFeP concentrations range from 1 to 10 μM.^138^ The LFeP of WT yeast cytosol was measured directly by ICP–MS to contain 66 μM Fe in one study and ∼50 μM Fe in another (when cells were grown under low-iron conditions).^139,12^ These concentrations are on the high-end of current estimates. The possibility that some of the previously reported iron in the detected LFeP arose from vacuolar iron contamination during cytosol isolation cannot be excluded. Compared to these other methods, the bioinformatics/spectroscopy-based estimates provided here for the LFeP in *S. cerevisiae* cells refer to unperturbed intact whole cells.

Additionally, we have isolated and characterized nuclei from respiring yeast cells grown under low and high iron conditions. This was the first such study and further work is required to establish the iron content in this organelle. Nevertheless, the four MB spectra collected from independent batches all were dominated by a quadrupole doublet arising from *S* = 0 [Fe_4_S_4_]^2+^ clusters, as predicted by our bioinformatics analysis. Also observed were features arising from NHHS Fe^II^ and Fe^III^, which suggest redox-active mononuclear iron species in nuclei. These features were not predicted by our bioinformatic analysis, and further studies are required to establish their origins.

Given the extraordinary number of moving parts needed to construct this model, it is not surprising that the predictions generated by it are not fully realized experimentally. Nevertheless, the model provides realistic constraints on a wide range of critical parameters, including the dimensions of the cell and fractional volumes of the major cellular compartments, the absolute (micromolar) concentrations of iron in each major compartment of the cell, the absolute (micromolar) cellular concentration of each iron-containing protein in the cell, the redox properties of these compartments and each iron-containing species housed therein, and the size of the LFePs in the cell. Researchers in the field are encouraged to communicate corrections or additions to the authors. With time and continued efforts to update relevant data and assumptions, future models will gradually sharpen in terms of accuracy, enhancing their predictive power and thus their utility for research.

## Conclusions

The major conclusions of this study are as follows.

- *Saccharomyces cerevisiae* houses ∼100 iron-containing proteins. The name of each protein, type of iron center, and cellular location in the cell are catalogued in Supplementary Table S1. The concentration of each iron-containing protein was estimated from reported quantitative mass spectrometry data along with morphological details regarding cell volume and fractional volume of major organelles.
- The type of iron center and redox properties for each such protein was catalogued in Supplementary Tables S3 and S7. This information was used to simulate the expected MB spectra of each iron-containing protein in a yeast cell. Composite spectra for each major organelle and for entire cells under fermenting and respiring conditions were obtained.
- Mitochondria are affected by whether cells are grown under fermenting or respiring conditions. Respiring cells contain ∼3× more mitochondria than fermenting cells. The concentrations of respiration-related proteins are different in respiration conditions.
- Fractional cellular volumes of fermenting and respiring mitochondria (*V*_mit_/*V*_cell_) are ∼0.03 and 0.10, respectively (for strain W303 grown aerobically in minimal media).
- The [Fe_4_S_4_] cluster in aconitase is the dominant contributor to the MB spectra of isolated mitochondria.
- The major LFeP in iron-replete cells was composed of non-heme high-spin Fe^III^ species located in vacuoles. The cytosolic NHHS Fe^II^ pool only contributed a few percentage points to the MB spectra, suggesting a pool size between 10 and 50 μM.
- Nuclei were isolated and preliminary MB spectra were obtained; they were dominated by [Fe_4_S_4_]^2+^ clusters as predicted. Non-heme high-spin Fe^II^ and Fe^III^ were also observed; their origins need to be investigated.

## Supplementary Material

mfac080_Supplemental_File

## Data Availability

The data underlying this article are available in the article and in its online supplementary material.
