# Supplementary material for: Mössbauer-based molecular-level decomposition of the Saccharomyces cerevisiae ironome, and preliminary characterization of isolated nuclei
Source: Metallomics. 2022 Oct 10;14(11):mfac080. doi: 10.1093/mtomcs/mfac080 (PMC9624242; doi:10.1093/mtomcs/mfac080)

**Electronic Supplementary Information**

For “Mössbauer-based molecular-level decomposition of the *Saccharomyces cerevisiae* ironome…” by Lindahl and Vali, 2022

Table of Contents:

Table S1: Iron-containing proteins in *S. cerevisiae* including copies-per-cell, and cellular and local concentrations.

Table S2: Fractional Volumes of yeast cellular compartments.

Figure S1: Illustration of F, R, Fsa, and Rsa assumptions.

Table S3: Iron centers contained in each iron protein and their contribution to the ironome.

Table S4: Local subunit-average concentrations of selected mitochondrial proteins (iron-containing respiratory complexes) under F and R conditions.

Table S5: Assumed thermodynamic reduction potentials for various cellular compartments.

Table S6: Assumed Mössbauer and redox properties of each iron center in yeast.

Table S7: Local Fe concentrations and fractional contributions for individual mitochondrial proteins assuming Fsa and Rsa concentrations.

Table S8: Comparison of simulated vs. observed parameters.

Figure S2: Simulated spectrum of Rsa mitochondria showing individual contributions.

Figure S3: Experimental spectra of isolated mitochondria from *S. cerevisiae*.

Figure S4: Experimental spectra of whole-cell *S. cerevisiae*.

Figure S5: Fluorescence microscopic images of isolated nuclei.

Figure S6: Mössbauer spectra of individual batches of isolated nuclei.

**Table S1: Iron-containing proteins in *S. cerevisiae* including copies-per-cell and cellular and local concentrations (in µM).** Copies per cell (CPC) are reported as the $\sqrt{(mean CPC)(median CPC)}$using values from Ho et al. for fermenting cells (grown on glucose) [9]. CPC values from fermenting (F) and respiring (R) yeast cells were also used [127]. Fermenting values were averaged. Ho et al. did not obtain CPCs for Fre2 and Fre3 so the average CPC values for Fre1, Fre4, Fre5, Fre7, and Fre8 were used. CPC for Cob1 was also not provided, so the average CPC of Cyt1 and Rip1 was used. The concentration of Cox10 was presumed to be 1/8^th^ of the concentration of Cox15, as reported [140]. Cta1 is located in peroxisomes but is included with cytosol proteins. Grx6 and Sfh5 are included with ER proteins even though Grx6 is likely located in the Golgi, and the location of Sfh5 is only known to be membrane-associated.

The list represents an update of a published list [3]. Iron-containing proteins Apd1, Aim32, Exo5, Lia1, Ncs6, Sfh5, Tdh3, Thi4, and Thi5 were added. Seven cell-wall proteins that had been included previously, namely Fit1, Fit2, Fit3, Arn1, Arn2, Arn3, and Arn4, were excluded due to uncertainty whether any of them bind iron. Also excluded were Ade4, Coq6, Cyt2, Cyc3, Dld1, and Met8. Ade4 encodes phosphoribosylpyrophosphate amidotransferase which in *Bacillus subtilis* and mammals contains an [Fe_4_S_4_] cluster. The same had been assumed for Ade4 in yeast. However, Mantsala and Zalkin found that the yeast enzyme, like that of *E coli*, lacks the cysteine residues used to coordinate the cluster in the other homolog enzymes [141]. Thus, Ade4 probably does not contain an ISC. Coq6 is an FAD-dependent monoxygenase that catalyzes a hydroxylation reaction that requires ferredoxin (Yah1) and ferredoxin reductase (Arh1). Such reactions are often catalyzed by cytochrome P450 enzymes, and CoQ6 had been assumed^3^ to be such an enzyme. However, evidence for this is lacking [142, 143]. Cyt2 and Cyc3 are cytochrome c1 and c heme lyases. These enzymes help attach the heme to cytochrome c containing proteins, but have a redox function rather than one involving heme binding [144]. Dld1 (D-lactate ferricytochrome c oxidoreductase or D-lactate dehydrogenase), contains a flavin and a zinc ion but not a b2 cytochrome as is found in L-lactate dehydrogenase [145]. Met8 was presumed to contain a siroheme iron. This homodimeric enzyme catalyzes the NAD-dependent dehydrogenation that yields sirohydrochlorin, and it catalyzes the chelation of Fe^II^ to give siroheme [146]. The enzyme was incubated in MnCl_2_ and CoCl_2_ but no metal binding site was observed in the X-ray crystal structure. Thus, the resting enzyme probably contains little iron.

| Number | Name | Identifier | CPC | [P]_cell(F)_ (µM) | [P]_local_ (µM) |
| --- | --- | --- | --- | --- | --- |
|  | **Cytosol** |  |  |  | [P]_cyt(F)_ = [P]_cyt(R)_ = [P]_cell(F)_ ÷ 0.615 |
| 1 | Aft1 | YGL071W | 2568 | 0.1015 | 0.1651 |
| 2 | Aft2 | YPL202C | 262 | 0.0104 | 0.01684 |
| 3 | Apd1 | YBR151W | 4728 | 0.1869 | 0.3040 |
| 4 | Bna1 | YJR025C | 7561 | 0.2989 | 0.4861 |
| 5 | Bol2 | YGL220W | 3262 | 0.1290 | 0.2097 |
| 6 | Cfd1 | YIL003W | 1515 | 0.0599 | 0.09740 |
| 7 | Cta1 (peroxisomes) | YDR256C | 1242 | 0.0491 | 0.07984 |
| 8 | Ctt1 | YGR088W | 5783 | 0.2286 | 0.3718 |
| 9 | Dbr1 | YKL149C | 1545 | 0.0611 | 0.0993 |
| 10 | Dph1 | YIL103W | 2898 | 0.1146 | 0.1863 |
| 11 | Dph2 | YKL191W | 3518 | 0.1391 | 0.2262 |
| 12 | Dph3 | YBL071W-A | 8829 | 0.3491 | 0.5676 |
| 13 | Dph4 | YJR097W | 1975 | 0.0781 | 0.1270 |
| 14 | Dre2 | YKR071C | 4827 | 0.1908 | 0.3103 |
| 15 | Elp3 | YPL086C | 4680 | 0.1850 | 0.3009 |
| 16 | Fre1 | YLR214W | 2144 | 0.0848 | 0.1378 |
| 17 | Fre2 | YKL220c | 1277 | 0.05050 | 0.08211 |
| 18 | Fre3 | YOR381w | 1277 | 0.05050 | 0.08211 |
| 19 | Fre4 | YNR060w | 1141 | 0.04511 | 0.07335 |
| 20 | Fre7 | YOL152w | 120 | 0.00474 | 0.00771 |
| 21 | Glt1 | YDL171C | 14158 | 0.5598 | 0.9102 |
| 22 | Grx3 | YDR098C | 7999 | 0.3163 | 0.5142 |
| 23 | Grx4 | YER174C | 4260 | 0.1684 | 0.2738 |
| 24 | Leu1 | YGL009C | 68365 | 2.703 | 4.395 |
| 25 | Lia1 | YJR070C | 21185 | 0.8376 | 1.362 |
| 26 | Met5 | YJR137C | 8489 | 0.3356 | 0.5457 |
| 27 | Nar1 | YNL240C | 1419 | 0.05610 | 0.0912 |
| 28 | Nbp35 | YGL091C | 8349 | 0.3301 | 0.5368 |
| 29 | Ncs6 | YGL211W | 2647 | 0.1047 | 0.1702 |
| 30 | Rli1 | YDR091C | 16173 | 0.6394 | 1.040 |
| 31 | Rnr2 | YJL026W | 31412 | 1.242 | 2.019 |
| 32 | Tdh3 | YGR192C | 1011608 | 40.00 | 65.035 |
| 33 | Tyw1 | YPL207W | 4385 | 0.1734 | 0.2819 |
| 34 | Yhb1 | YGR234W | 44442 | 1.757 | 2.857 |
|  |  |  |  |  |  |
|  | **Mitochondrion** |  | **Copies per cell**  (Fermenting, Ho et al.^9^ = “Ho”  (Fermenting and respiring, Morgenstern et al.^127^ = “Mo” | **Cell Concentration**  [P]_cell(F)Ho_  [P]_cell(F)Mo_  [P]_cell(F)Ave_  [P]_cell(R)Mo_ | **Local Concentration**  [P]_mit(F)Ho_ = [P]_cell(F)Ho_ ÷ 0.033  [P]_mit(F)Mo_ = [P]_cell(F)Mo_ ÷ 0.033  [P]_mit(F)ave_ = [P]_cell(F)ave_ ÷ 0.033  [P]_mit(R)Mo_ = [P]_cell(R)Mo_ ÷ 0.10  For selected entries…  [P]_mit(Fsa)_ = [P]_cell(Rsa)_ ÷ 0.033  [P]_mit(Rsa)_ = [P]_cell(Rsa)_ ÷ 0.10 |
| 35 | Aco1 | YLR304C | 56807 (F Ho)  79401 (F Mo)  152031 (R Mo) | 2.246  3.139  2.69 (ave F)  6.011 (R) | 68.06  95.12  81.6 (ave F)  60.11 (R) |
| 36 | Aco2 | YJL200C | 15862 (F Ho)  20667 (F Mo)  4112 (R Mo) | 0.6271  0.8171  0.7221 (Ave F)  0.1626 (R) | 19.00  20.66  19.8 (ave F)  1.626 (R) |
| 37 | Aim32 | YML050W | 1176(F Ho)  208 (F Mo)  508 (R Mo) | 0.04650  0.0082  0.0274 (Ave F)  0.0200 (R) | 1.409  0.248  0.82 (ave F)  0.200 (R) |
| 38 | Bio2 | YGR286C | 4154 (F Ho)  1315 (F Mo)  988 (R Mo) | 0.1642  0.0520  0.108 (Ave F)  0.0391 (R) | 4.976  1.575  3.27 (ave F)  0.391 (R) |
| 39 | Bol1 | YAL044W-A | 4236 (F Ho)  5930 (F Mo)  13959 (R Mo) | 0.1675  0.2344  0.201 (Ave F)  0.5519 (R) | 5.0758  7.103  6.09 (ave F)  5.52 (R) |
| 40 | Bol3 | YAL046C | 2649 (F Ho)  1257 (F Mo)  1517 (R Mo) | 0.1047  0.0497  0.077 (Ave F)  0.0600 (R) | 3.173  1.506  2.34 (ave F)  0.600 (R) |
| 41 | Ccp1 | YKR066C | 8441 (F Ho)  8440 (F Mo)  18188 (R Mo) | 0.33380  0.3370  0.335 (Ave F)  0.7191 (R) | 10.12  10.21  10.16 (ave F)  7.191 (R) |
| 42 | Cob1 | Q0105 | 8283 (F Ho)  (NA) | 0.3275 (F) | 9.924 (F)  17.4 (Fsa)  22.9 (Rsa) |
| 43 | Coq7 | YOR125C | 2646 (F Ho)  991 (F Mo)  796 (R Mo) | 0.1046  0.0392  0.072 (Ave F)  0.0147 (R) | 3.170  1.188  2.18 (ave F)  0.147 (R) |
| 44 | Cox1 | Q0045 | 2382 (F Ho)  1200 (F Mo)  9596 (R Mo) | 0.09418 (F Ho)  0.0474 (F Mo)  0.0708 (Ave F)  0.3794 (R) | 2.854 (F Ho)  1.436 (F Mo)  2.145 (ave F)  3.794 (R)  7.93 (Fsa)  11.4 (Rsa) |
| 45 | Cox10 | YPL172C | 734 (F Ho) | 0.02902 (F) | 0.879 (F) |
| 46 | Cox15 | YER141W | 5872 (F Ho)  2241 (F Mo)  3080 (R Mo) | 0.2322  0.0886  0.160 (Ave F)  0.1218 (R) | 7.036  2.685  4.860 (ave F)  1.218 (R) |
| 47 | Cyb2 | YML054C | 6968 (F Ho)  5390 (F Mo)  48598 (R Mo) | 0.2755  0.2131  0.244 (Ave F)  1.921 (R) | 8.348  6.458  7.403 (ave F)  19.21 (R) |
| 48 | Cyc1 | YJR048W | 9225 (F Ho)  54146 (F Mo)  155053 (R Mo) | 0.3647  2.141  1.253 (Ave F)  6.1042 (R) | 11.05  64.88  37.97 (ave F)  61.042 (R) |
| 49 | Cyc7 | YEL039C | 3192 (F Ho)  3579 (F Mo)  3798 (R Mo) | 0.1262  0.1415  0.134 (Ave F)  0.1502 (R) | 3.824  4.288  4.056 (ave F)  1.502 (R) |
| 50 | Cyt1 | YOR065W | 6247 (F Ho)  10878 (F Mo)  34499 (R Mo) | 0.2470  0.4300  0.338 (Ave F)  1.364 (R) | 7.485  13.03  10.26 (ave F)  13.64 (R)  17.4 (Fsa)  22.9 (Rsa) |
| 51 | Exo5 | YBR163w | 1723 (F Ho) | 0.06812 (F) | 2.064 (F) |
| 52 | Fre5 | YOR384w | 2212 (F Ho) | 0.08746 (F) | 2.650 (F) |
| 54 | Grx5 | YPL059W | 7362 (F Ho)  12541 (F Mo)  17790 (R Mo) | 0.2911  0.4958  0.393 (Ave F)  0.7034 (R) | 8.821  15.024  11.92 (ave F)  7.034 (R) |
| 54 | Hem15 | YOR176W | 8709 (F Ho)  8593 (F Mo)  11302 (R Mo) | 0.3443  0.3397  0.342 (Ave F)  0.4468 (R) | 10.43  10.29  10.36 (ave F)  4.468 (R) |
| 55 | Ilv3 | YJR016C | 47064 (F Ho)  43051 (F Mo)  35443 (R Mo) | 1.861  1.702  1.781 (Ave F)  1.401 (R) | 56.39  51.57  53.98 (ave F)  14.01 (R) |
| 56 | Isa1 | YLL027W | 1542 (F Ho) | 0.0610 (F) | 1.848 (F) |
| 57 | Isa2 | YPR067W | 2620 (F Ho)  200 (F Mo)  333 (R Mo) | 0.1036  0.008  0.0558 (Ave F)  0.0132 (R) | 3.221  0.24  1.73 (ave F)  0.132 (R) |
| 58 | Isu1 | YPL135W | 7702 (F Ho)  852 (F Mo)  1679 (R Mo) | 0.3045  0.03369  0.169 (Ave F)  0.06638 (R) | 9.227  1.021  5.124 (ave F)  0.6638 (R) |
| 59 | Isu2 | YOR226C | 4518 (F Ho)  145 (F Mo)  57 (R Mo) | 0.1786  0.0057  0.0921 (Ave F)  0.0022 (R) | 5.41  0.173  2.79 (ave F)  0.022 (R) |
| 60 | Lip5 | YOR196C | 3870 (F Ho)  918 (F Mo)  875 (R Mo) | 0.1530  0.0363  0.0946 (Ave F)  0.0346 (R) | 4.636  1.100  2.87 (ave F)  0.346 (R) |
| 61 | Lys4 | YDR234W | 10156 (F Ho)  11833 (F Mo)  3063 (R Mo) | 0.4015  0.4678  0.435 (Ave F)  0.1211 (R) | 12.17  14.18  13.18 (ave F)  1.211 (R) |
| 62 | Mss51 | YLR203C | 6426 (F Ho)  2275 (F Mo)  3333 (R Mo) | 0.2541  0.0900  0.172 (Ave F)  0.1318 (R) | 7.700  2.727  5.213 (ave F)  1.318 (R) |
| 63 | Nfu1 | YKL040C | 7662 (F Ho)  10527 (F Mo)  12490 (R Mo) | 0.3029  0.4162  0.359 (Ave F)  0.4938 (R) | 9.179  12.612  10.90 (ave F)  4.938 (R) |
| 64 | Rip1 | YEL024W | 10319 (F Ho)  9128 (F Mo)  35070 (R Mo) | 0.4080  0.3609  0.384 (Ave F)  1.3866 (R) | 12.36  10.936  11.65 (ave F)  13.866 (R)  17.4 (Fsa)  22.9 (Rsa) |
| 65 | Sdh2 | YLL041C | 8535 (F Ho)  11429 (F Mo)  85568 (R Mo) | 0.3374  0.4519  0.395 (Ave F)  3.3832 (R) | 10.224  13.693  11.96 (ave F)  33.832 (R)  7.53 (Fsa)  14.9 (Rsa) |
| 66 | Sdh3 | YKL141W | 4719 (F Ho)  2665 (F Mo)  18383 (R Mo) | 0.1866  0.1054  0.146 (Ave F)  0.7268 (R) | 5.654  3.194  4.42 (ave F)  7.268 (R)  7.53 (Fsa)  14.9 (Rsa) |
| 67 | Sdh4 | YDR178W | 3115 (F Ho)  3377 (F Mo)  12815 (R Mo) | 0.1232  0.1335  0.1284 (Ave F)  0.5067 (R) | 3.733  4.045  3.89 (ave F)  5.067 (R)  7.53 (Fsa)  14.9 (Rsa) |
| 68 | Thi4 | YGR144W | 2611 (F Ho)  (NA) | 0.1032 (F) | 3.127 (F) |
| 69 | Thi5 | YFL058W | 1193 (F Ho)  (NA) | 0.04717 (F) | 1.264 (F) |
| 70 | Yah1 | YPL252C | 3599 (F Ho)  791 (F Mo)  551 (R Mo) | 0.1423  0.0313  0.087 (Ave F)  0.0218 (R) | 4.312  0.948  2.63 (ave F)  0.218 (R) |
| 71 | Yfh1 | YDL120W | 3366 (F Ho)  1321 (F Mo)  1471 (R Mo) | 0.1331  0.0522  0.0926 (Ave F)  0.0582 (R) | 4.033  1.582  2.808 (ave F)  0.582 (R) |
|  |  |  |  |  |  |
|  | **Nucleus** |  |  |  | [P]_nuc(F)_ = [P]_nuc(R)_ = [P]_cell_ ÷ 0.125 |
| 72 | Chl1 | YPL008W | 395 | 0.01562 | 0.1249 |
| 73 | Dna2 | YHR164C | 1836 | 0.07259 | 0.58073 |
| 74 | Hap1 | YLR256W | 1412 | 0.05583 | 0.4466 |
| 75 | Hap4 | YKL109W | 790 | 0.03123 | 0.2499 |
| 76 | Ntg2 | YOL043C | 90 | 0.00356 | 0.02847 |
| 77 | Pol1 | YNL102W | 2551 | 0.1009 | 0.8069 |
| 78 | Pol2 | YNL262W | 3195 | 0.1263 | 1.011 |
| 79 | Pol3 | YDL102W | 3200 | 0.1265 | 1.012 |
| 80 | Pri2 | YKL045W | 2413 | 0.09540 | 0.7632 |
| 81 | Rad3 | YER171W | 2892 | 0.1143 | 0.9147 |
| 82 | Rev3 | YPL167C | 1454 | 0.05749 | 0.4599 |
| 83 | Tpa1 | YER049W | 8155 | 0.3224 | 2.579 |
| 84 | Yap5 | YIR018W | 609 | 0.02408 | 0.1926 |
|  |  |  |  |  |  |
|  | **Endoplasmic Reticulum** |  |  |  | [P]_er(F)_ = [P]_er(R)_ = [P]_cell_ ÷ 0.015 |
| 85 | Cyb5 | YNL111C | 5939 | 0.2348 | 15.65 |
| 86 | Cyp51 | YHR007C | 16772 | 0.6631 | 44.21 |
| 87 | Dap1 | YPL170W | 5892 | 0.2330 | 15.53 |
| 88 | Erg3 | YLR056W | 7626 | 0.3015 | 20.10 |
| 89 | Erg5 | YMR015C | 8728 | 0.3451 | 23.01 |
| 90 | Erg25 | YGR060W | 24158 | 0.9552 | 63.68 |
| 91 | Fre8 | YLR047c | 770 | 0.03044 | 2.030 |
| 92 | Grx6 (golgi) | YDL010W | 2629 | 0.1039 | 6.930 |
| 93 | Hmx1 | YLR205C | 3068 | 0.1213 | 8.087 |
| 94 | Mpo1 | YGL010W | 2017 | 0.07975 | 5.316 |
| 95 | Ole1 | YGL055W | 9584 | 0.3789 | 25.26 |
| 96 | Scs7 | YMR272C | 4169 | 0.1648 | 10.99 |
| 97 | Sfh5 (unknown membrane) | YJL145W | 6822 | 0.2697 | 17.98 |
| 98 | Sur2 | YDR297W | 7012 | 0.2772 | 18.48 |
| 99 | Yno1 | YGL160w | 1189 | 0.04701 | 3.134 |
|  |  |  |  |  |  |
|  | **Vacuole** |  |  |  | [P]_vac(F)_ = [P]_vac(R)_ = [P]_cell_ ÷ 0.068 |
| 100 | Fre6 | YLL051C | 2071 | 0.08188 | 1.204 |

**Table S2: Fractional Volumes of yeast cellular compartments.** The term “cytosol” includes all other regions in the cell that are not specified by the indicated compartments.

| Compartment | Percentage of Cell volume [121]. | Percentage of Cell volume [122]. | Assumed fractional volumes | Assumed absolute volumes  (×10^-15^ L) |
| --- | --- | --- | --- | --- |
| Whole Cell | 100 | 100 | *f_cell_* = 1.000 | 42 |
| Cell Wall | 17 | 15.9 | *f_wall_* = 0.077 or 0.144 | 3.2-6.0 |
| Cytosol | 64 | --- | *f_cyt_* = 0.615 | 25.7 |
| Nucleus | 10.5 | 12.5 [124] | *f_nuc_* = 0.125 | 5.25 |
| Vacuoles | 5.8 | 7.8 | *f_vac_* = 0.068 | 2.8 |
| Mitochondria | 1.7 | 1.6 | *f_mit(F)_* = 0.033 or *f_mit(R)_* = 0.100 | 1.4 (F)  4.2(R) |
| ER | 0.7 | 2.2 | *f_er_* = 0.015 | 0.63 |

**Figure S1. Visual explanation of F, R, Fsa, and Rsa assumptions.** Consider a protein P in which 1 copy is present per cell when the cell is grown under fermenting conditions. For convenience, consider that the cellular concentration is 1 µM. If mitochondria represent 0.033 of total cell volume, the local concentration of P ([P]_mit(F)_) would be 1/0.033. Under respiring conditions, the R scenario assumes that 3-times more mitochondria are present per cell, but that the cellular expression level of each mitochondrial protein matches the increased fractional volume of mitochondria. Thus, [P]_cell(R)_ = 3 and [P]_mit(R)_ = 3/0.10 = [P]_mit(F)_. Now consider that P is an iron-containing subunit of a protein complex of known composition. In the Fsa and Rsa (sa = subunit averaged) calculations, the presumed concentration of P was determined by averaging the concentrations of all subunits in the complex. This was done to improve the accuracy of [P] for both fermenting (illustrated) and respiring (not illustrated) conditions.


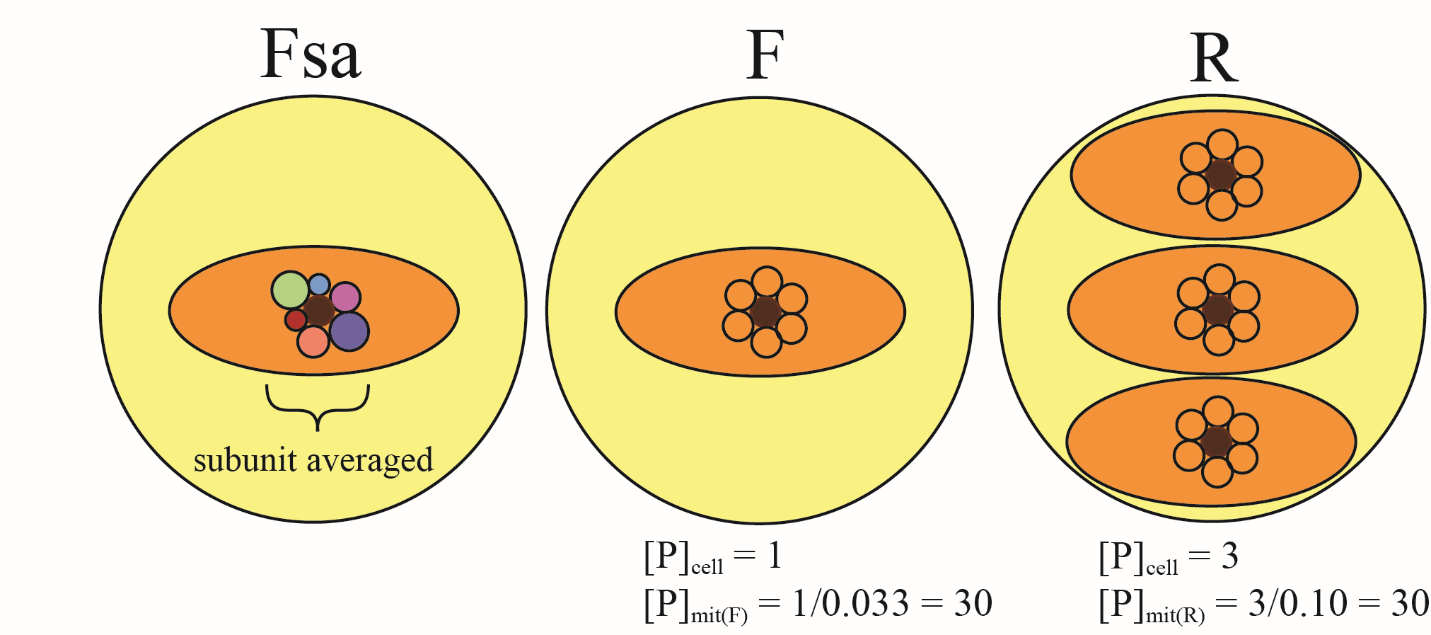


**Table S3: Iron centers contained in each iron protein and their contribution to the ironome.** Eight types of centers were considered, including [Fe_4_S_4_], [Fe_3_S_4_], [Fe_2_S_2_], high-spin hemes, low-spin hemes, Fe^II^(cys)_4_, Fe^II^(O/N), and [Fe-O-Fe] centers. Structural details beyond these divisions were not considered. Assumptions pertaining to the occupancy of the center in the protein and the quaternary structure of the proteins and associated protein complexes are indicated. From this, the contribution of iron from each center (in µM) per monomer protein was calculated. Local protein concentrations [P]_i_ are for fermenting and respiring cells assuming Fsa and Rsa scenarios.

Ilv3 was previously presumed to contain an [Fe_4_S_4_] cluster but it now appears to contain an [Fe_2_S_2_] cluster [3]. The enzyme is homologous to those from *Escherichia coli* and *Mycobacterium tuberculosis*. A recently obtained structure of the enzyme from *M. tuberculosis*, purified and crystalized anaerobically, reveals a novel [Fe_2_S_2_] cluster in which one iron has an open coordinate site to which water, OH^-^, or substrate might bind [147]. The yeast and *E. coli* enzymes probably also contains an [Fe_2_S_2_] cluster.

| **Cytosol** | **Assumptions** | **Iron center(s) per monomer** | **Manipulation of [P]_cyt(F)_** | **[Fe_4_S_4_]** | **[Fe_3_S_4_]** | **[Fe_2_S_2_]** | **H.S. Heme** | **L.S. Heme** | **Fe^II^_(cys)4_** | **Fe^II^_O/N_** | **Fe-O-Fe** | **Cytosol [Iron]** | **Fraction** |
| --- | --- | --- | --- | --- | --- | --- | --- | --- | --- | --- | --- | --- | --- |
| Aft1 | 100% has bridging cluster complexed with Aft1, Aft2, Bol2, Grx3, or Grx4 | 0.5 [Fe_2_S_2_] | [P]_cyt_×2÷2 |  |  | 0.1651 |  |  |  |  |  | 0.1651 | 0.00269 |
| Aft2 | 100% has bridging cluster complexed with Bol2, Grx3, or Grx4 (evenly distributed) | 0.5 [Fe_2_S_2_] | [P]_cyt_×2÷2 |  |  | 0.01684 |  |  |  |  |  | 0.01684 | 0.00027 |
| Apd1 | 100% bound monomer | 1 [Fe_2_S_2_] | [P]_cyt_×2 |  |  | 0.6079 |  |  |  |  |  | 0.6079 | 0.00989 |
| Bna1 | 100% bound | 1Fe^II^_(O/N)_ | [P]_cyt_ |  |  |  |  |  |  | 0.4861 |  | 0.4861 | 0.00791 |
| Bol2 | 100% bridging cluster complexed with Aft1, Aft2, Grx3, Grx4 | 0.5 [Fe_2_S_2_] | [P]_cyt_×2÷2 |  |  | 0.2097 |  |  |  |  |  | 0.2097 | 0.00341 |
| Cfd1 | 100% bridging cluster between two Cfd1 monomers | 0.5 [Fe_4_S_4_] | [P]_cyt_×4÷2 | 0.1948 |  |  |  |  |  |  |  | 0.1948 | 0.00317 |
| Cta1 | 100% bound | 1 heme b, HS | [P]_cyt_ |  |  |  | 0.07985 |  |  |  |  | 0.07985 | 0.00130 |
| Ctt1 | 100% bound | 1 heme b, HS | [P]_cyt_ |  |  |  | 0.3718 |  |  |  |  | 0.3718 | 0.00605 |
| Dbr1 | 100% bound | 1 Fe^II^_(O/N)_ |  |  |  |  |  |  |  | 0.09933 |  | 0.09933 | 0.00162 |
| Dph1 | 100% bound cluster bridged | 0.5 [Fe_4_S_4_] | [P]_cyt_×4÷2 | 0.3726 |  |  |  |  |  |  |  | 0.3726 | 0.00606 |
| Dph2 | 100% bound cluster bridged | 0.5 [Fe_4_S_4_] | [P]_cyt_×4÷2 | 0.4523 |  |  |  |  |  |  |  | 0.4523 | 0.00736 |
| Dph3 | 100% bound | 1Fe^II^_(cys)4_ | [P]_cyt_ |  |  |  |  |  | 0.5676 |  |  | 0.5676 | 0.00924 |
| Dph4 | 100% bound | 1Fe^II^_(cys)4_ | [P]_cyt_ |  |  |  |  |  | 0.1270 |  |  | 0.126 | 0.00207 |
| Dre2 | 100% bound.  Controversial metal content. | 1.5 [Fe_2_S_2_]  0.5 [Fe_4_S_4_] | [P]_cyt_×4÷2  and  [P]_cyt_×2×1.5 | 0.6206 |  | 0.9310 |  |  |  |  |  | 1.552 | 0.0252 |
| Elp3 | 100% bound | 1[Fe_4_S_4_] | [P]_cyt_×4 | 1.2035 |  |  |  |  |  |  |  | 1.2035 | 0.0196 |
| Fre1 | 100% bound | 2 LS hemes | [P]_cyt_×2 |  |  |  |  | 0.2757 |  |  |  | 0.2757 | 0.00449 |
| Fre2 | 50% bound;  Uncertain | 2 LS hemes | [P]_cyt_×2÷2 |  |  |  |  | 0.08210 |  |  |  | 0.08210 | 0.00134 |
| Fre3 | 50% bound;  Uncertain | 2 LS heme | [P]_cyt_×2÷2 |  |  |  |  | 0.08210 |  |  |  | 0.08210 | 0.00134 |
| Fre4 | 50% bound;  Uncertain | 2 LS heme | [P]_cyt_×2÷2 |  |  |  |  | 0.07335 |  |  |  | 0.07335 | 0.00119 |
| Fre7 | 50% bound;  Uncertain | 2 LS heme | [P]_cyt_×2÷2 |  |  |  |  | 0.00771 |  |  |  | 0.00771 | 0.00013 |
| Glt1 | 100% bound | 2[Fe_4_S_4_] and 1 [Fe_3_S_4_] per monomer | [P]_cyt_×4×2  and  [P]_cyt_×3 | 7.282 | 2.730 |  |  |  |  |  |  | 10.01 | 0.1630 |
| Grx3 | 100% bound cluster bridged | 0.5 [Fe_2_S_2_] | [P]_cyt_×2÷2 |  |  | 0.5142 |  |  |  |  |  | 0.5142 | 0.00837 |
| Grx4 | 100% bound cluster bridged | 0.5 [Fe_2_S_2_] | [P]_cyt_×2÷2 |  |  | 0.2738 |  |  |  |  |  | 0.2738 | 0.00446 |
| Leu1 | 100% bound | 1[Fe_4_S_4_] | [P]_cyt_×4 | 17.58 |  |  |  |  |  |  |  | 17.58 | 0.286 |
| Lia1 | 100% bound | 1 Fe^II^_(O/N)_ | [P]_cyt_ |  |  |  |  |  |  | 1.362 |  | 1.362 | 0.0222 |
| Met5 | 100% bound | 1 HS heme and 1 [Fe_4_S_4_] | [P]_cyt_×4 | 2.183 |  |  | 0.5458 |  |  |  |  | 2.729 | 0.0444 |
| Nar1 | 100% bound | 2[Fe_4_S_4_] | [P]_cyt_×4×2 | 0.7298 |  |  |  |  |  |  |  | 0.7298 | 0.0119 |
| Nbp35 | 100% 1 cluster bridging and 1 cluster not bridging | 1.5[Fe_4_S_4_] | [P]_cyt_×4×1.5 | 3.220 |  |  |  |  |  |  |  | 3.220 | 0.0524 |
| Ncs6 | 100% bound | 1 [Fe_3_S_4_] | [P]_cyt_×3 |  | 0.5105 |  |  |  |  |  |  | 0.5105 | 0.00831 |
| Rli1 | 100% bound | 2 [Fe_4_S_4_] | [P]_cyt_×4×2 | 8.318 |  |  |  |  |  |  |  | 8.318 | 0.135 |
| Rnr2 | 100% bound | 1[Fe-O-Fe] | [P]_cyt_×2 |  |  |  |  |  |  |  | 4.039 | 4.039 | 0.0657 |
| Tdh3 | 0.0169% bound | 0.000169 Heme b | [P]_cyt_×0.000169 |  |  |  |  | 0.01099 |  |  |  | 0.01099 | 0.00018 |
| Tyw1 | 100% bound | 2[Fe_4_S_4_] | [P]_cyt_×4×2 | 2.255 |  |  |  |  |  |  |  | 2.255 | 0.0367 |
| Yhb1 | 100% bound | 1heme b | [P]_cyt_×1 |  |  |  | 2.857 |  |  |  |  | 2.857 | 0.0465 |
| **Total** |  | **…** | **…** | **44.41** | **3.241** | **2.719** | **3.854** | **0.5319** | **0.6946** | **1.947** | **4.039** | **61.44** | 1.000 |
| **Fraction** |  |  |  | **0.723** | **0.053** | **0.044** | **0.063** | **0.009** | **0.011** | **0.032** | **0.066** |  |  |
|  |  |  |  |  |  |  |  |  |  |  |  |  |  |
| **Nucleus** | **Assumptions** | **Iron center(s) per monomer** | **Manipulation of [P]_nuc(F)_** | **[Fe_4_S_4_]** | **[Fe_3_S_4_]** | **[Fe_2_S_2_]** | **H.S. Heme** | **L.S. Heme** | **Fe^II^_S_** | **Fe^II^_O/N_** | **Fe-O-Fe** | **Nucleus [Iron]** |  |
| Chl1 | 100% bound | 1 [Fe_4_S_4_] | [P]_nuc_×4 | 0.50 |  |  |  |  |  |  |  | 0.50 | 0.019 |
| Dna2 | 100% bound | 1 [Fe_4_S_4_] | [P]_nuc_×4 | 2.32 |  |  |  |  |  |  |  | 2.32 | 0.087 |
| Hap1 | 50% bound; LS heme | 0.5 heme b | [P]_nuc_÷2 |  |  |  |  | 0.22 |  |  |  | 0.22 | 0.008 |
| Hap4 | 50% bound; LS heme | 0.5 heme b | [P]_nuc_÷2 |  |  |  |  | 0.12 |  |  |  | 0.12 | 0.005 |
| Ntg2 | 100% bound | 1 [Fe_4_S_4_] | [P]_nuc_×4 | 0.11 |  |  |  |  |  |  |  | 0.11 | 0.004 |
| Pol1 | 100% bound | 1 [Fe_4_S_4_] | [P]_nuc_×4 | 3.23 |  |  |  |  |  |  |  | 3.23 | 0.121 |
| Pol2 | 100% bound | 1 [Fe_4_S_4_] | [P]_nuc_×4 | 4.04 |  |  |  |  |  |  |  | 4.04 | 0.151 |
| Pol3 | 100% bound | 1 [Fe_4_S_4_] | [P]_nuc_×4 | 4.05 |  |  |  |  |  |  |  | 4.05 | 0.152 |
| Pri2 | 100% bound | 1 [Fe_4_S_4_] | [P]_nuc_×4 | 3.05 |  |  |  |  |  |  |  | 3.05 | 0.114 |
| Rad3 | 100% bound | 1 [Fe_4_S_4_] | [P]_nuc_×4 | 3.66 |  |  |  |  |  |  |  | 3.66 | 0.137 |
| Rev3 | 100% bound | 1 [Fe_4_S_4_] | [P]_nuc_×4 | 1.84 |  |  |  |  |  |  |  | 1.84 | 0.069 |
| Tpa1 | 100% bound | 1 Fe^II^_O/N_ | [P]_nuc_ |  |  |  |  |  |  | 2.58 |  | 2.58 | 0.097 |
| Yap5 | 100% bound | 1.5[Fe_2_S_2_]  and  0.5[Fe_4_S_4_] | [P]_nuc_×4÷2  and  [P]_nuc_×2×1.5 | 0.38 |  | 0.58 |  |  |  |  |  | 0.96 | 0.036 |
| **Totals** |  |  |  | **23.19** | **0** | **0.58** | **0** | **0.348** | **0** | **2.58** | **0** | **26.70** |  |
| **Fraction** |  |  |  | **0.868** | **0** | **0.022** | **0** | **0.013** | **0** | **0.097** | **0** | 1.000 |  |
|  |  |  |  |  |  |  |  |  |  |  |  |  |  |
| **Endoplasmic Reticulum** | **Assumptions** | **Iron center(s) per monomer** | **Manipulation of [P]_ER(F)_** | **[Fe_4_S_4_]** | **[Fe_3_S_4_]** | **[Fe_2_S_2_]** | **H.S. Heme** | **L.S. Heme** | **Fe^II^_S_** | **Fe^II^_O/N_** | **Fe-O-Fe** | **[Iron]_ER_** |  |
| Cyb5 | 100% bound | 1 heme b | [P]_er_ |  |  |  |  | 15.65 |  |  |  | 15.65 | 0.034 |
| Cyp51 | 100% bound | 1 heme b (cys) | [P]_er_ |  |  |  |  | 44.21 |  |  |  | 44.21 | 0.096 |
| Dap1 | 100% bound | 1 heme b | [P]_er_ |  |  |  |  | 15.53 |  |  |  | 15.53 | 0.034 |
| Erg3 | 100% bound | 1[Fe-O-Fe] | [P]_er_×2 |  |  |  |  |  |  |  | 40.20 | 40.20 | 0.088 |
| Erg5 | 100% bound | 1 heme b (cys) | [P]_er_ |  |  |  |  | 23.01 |  |  |  | 23.01 | 0.050 |
| Erg25 | 100% bound | 1[Fe-O-Fe] | [P]_er_×2 |  |  |  |  |  |  |  | 127.4 | 127.4 | 0.278 |
| Fre8 | 100% bound | 1 heme b | [P]_er_ |  |  |  |  | 2.03 |  |  |  | 2.03 | 0.004 |
| Grx6(golgi) | 5% bound | 0.5 [Fe_2_S_2_] | [P]_er_×2÷2 |  |  | 6.93 |  |  |  |  |  | 6.93 | 0.015 |
| Hmx1 | 100% bound | 1 heme b | [P]_er_ |  |  |  |  | 8.09 |  |  |  | 8.09 | 0.018 |
| Mpo1 | 100% bound | 1 Fe^II^_O/N_ | [P]_er_ |  |  |  |  |  |  | 5.32 |  | 5.32 | 0.012 |
| Ole1 | 100% bound | 1 [Fe-O-Fe]; 1 heme b | [P]_er_×3 |  |  |  |  | 25.26 |  |  | 50.52 | 75.79 | 0.165 |
| Scs7 | 100% bound | 1 [Fe-O-Fe]; 1 heme b | [P]_er_×3 |  |  |  |  | 10.99 |  |  | 21.98 | 32.97 | 0.072 |
| Sfh5 | 100% bound | 1 HS heme b (tyr) | [P]_er_ |  |  |  | 17.98 |  |  |  |  | 17.98 | 0.039 |
| Sur2 | 100% bound | 1 [Fe-O-Fe] | [P]_er_×2 |  |  |  |  |  |  |  | 36.96 | 36.96 | 0.081 |
| Yno1 | 100% bound | 2 heme b  Low spin | [P]_er_×2 |  |  |  |  | 6.27 |  |  |  | 6.27 | 0.014 |
| **Totals** |  |  |  | **0** | **0** | **6.93** | **17.98** | **151.0** | **0** | **5.32** | **277.0** | **458.3** |  |
| **Fraction** |  |  |  |  |  | **0.015** | **0.039** | **0.330** | **0** | **0.012** | **0.604** | **1.000** |  |
|  |  |  |  |  |  |  |  |  |  |  |  |  |  |
| **Vacuoles** | **Assumptions** | **Iron center(s) per monomer** | **Manipulation of [P]_vac(F)_** | **[Fe_4_S_4_]** | **[Fe_3_S_4_]** | **[Fe_2_S_2_]** | **H.S. Heme** | **L.S. Heme** | **Fe^II^_S_** | **Fe^II^_O/N_** | **Fe-O-Fe** | **[Iron]_ER_** |  |
| Fre6 |  | 1 heme b | [P]_vac_ |  |  |  |  | 1.20 |  |  |  | 1.20 | 1.000 |
| **Totals** |  |  |  | **0** | **0** | **0** | **0** | **1.20** | **0** | **0** | **0** | **1.20** |  |
| **Fraction** |  |  |  | **0** | **0** | **0** | **0** | **1.00** | **0** | **0** | **0** |  | **1.000** |
|  |  |  |  |  |  |  |  |  |  |  |  |  |  |
|  |  |  |  |  |  |  |  |  |  |  |  |  |  |
| **Mitochondria (F)** |  | **Iron center(s)** | **Manipulation of [P]_mit(F)_** | **[Fe_4_S_4_]** | **[Fe_3_S_4_]** | **[Fe_2_S_2_]** | **H.S. Heme** | **L.S. Heme** | **Fe^II^_S_** | **Fe^II^_O/N_** | **Fe-O-Fe** | **Mito. [Iron]** |  |
| Aco1 | 100% metallated | 1[Fe_4_S_4_] | [P]_mit(F)_×4 | 326.4 |  |  |  |  |  |  |  | 326.4 | 0.320 |
| Aco2 | 100% metallated | 1[Fe_4_S_4_] | [P]_mit(F)_×4 | 79.2 |  |  |  |  |  |  |  | 79.2 | 0.078 |
| Aim32 | 100% bound  monomer | 1 [Fe_2_S_2_] | [P]_mit(F)_×2 |  |  | 1.64 |  |  |  |  |  | 1.64 | 0.002 |
| Bio2 | 100% bound | 1[Fe_4_S_4_]  and  1[Fe_2_S_2_] | [P]_mit(F)_×4  +  [P]_mit(F)_×2 | 13.08 |  | 6.54 |  |  |  |  |  | 19.62 | 0.019 |
| Bol1 | 100% bound cluster bridging | 0.5 [Fe_2_S_2_] | [P]_mit(F)_×2÷2 |  |  | 6.09 |  |  |  |  |  | 6.09 | 0.006 |
| Bol3 | 100% bound cluster bridging | 0.5 [Fe_2_S_2_] | [P]_mit(F)_×2÷2 |  |  | 2.34 |  |  |  |  |  | 2.34 | 0.002 |
| Ccp1 | 100% bound | 2 heme b | [P]_mit(F)_×2 |  |  |  |  | 20.32 |  |  |  | 20.32 | 0.020 |
| Cob1 | 100% bound | 2 heme b | [P]_mit(F)_×2 |  |  |  |  | 19.85 |  |  |  | 19.85 | 0.019 |
| Coq7 | 100% bound | 1[Fe-O-Fe] | [P]_mit(F)_×2 |  |  |  |  |  |  |  | 4.36 | 4.36 | 0.004 |
| Cox1 | 100% bound | 1 LS heme a  and  1 HS heme a3 | [P]_mit(F)_  and  [P]_mit(F)_ |  |  |  | 2.14 | 2.14 |  |  |  | 4.28 | 0.004 |
| Cox10 | 100% bound  (uncertain) | 1 HS? heme o | [P]_mit(F)_ |  |  |  | 0.88 |  |  |  |  | 0.88 | 0.001 |
| Cox15 | 100% bound  (uncertain) | 1 HS? heme b and  1 HS? heme a | [P]_mit(F)_  and  [P]_mit(F)_ |  |  |  | 9.72 |  |  |  |  | 9.72 | 0.010 |
| Cyb2 | 100% bound | 1 LS heme b | [P]_mit(F)_ |  |  |  |  | 7.40 |  |  |  | 7.40 | 0.007 |
| Cyc1 | 100% bound | 1 LS heme c | [P]_mit(F)_ |  |  |  |  | 37.97 |  |  |  | 37.97 | 0.037 |
| Cyc7 | 100% bound | 1 LS heme c | [P]_mit(F)_ |  |  |  |  | 4.06 |  |  |  | 4.06 | 0.004 |
| Cyt1 | 100% bound | 1 heme c | [P]_mit(F)_ |  |  |  |  | 10.26 |  |  |  | 10.26 | 0.010 |
| Exo5 | 100% bound | 1 [Fe_4_S_4_] | [P]_mit(F)_×4 | 8.26 |  |  |  |  |  |  |  | 8.26 | 0.008 |
| Fre5 | 50% bound  (uncertain) | 2 LS heme b | [P]_mit(F)_×2÷2 |  |  |  |  | 2.65 |  |  |  | 2.65 | 0.003 |
| Grx5 | 100% bound cluster bridging | 0.5[Fe_4_S_4_]  and  1.5[Fe_2_S_2_] | [P]_mit(F)_×4÷2  and  [P]_mit(F)_×2×1.5 | 23.84 |  | 35.76 |  |  |  |  |  | 59.60 | 0.058 |
| Hem15 | 100% bound | 1 Fe^II^_O/N_ | [P]_mit(F)_ |  |  |  |  |  |  | 10.36 |  | 10.36 | 0.010 |
| Ilv3 | 100% bound | 1 [Fe_2_S_2_] | [P]_mit(F)_×2 |  |  | 107.96 |  |  |  |  |  | 107.96 | 0.106 |
| Isa1 | 100% bound | 0.5 [Fe_4_S_4_]  and  0.5 [Fe_2_S_2_] | [P]_mit(F)_×4÷2  and  [P]_mit(F)_×2÷2 | 3.70 |  | 1.85 |  |  |  |  |  | 5.55 | 0.005 |
| Isa2 | 100% bound | 0.5 [Fe_4_S_4_] + and  0.5 [Fe_2_S_2_] | [P]_mit(F)_×4÷2  and  [P]_mit(F)_×2÷2 | 3.46 |  | 1.73 |  |  |  |  |  | 5.19 | 0.005 |
| Isu1 | 100% bound | 1 [Fe_2_S_2_] | [P]_mit(F)_×2 |  |  | 10.50 |  |  |  |  |  | 10.50 | 0.010 |
| Isu2 | 100% bound | 1 [Fe_2_S_2_] | [P]_mit(F)_×2 |  |  | 5.58 |  |  |  |  |  | 5.58 | 0.005 |
| Lip5 | 100% bound | 2 [Fe_4_S_4_] | [P]_mit(F)_×4×2 | 22.96 |  |  |  |  |  |  |  | 22.96 | 0.023 |
| Lys4 | 100% bound | 1 [Fe_4_S_4_] | [P]_mit(F)_×4 | 52.72 |  |  |  |  |  |  |  | 52.72 | 0.052 |
| Mss51 | 100% bound  (uncertain) | 1 HS? heme b | [P]_mit(F)_ |  |  |  | 5.21 |  |  |  |  | 5.21 | 0.005 |
| Nfu1 | 100% bound | 0.5 [Fe_4_S_4_] | [P]_mit(F)_×4÷2 | 21.80 |  |  |  |  |  |  |  | 21.80 | 0.021 |
| Rip1 | 100% bound | 1 [Fe_2_S_2_] (2 His ligands) | [P]_mit(F)_×2 |  |  | 23.30 |  |  |  |  |  | 23.30 | 0.023 |
| Sdh2 | 100% bound | 1 [Fe_4_S_4_]  and  1[Fe_3_S_4_]  and  1[Fe_2_S_2_] | [P]_mit(F)_×4  and  [P]_mit(F)_×3  and  [P]_mit(F)_×2 | 47.84 | 35.88 | 23.92 |  |  |  |  |  | 107.64 | 0.106 |
| Sdh3 | 100% bound bridging heme with Sdh4 | 0.5 heme b | [P]_mit(F)_÷2 |  |  |  |  | 2.21 |  |  |  | 2.21 | 0.002 |
| Sdh4 | 100% bound bridging heme with Sdh3 | 0.5 heme b | [P]_mit(F)_÷2 |  |  |  |  | 1.94 |  |  |  | 1.94 | 0.002 |
| Thi4 | 100% bound | Fe^II^_(N/O)_ | [P]_mit(F)_ |  |  |  |  |  |  | 3.13 |  | 3.13 | 0.003 |
| Thi5 | 100% bound | Fe^II^_(N/O)_ | [P]_mit(F)_ |  |  |  |  |  |  | 1.26 |  | 1.26 | 0.001 |
| Yah1 | 100% bound | 1 [Fe_2_S_2_] | [P]_mit(F)_×2 |  |  | 5.26 |  |  |  |  |  | 5.26 | 0.005 |
| Yfh1 | 50% bound  (uncertain) | 1 Fe^II^_O/N_ | [P]_mit(F)_÷2 |  |  |  |  |  |  | 1.40 |  | 1.40 | 0.001 |
| **Totals** |  |  |  | **603.26** | **35.88** | **232.47** | **17.95** | **108.8** | 0.000 | **16.15** | **4.36** | 1018.87 | **1.000** |
| **Fraction** |  |  |  | **0.592** | **0.035** | **0.228** | **0.018** | **0.107** | **0** | **0.016** | **0.004** | 1.000 |  |
|  |  |  |  |  |  |  |  |  |  |  |  |  |  |
| **Mitochondria (R)** |  | **Iron center(s)** | **Manipulation of [P]_mit(R)_** | **[Fe_4_S_4_]** | **[Fe_3_S_4_]** | **[Fe_2_S_2_]** | **H.S. Heme** | **L.S. Heme** | **Fe^II^_S_** | **Fe^II^_O/N_** | **Fe-O-Fe** | **Mito. [Iron]** |  |
| Aco1 | 100% metallated | 1[Fe_4_S_4_] | [P]_mit(R)_×4 | 240.44 |  |  |  |  |  |  |  | 240.44 | 0.285 |
| Aco2 | 100% metallated | 1[Fe_4_S_4_] | [P]_mit(R)_×4 | 6.50 |  |  |  |  |  |  |  | 6.50 | 0.008 |
| Aim32 | 100% bound  monomer | 1 [Fe_2_S_2_] | [P]_mit(R)_×2 |  |  | 0.40 |  |  |  |  |  | 0.40 | 0.000 |
| Bio2 | 100% bound | 1[Fe_4_S_4_]  and  1[Fe_2_S_2_] | [P]_mit(R)_×4  +  [P]_mit(R)_×2 | 1.56 |  | 0.78 |  |  |  |  |  | 2.34 | 0.003 |
| Bol1 | 100% bound cluster bridging | 0.5 [Fe_2_S_2_] | [P]_mit(R)_×2÷2 |  |  | 5.52 |  |  |  |  |  | 5.52 | 0.007 |
| Bol3 | 100% bound cluster bridging | 0.5 [Fe_2_S_2_] | [P]_mit(R)_×2÷2 |  |  | 0.60 |  |  |  |  |  | 0.60 | 0.001 |
| Ccp1 | 100% bound | 2 heme b | [P]_mit(R)_×2 |  |  |  |  | 14.38 |  |  |  | 14.38 | 0.017 |
| Cob1 | 100% bound | 2 heme b | [P]_mit(F)_×2 |  |  |  |  | 19.85 |  |  |  | 19.85 | 0.023 |
| Coq7 | 100% bound | 1[Fe-O-Fe] | [P]_mit(R)_×2 |  |  |  |  |  |  |  | 0.29 | 0.29 | 0.000 |
| Cox1 | 100% bound | 1 LS heme a  and  1 HS heme a3 | [P]_mit(R)_  and  [P]_mit(R)_ |  |  |  | 3.79 | 3.79 |  |  |  | 7.59 | 0.009 |
| Cox10 | 100% bound  (uncertain) | 1 HS? heme o | [P]_mit(F)_ |  |  |  | 0.88 |  |  |  |  | 0.88 | 0.001 |
| Cox15 | 100% bound  (uncertain) | 1 HS? heme b and  1 HS? heme a | [P]_mit(R)_  and  [P]_mit(R)_ |  |  |  | 2.44 |  |  |  |  | 2.44 | 0.003 |
| Cyb2 | 100% bound | 1 LS heme b | [P]_mit(R)_ |  |  |  |  | 19.21 |  |  |  | 19.21 | 0.023 |
| Cyc1 | 100% bound | 1 LS heme c | [P]_mit(R)_ |  |  |  |  | 61.04 |  |  |  | 61.04 | 0.072 |
| Cyc7 | 100% bound | 1 LS heme c | [P]_mit(R)_ |  |  |  |  | 1.50 |  |  |  | 1.50 | 0.002 |
| Cyt1 | 100% bound | 1 heme c | [P]_mit(R)_ |  |  |  |  | 13.64 |  |  |  | 13.64 | 0.016 |
| Exo5 | 100% bound | 1 [Fe_4_S_4_] | [P]_mit(F)_×4 | 8.26 |  |  |  |  |  |  |  | 8.26 | 0.010 |
| Fre5 | 50% bound  (uncertain) | 2 LS heme b | [P]_mit(F)_×2÷2 |  |  |  |  | 2.65 |  |  |  | 2.65 | 0.003 |
| Grx5 | 100% bound cluster bridging | 0.5[Fe_4_S_4_]  and  1.5[Fe_2_S_2_] | [P]_mit(R)_×4÷2  and  [P]_mit(R)_×2×1.5 | 14.07 |  | 21.10 |  |  |  |  |  | 35.17 | 0.042 |
| Hem15 | 100% bound | 1 Fe^II^_O/N_ | [P]_mit(R)_ |  |  |  |  |  |  | 4.47 |  | 4.47 | 0.005 |
| Ilv3 | 100% bound | 1 [Fe_2_S_2_] | [P]_mit(R)_×2 |  |  | 28.02 |  |  |  |  |  | 28.02 | 0.033 |
| Isa1 | 100% bound | 0.5 [Fe_4_S_4_]  and  0.5 [Fe_2_S_2_] | [P]_mit(F)_×4÷2  and  [P]_mit(F)_×2÷2 | 3.70 |  | 1.85 |  |  |  |  |  | 5.55 | 0.007 |
| Isa2 | 100% bound | 0.5 [Fe_4_S_4_] + and  0.5 [Fe_2_S_2_] | [P]_mit(R)_×4÷2  and  [P]_mit(R)_×2÷2 | 0.26 |  | 0.13 |  |  |  |  |  | 0.39 | 0.000 |
| Isu1 | 100% bound | 1 [Fe_2_S_2_] | [P]_mit(R)_×2 |  |  | 1.33 |  |  |  |  |  | 1.33 | 0.002 |
| Isu2 | 100% bound | 1 [Fe_2_S_2_] | [P]_mit(R)_×2 |  |  | 0.04 |  |  |  |  |  | 0.04 | 0.000 |
| Lip5 | 100% bound | 2 [Fe_4_S_4_] | [P]_mit(R)_×4×2 | 2.77 |  |  |  |  |  |  |  | 2.77 | 0.003 |
| Lys4 | 100% bound | 1 [Fe_4_S_4_] | [P]_mit(R)_×4 | 4.84 |  |  |  |  |  |  |  | 4.84 | 0.006 |
| Mss51 | 100% bound  (uncertain) | 1 HS? heme b | [P]_mit(R)_ |  |  |  | 1.32 |  |  |  |  | 1.32 | 0.002 |
| Nfu1 | 100% bound | 0.5 [Fe_4_S_4_] | [P]_mit(R)_×4÷2 | 9.88 |  |  |  |  |  |  |  | 9.88 | 0.012 |
| Rip1 | 100% bound | 1 [Fe_2_S_2_] (2 His ligands) | [P]_mit(R)_×2 |  |  | 27.73 |  |  |  |  |  | 27.73 | 0.033 |
| Sdh2 | 100% bound | 1 [Fe_4_S_4_]  and  1[Fe_3_S_4_]  and  1[Fe_2_S_2_] | [P]_mit(R)_×4  and  [P]_mit(R)_×3  and  [P]_mit(R)_×2 | 135.33 | 101.50 | 67.66 |  |  |  |  |  | 304.49 | 0.360 |
| Sdh3 | 100% bound bridging heme with Sdh4 | 0.5 heme b | [P]_mit(R)_÷2 |  |  |  |  | 3.63 |  |  |  | 3.63 | 0.004 |
| Sdh4 | 100% bound bridging heme with Sdh3 | 0.5 heme b | [P]_mit(R)_÷2 |  |  |  |  | 2.53 |  |  |  | 2.53 | 0.003 |
| Thi4 | 100% bound | Fe^II^_(N/O)_ | [P]_mit(F)_ |  |  |  |  |  |  | 3.13 |  | 3.13 | 0.004 |
| Thi5 | 100% bound | Fe^II^_(N/O)_ | [P]_mit(F)_ |  |  |  |  |  |  | 1.26 |  | 1.26 | 0.001 |
| Yah1 | 100% bound | 1 [Fe_2_S_2_] | [P]_mit(R)_×2 |  |  | 0.44 |  |  |  |  |  | 0.44 | 0.001 |
| Yfh1 | 50% bound  (uncertain) | 1 Fe^II^_O/N_ | [P]_mit(R)_÷2 |  |  |  |  |  |  | 0.29 |  | 0.29 | 0.000 |
| **Totals** |  |  |  | **427.61** | **101.50** | **155.60** | **8.43** | **142.22** | 0.000 | **9.15** | **0.29** | 844.81 | 1.000 |
| **Fraction** |  |  |  | **0.506** | **0.120** | **0.184** | **0.010** | **0.168** | **0** | **0.016** | **0.000** | 1.000 |  |
|  |  |  |  |  |  |  |  |  |  |  |  |  |  |
| **Mitochondria (Fsa)** |  | **Iron center(s)** | **Manipulation of [P]_mit(F)_** | **[Fe_4_S_4_]** | **[Fe_3_S_4_]** | **[Fe_2_S_2_]** | **H.S. Heme** | **L.S. Heme** | **Fe^II^_S_** | **Fe^II^_O/N_** | **Fe-O-Fe** | **Mito. [Iron]** |  |
| Aco1 | 100% metallated | 1[Fe_4_S_4_] | [P]_mit(F)_×4 | 326.4 |  |  |  |  |  |  |  | 326.4 |  |
| Aco2 | 100% metallated | 1[Fe_4_S_4_] | [P]_mit(F)_×4 | 79.2 |  |  |  |  |  |  |  | 79.2 |  |
| Aim32 | 100% bound  monomer | 1 [Fe_2_S_2_] | [P]_mit(F)_×2 |  |  | 1.64 |  |  |  |  |  | 1.64 |  |
| Bio2 | 100% bound | 1[Fe_4_S_4_]  and  1[Fe_2_S_2_] | [P]_mit(F)_×4  +  [P]_mit(F)_×2 | 13.08 |  | 6.54 |  |  |  |  |  | 19.62 |  |
| Bol1 | 100% bound cluster bridging | 0.5 [Fe_2_S_2_] | [P]_mit(F)_×2÷2 |  |  | 6.09 |  |  |  |  |  | 6.09 |  |
| Bol3 | 100% bound cluster bridging | 0.5 [Fe_2_S_2_] | [P]_mit(F)_×2÷2 |  |  | 2.34 |  |  |  |  |  | 2.34 |  |
| Ccp1 | 100% bound | 2 heme b | [P]_mit(F)_×2 |  |  |  |  | 20.32 |  |  |  | 20.32 |  |
| Coq7 | 100% bound | 1[Fe-O-Fe] | [P]_mit(F)_×2 |  |  |  |  |  |  |  | 4.36 | 4.36 |  |
| Cox10 | 100% bound  (uncertain) | 1 HS? heme o | [P]_mit(F)_ |  |  |  | 0.88 |  |  |  |  | 0.88 |  |
| Cox15 | 100% bound  (uncertain) | 1 HS? heme b and  1 HS? heme a | [P]_mit(F)_  and  [P]_mit(F)_ |  |  |  | 9.72 |  |  |  |  | 9.72 |  |
| Cyb2 | 100% bound | 1 LS heme b | [P]_mit(F)_ |  |  |  |  | 7.40 |  |  |  | 7.40 |  |
| Cyc1 | 100% bound | 1 LS heme c | [P]_mit(F)_ |  |  |  |  | 37.97 |  |  |  | 37.97 |  |
| Cyc7 | 100% bound | 1 LS heme c | [P]_mit(F)_ |  |  |  |  | 4.06 |  |  |  | 4.06 |  |
| Exo5 | 100% bound | 1 [Fe_4_S_4_] | [P]_mit(F)_×4 | 8.26 |  |  |  |  |  |  |  | 8.26 |  |
| Fre5 | 50% bound  (uncertain) | 2 LS heme b | [P]_mit(F)_×2÷2 |  |  |  |  | 2.65 |  |  |  | 2.65 |  |
| Grx5 | 100% bound cluster bridging | 0.5[Fe_4_S_4_]  and  1.5[Fe_2_S_2_] | [P]_mit(F)_×4÷2  and  [P]_mit(F)_×2×1.5 | 23.84 |  | 35.76 |  |  |  |  |  | 59.60 |  |
| Hem15 | 100% bound | 1 Fe^II^_O/N_ | [P]_mit(F)_ |  |  |  |  |  |  | 10.36 |  | 10.36 |  |
| Ilv3 | 100% bound | 1 [Fe_2_S_2_] | [P]_mit(F)_×2 |  |  | 107.96 |  |  |  |  |  | 107.96 |  |
| Isa1 | 100% bound | 0.5 [Fe_4_S_4_]  and  0.5 [Fe_2_S_2_] | [P]_mit(F)_×4÷2  and  [P]_mit(F)_×2÷2 | 3.70 |  | 1.85 |  |  |  |  |  | 5.55 |  |
| Isa2 | 100% bound | 0.5 [Fe_4_S_4_] + and  0.5 [Fe_2_S_2_] | [P]_mit(F)_×4÷2  and  [P]_mit(F)_×2÷2 | 3.46 |  | 1.73 |  |  |  |  |  | 5.19 |  |
| Isu1 | 100% bound | 1 [Fe_2_S_2_] | [P]_mit(F)_×2 |  |  | 10.50 |  |  |  |  |  | 10.50 |  |
| Isu2 | 100% bound | 1 [Fe_2_S_2_] | [P]_mit(F)_×2 |  |  | 5.58 |  |  |  |  |  | 5.58 |  |
| Lip5 | 100% bound | 2 [Fe_4_S_4_] | [P]_mit(F)_×4×2 | 22.96 |  |  |  |  |  |  |  | 22.96 |  |
| Lys4 | 100% bound | 1 [Fe_4_S_4_] | [P]_mit(F)_×4 | 52.72 |  |  |  |  |  |  |  | 52.72 |  |
| Mss51 | 100% bound  (uncertain) | 1 HS? heme b | [P]_mit(F)_ |  |  |  | 5.21 |  |  |  |  | 5.21 |  |
| Nfu1 | 100% bound | 0.5 [Fe_4_S_4_] | [P]_mit(F)_×4÷2 | 21.80 |  |  |  |  |  |  |  | 21.80 |  |
| Thi4 | 100% bound | Fe^II^_(N/O)_ | [P]_mit(F)_ |  |  |  |  |  |  | 3.13 |  | 3.13 |  |
| Thi5 | 100% bound | Fe^II^_(N/O)_ | [P]_mit(F)_ |  |  |  |  |  |  | 1.26 |  | 1.26 |  |
| Yah1 | 100% bound | 1 [Fe_2_S_2_] | [P]_mit(F)_×2 |  |  | 5.26 |  |  |  |  |  | 5.26 |  |
| Yfh1 | 50% bound  (uncertain) | 1 Fe^II^_O/N_ | [P]_mit(F)_÷2 |  |  |  |  |  |  | 1.40 |  | 1.40 |  |
| **Non-selected entries total** |  |  |  | 555.42 | 0 | 185.25 | 15.81 | 72.4 | 0 | 16.15 | 4.36 | 849.39 |  |
|  |  |  |  |  |  |  |  |  |  |  |  |  |  |
| **Selected**  **Entries** |  |  |  |  |  |  |  |  |  |  |  |  |  |
| Cox1 | 100% bound | 1 LS heme a  and  1 HS heme a3 | [P]_mit(Fsa)_  and  [P]_mit(Fsa)_ |  |  |  | 7.93 | 7.93 |  |  |  | 15.86 |  |
| Cob1 | 100% bound | 2 heme b | [P]_mit(Fsa)_×2 |  |  |  |  | 34.8 |  |  |  | 34.8 |  |
| Cyt1 | 100% bound | 1 heme c | [P]_mit(Fsa)_ |  |  |  |  | 17.4 |  |  |  | 17.4 |  |
| Rip1 | 100% bound | 1 [Fe_2_S_2_] (2 His ligands) | [P]_mit(Fsa)_×2 |  |  | 34.8 |  |  |  |  |  | 34.8 |  |
| Sdh2 | 100% bound | 1 [Fe_4_S_4_]  and  1[Fe_3_S_4_]  and  1[Fe_2_S_2_] | [P]_mit(Fsa)_×4  and  [P]_mit(Fsa)_×3  and  [P]_mit(Fsa)_×2 | 30.12 | 22.59 | 15.06 |  |  |  |  |  | 67.77 |  |
| Sdh3 | 100% bound bridging heme with Sdh4 | 0.5 heme b | [P]_mit(Fsa)_÷2 |  |  |  |  | 3.76 |  |  |  | 3.76 |  |
| Sdh4 | 100% bound bridging heme with Sdh3 | 0.5 heme b | [P]_mit(Fsa)_÷2 |  |  |  |  | 3.76 |  |  |  | 3.76 |  |
| Selected entries total |  |  |  | 30.12 | 22.59 | 49.86 | 7.93 | **67.65** | 0 | 0 | 0 | 178.15 |  |
|  |  |  |  |  |  |  |  |  |  |  |  |  |  |
| **Totals (Fsa)** |  |  |  | **585.54** | **22.59** | **235.11** | **23.74** | **140.05** | 0.000 | **16.15** | **4.36** | 1027.54 |  |
| **Fraction** |  |  |  | **0.570** | **0.022** | **0.229** | **0.022** | **0.136** | **0** | **0.016** | **0.004** | 1.000 |  |
|  |  |  |  |  |  |  |  |  |  |  |  |  |  |
| **Mitochondria (Rsa)** |  | **Iron center(s)** | **Manipulation of [P]_mit(R)_** | **[Fe_4_S_4_]** | **[Fe_3_S_4_]** | **[Fe_2_S_2_]** | **H.S. Heme** | **L.S. Heme** | **Fe^II^_S_** | **Fe^II^_O/N_** | **Fe-O-Fe** | **Mito. [Iron]** |  |
| Aco1 | 100% metallated | 1[Fe_4_S_4_] | [P]_mit(R)_×4 | 240.44 |  |  |  |  |  |  |  | 240.44 |  |
| Aco2 | 100% metallated | 1[Fe_4_S_4_] | [P]_mit(R)_×4 | 6.50 |  |  |  |  |  |  |  | 6.50 |  |
| Aim32 | 100% bound  monomer | 1 [Fe_2_S_2_] | [P]_mit(R)_×2 |  |  | 0.40 |  |  |  |  |  | 0.40 |  |
| Bio2 | 100% bound | 1[Fe_4_S_4_]  and  1[Fe_2_S_2_] | [P]_mit(R)_×4  +  [P]_mit(R)_×2 | 1.56 |  | 0.78 |  |  |  |  |  | 2.34 |  |
| Bol1 | 100% bound cluster bridging | 0.5 [Fe_2_S_2_] | [P]_mit(R)_×2÷2 |  |  | 5.52 |  |  |  |  |  | 5.52 |  |
| Bol3 | 100% bound cluster bridging | 0.5 [Fe_2_S_2_] | [P]_mit(R)_×2÷2 |  |  | 0.60 |  |  |  |  |  | 0.60 |  |
| Ccp1 | 100% bound | 2 heme b | [P]_mit(R)_×2 |  |  |  |  | 14.38 |  |  |  | 14.38 |  |
| Coq7 | 100% bound | 1[Fe-O-Fe] | [P]_mit(R)_×2 |  |  |  |  |  |  |  | 0.29 | 0.29 |  |
| Cox10 | 100% bound  (uncertain) | 1 HS? heme o | [P]_mit(F)_ |  |  |  | 0.88 |  |  |  |  | 0.88 |  |
| Cox15 | 100% bound  (uncertain) | 1 HS? heme b and  1 HS? heme a | [P]_mit(R)_  and  [P]_mit(R)_ |  |  |  | 2.44 |  |  |  |  | 2.44 |  |
| Cyb2 | 100% bound | 1 LS heme b | [P]_mit(R)_ |  |  |  |  | 19.21 |  |  |  | 19.21 |  |
| Cyc1 | 100% bound | 1 LS heme c | [P]_mit(R)_ |  |  |  |  | 61.04 |  |  |  | 61.04 |  |
| Cyc7 | 100% bound | 1 LS heme c | [P]_mit(R)_ |  |  |  |  | 1.50 |  |  |  | 1.50 |  |
| Exo5 | 100% bound | 1 [Fe_4_S_4_] | [P]_mit(F)_×4 | 8.26 |  |  |  |  |  |  |  | 8.26 |  |
| Fre5 | 50% bound  (uncertain) | 2 LS heme b | [P]_mit(F)_×2÷2 |  |  |  |  | 2.65 |  |  |  | 2.65 |  |
| Grx5 | 100% bound cluster bridging | 0.5[Fe_4_S_4_]  and  1.5[Fe_2_S_2_] | [P]_mit(R)_×4÷2  and  [P]_mit(R)_×2×1.5 | 14.07 |  | 21.10 |  |  |  |  |  | 35.17 |  |
| Hem15 | 100% bound | 1 Fe^II^_O/N_ | [P]_mit(R)_ |  |  |  |  |  |  | 4.47 |  | 4.47 |  |
| Ilv3 | 100% bound | 1 [Fe_2_S_2_] | [P]_mit(R)_×2 |  |  | 28.02 |  |  |  |  |  | 28.02 |  |
| Isa1 | 100% bound | 0.5 [Fe_4_S_4_]  and  0.5 [Fe_2_S_2_] | [P]_mit(F)_×4÷2  and  [P]_mit(F)_×2÷2 | 3.70 |  | 1.85 |  |  |  |  |  | 5.55 |  |
| Isa2 | 100% bound | 0.5 [Fe_4_S_4_] + and  0.5 [Fe_2_S_2_] | [P]_mit(R)_×4÷2  and  [P]_mit(R)_×2÷2 | 0.26 |  | 0.13 |  |  |  |  |  | 0.39 |  |
| Isu1 | 100% bound | 1 [Fe_2_S_2_] | [P]_mit(R)_×2 |  |  | 1.33 |  |  |  |  |  | 1.33 |  |
| Isu2 | 100% bound | 1 [Fe_2_S_2_] | [P]_mit(R)_×2 |  |  | 0.04 |  |  |  |  |  | 0.04 |  |
| Lip5 | 100% bound | 2 [Fe_4_S_4_] | [P]_mit(R)_×4×2 | 2.77 |  |  |  |  |  |  |  | 2.77 |  |
| Lys4 | 100% bound | 1 [Fe_4_S_4_] | [P]_mit(R)_×4 | 4.84 |  |  |  |  |  |  |  | 4.84 |  |
| Mss51 | 100% bound  (uncertain) | 1 HS? heme b | [P]_mit(R)_ |  |  |  | 1.32 |  |  |  |  | 1.32 |  |
| Nfu1 | 100% bound | 0.5 [Fe_4_S_4_] | [P]_mit(R)_×4÷2 | 9.88 |  |  |  |  |  |  |  | 9.88 |  |
| Thi4 | 100% bound | Fe^II^_(N/O)_ | [P]_mit(F)_ |  |  |  |  |  |  | 3.13 |  | 3.13 |  |
| Thi5 | 100% bound | Fe^II^_(N/O)_ | [P]_mit(F)_ |  |  |  |  |  |  | 1.26 |  | 1.26 |  |
| Yah1 | 100% bound | 1 [Fe_2_S_2_] | [P]_mit(R)_×2 |  |  | 0.44 |  |  |  |  |  | 0.44 |  |
| Yfh1 | 50% bound  (uncertain) | 1 Fe^II^_O/N_ | [P]_mit(R)_÷2 |  |  |  |  |  |  | 0.29 |  | 0.29 |  |
| **Non-selected entries total** |  |  |  | 292.28 | 0 | 60.21 | 4.64 | 98.78 | 0 | 9.15 | 0.29 | 465.35 |  |
|  |  |  |  |  |  |  |  |  |  |  |  |  |  |
| **Selected**  **Entries** |  |  |  |  |  |  |  |  |  |  |  |  |  |
| Cox1 | 100% bound | 1 LS heme a  and  1 HS heme a3 | [P]_mit(Rsa)_  and  [P]_mit(Rsa)_ |  |  |  | 11.4 | 11.4 |  |  |  | 22.8 |  |
| Cob1 | 100% bound | 2 heme b | [P]_mit(Rsa)_×2 |  |  |  |  | 45.8 |  |  |  | 45.8 |  |
| Cyt1 | 100% bound | 1 heme c | [P]_mit(Rsa)_ |  |  |  |  | 22.9 |  |  |  | 22.9 |  |
| Rip1 | 100% bound | 1 [Fe_2_S_2_] (2 His ligands) | [P]_mit(Rsa)_×2 |  |  | 45.8 |  |  |  |  |  | 45.8 |  |
| Sdh2 | 100% bound | 1 [Fe_4_S_4_]  and  1[Fe_3_S_4_]  and  1[Fe_2_S_2_] | [P]_mit(Rsa)_×4  and  [P]_mit(Rsa)_×3  and  [P]_mit(Rsa)_×2 | 59.6 | 44.7 | 29.8 |  |  |  |  |  | 134.1 |  |
| Sdh3 | 100% bound bridging heme with Sdh4 | 0.5 heme b | [P]_mit(Rsa)_÷2 |  |  |  |  | 7.45 |  |  |  | 7.45 |  |
| Sdh4 | 100% bound bridging heme with Sdh3 | 0.5 heme b | [P]_mit(Rsa)_÷2 |  |  |  |  | 7.45 |  |  |  | 7.45 |  |
| Selected entries total |  |  |  | 59.6 | 44.7 | 75.6 | 11.4 | 95.0 | 0 | 0 | 0 | 286.3 |  |
|  |  |  |  |  |  |  |  |  |  |  |  |  |  |
| **Totals (Rsa)** |  |  |  | **351.88** | **44.7** | **135.81** | **16.04** | **193.78** | 0.000 | **9.15** | **0.29** | 751.65 |  |
| **Fraction** |  |  |  | **0.468** | **0.059** | **0.181** | **0.021** | **0.258** | **0** | **0.012** | **0.000** | 1.000 |  |
|  |  |  |  |  |  |  |  |  |  |  |  |  |  |
| **Cell concentrations for nonmitochondrial compartments** | | | | |  |  |  |  |  |  |  |  |  |
| Cytosol (×0.615) |  |  |  | 27.31 | 1.99 | 1.67 | 2.37 | 0.33 | 0.43 | 1.20 | 2.48 | 37.78 |  |
| Nucleus (×0.125) |  |  |  | 2.90 | 0 | 0.07 | 0 | 0.04 | 0 | 0.32 | 0 | 3.34 |  |
| ER (×0.015) |  |  |  | 0 | 0 | 0.10 | 0.27 | 2.27 | 0 | 0.08 | 4.16 | 6.87 |  |
| Vacuole (×0.068) |  |  |  | 0 | 0 | 0 | 0 | 0.08 | 0 | 0 | 0 | 0.08 |  |
| [Fe]_cell_; sum of non-mito |  |  |  | 30.21 | 1.99 | 1.85 | 2.64 | 2.72 | 0.43 | 1.60 | 6.64 | 48.07 |  |
|  |  |  |  |  |  |  |  |  |  |  |  |  |  |
| **Cell concentrations including mitochondrial compartments (Fsa)** | | | | |  |  |  |  |  |  |  |  |  |
| Mito. (Fsa) (×0.033) |  |  |  | 19.32 | 0.74 | 7.76 | 0.78 | 4.62 | 0 | 0.53 | 0.14 | 33.90 |  |
| Sum with non-mitos |  |  |  | 49.53 | 2.73 | 9.61 | 3.42 | 7.34 | 0.43 | 2.13 | 6.78 | 81.97 |  |
| Fraction (cell) |  |  |  | 0.604 | 0.033 | 0.117 | 0.042 | 0.090 | 0.005 | 0.026 | 0.083 | 1.000 |  |
|  |  |  |  |  |  |  |  |  |  |  |  |  |  |
| **Fas Cell concentrations including LFePs (iron replete)** | | | | |  |  |  |  |  |  |  |  |  |
|  |  |  | NHHS Fe^III^ |  |  |  |  |  |  | NHHS Fe^II^ |  |  |  |
| LFeP |  |  | 200 |  |  |  |  |  |  | 15 |  |  |  |
| Sum |  |  | 200 | 49.53 | 2.73 | 9.61 | 3.42 | 7.34 | 0.43 | 17.13 | 6.78 | 296.97 |  |
| Fraction (cell) |  |  | 0.673 | 0.167 | 0.009 | 0.032 | 0.012 | 0.025 | 0.001 | 0.058 | 0.023 | 1.000 |  |
| **Cell concentrations including mitochondrial compartments (Rsa)** | | | | |  |  |  |  |  |  |  |  |  |
| Mito. (Rsa) (×0.10) |  |  |  | 35.19 | 4.47 | 13.58 | 1.60 | 19.38 | 0 | 0.92 | 0.03 | 75.16 |  |
| Sum with non-mitos |  |  |  | 65.40 | 6.46 | 15.43 | 4.24 | 22.10 | 0.43 | 2.52 | 6.67 | 123.23 |  |
| Fraction (cell) |  |  |  | 0.531 | 0.052 | 0.125 | 0.034 | 0.179 | 0.003 | 0.020 | 0.054 | 1.000 |  |
|  |  |  |  |  |  |  |  |  |  |  |  |  |  |
| **Ras Cell concentrations including LFePs (iron replete)** | | | | |  |  |  |  |  |  |  |  |  |
|  |  |  | NHHS Fe^III^ |  |  |  |  |  |  | NHHS Fe^II^ |  |  |  |
| LFeP |  |  | 200 |  |  |  |  |  |  | 15 |  |  |  |
| Sum |  |  | 200 | 65.40 | 6.46 | 15.43 | 4.24 | 22.10 | 0.43 | 17.52 | 6.67 | 338.25 |  |
| Fraction (cell) |  |  | 0.591 | 0.193 | 0.019 | 0.046 | 0.012 | 0.065 | 0.001 | 0.052 | 0.020 | 1.000 |  |

**Table S4: Local subunit-average concentrations of selected mitochondrial proteins (iron-containing respiratory complexes)** **under F and R conditions.**

| Name | Identifier | CPC [9, 127] | Cellular Concentrations (µM); [P]_cell(F)_ | Local concentration (µM);  [P]_mit(F)_ = [P]_cell(F)_ ÷ 0.033  [P]_mit(R)_ = [P]_cell(R)_ ÷ 0.100 |
| --- | --- | --- | --- | --- |
| **Cytochrome c oxidase** |  |  |  |  |
| Cox1 | Q0045 | 2382 (F Ho)  1200 (F Mo)  9596 (R Mo) | 0.09418  0.0474  0.0708 (ave F)  0.3794 (R) | 2.854  1.436  2.14 (ave F)  3.79 (R) |
| Cox2 | Q0250 | 4411 (F Ho)  4551 (F Mo)  32993 (R Mo) | 0.1744  0.1799  0.177 (ave F)  1.304 (R) | 5.285  5.452  5.37 (Ave F)  13.04 (R) |
| Cox3 | Q0275 | ---  NA | --- | --- |
| Cox4 | YGL187C | 18243 (F Ho)  25566 (F Mo)  91731 (R Mo) | 0.7213  1.0108  0.866 (ave F)  3.627 (R) | 21.86  30.63  26.2 (ave F)  36.27 (R) |
| Cox5A | YNL052W | 4751 (F Ho)  4040 (F Mo)  28532 (R Mo) | 0.1878  0.1597  0.174 (Ave F)  1.128 (R) | 5.691  4.839  5.265 (Ave F)  11.28 (R) |
| Cox5B | YIL111W | 3876 (F Ho)  521 (F Mo)  341 (R Mo) | 0.1532  0.0206  0.0869 (Ave F)  0.0135 (R) | 4.642  0.6242  2.633 (ave F)  0.135 (R) |
| Cox6 | YHR051W | 13358 (F Ho)  18309 (F Mo)  66753 (R Mo) | 0.5281  0.724  0.626 (Ave F)  2.639 (R) | 16.00  21.94  18.97 (Ave F)  26.39 (R) |
| Cox7 | YMR256C | 4694 (F Ho)  834 (F Mo)  3240 (R Mo) | 0.1856  0.0330  0.202 (Ave F)  0.1281 (R) | 5.624  1.00  3.31 (Ave F)  1.281 (R) |
| Cox9 | YDL067C | 8954 (F Ho)  4921 (F Mo)  25127 (R Mo) | 0.3540  0.1945  0.274 (Ave F)  0.9935 (R) | 10.73  5.894  8.31 (Ave F)  9.935 (R) |
| Cox12 | YLR038C | 8598 (F Ho)  7028 (F Mo)  42099 (R Mo) | 0.3399  0.2779  0.309 (Ave F)  1.664 (R) | 10.03  8.421  9.23 (Ave F)  16.64 (R) |
| Cox13 | YGL191W | 4996 (F Ho)  2406 (F Mo)  17934 (R Mo) | 0.1975  0.0951  0.146 (Ave F)  0.7091 (R) | 5.985  2.882  4.43 (Ave F)  7.091 (R) |
| Cox8 (2 copies per complex) | YLR395C | 3909/2 = 1955 (F Ho)  587/2 =293 (F Mo)  3433/2 = 1716 (R Mo) | 0.0773  0.0116  0.0444 (Ave F)  0.0679 (R) | 2.342  0.352  1.347 (Ave F)  0.679 (R) |
| Average (± SD) |  |  | 0.27 ± 0.24 (F)  1.15 ± 1.09 (R) | 7.93 ± 7.44 (Fsa)  11.4 ± 11.0 (Rsa) |
|  |  |  |  |  |
| **Cytochrome bc1** |  |  |  |  |
| Cob1 | Q0105 | 8283 (F Ho)  (NA) | 0.3275 (F) | 9.924 (F) |
| Cyt1 | YOR065W | 6247 (F Ho)  10878 (F Mo)  34499 (R Mo) | 0.2470  0.4300  0.338 (Ave F)  1.364 (R) | 7.485  13.03  10.26 (Ave F)  13.64 (R) |
| Rip1 | YEL024W | 10319 (F Ho)  9128 (F Mo)  35070 (R Mo) | 0.4080  0.3609  0.3844 (Ave F)  1.3866 (R) | 12.36  10.936  11.65 (Ave F)  13.866 (R) |
| Cor1 | YBL045C | 21181 (F Ho)  41221 (F Mo)  126771 (R Mo) | 0.8374  1.6298  1.234 (Ave F)  5.0122 (R) | 25.38  49.39  37.39 (Ave F)  50.122 (R) |
| Qcr2 | YPR191W | 31306 (F Ho)  48948 (F Mo)  154636 (R Mo) | 1.238  1.935  1.586 (Ave F)  6.114 (R) | 37.52  58.64  48.08 (Ave F)  61.14 (R) |
| Qcr6 | YFR033C | 6068 (F Ho)  11400 (F Mo)  21131 (R Mo) | 0.2399  0.4507  0.3453 (Ave F)  0.8355 (R) | 7.27  13.66  10.46 (Ave F)  8.355 (R) |
| Qcr7 | YDR529C | 21227 (F Ho)  19642 (F Mo)  82753 (R Mo) | 0.8393  0.7766  0.8080 (Ave F)  3.272 (R) | 25.43  23.53  24.48 (Ave F)  32.72 (R) |
| Qcr8 | YJL166W | 4551 (F Ho)  9641 (F Mo)  10020 (R Mo) | 0.1799  0.3812  0.2805 (Ave F)  0.3962 (R) | 5.452  11.55  8.502 (Ave F)  3.962 (R) |
| Qcr9 | YGR183C | 5276 (F Ho)  3141 (F Mo)  12638 (R Mo) | 0.2086  0.1242  0.1664 (Ave F)  0.4997 (R) | 6.321  3.764  5.042 (Ave F)  4.997 (R) |
| Qcr10 | YHR001W-A | 4479 (F Ho)  9215 (F Mo)  44548 (R Mo) | 0.1771  0.3643  0.271 (Ave F)  1.762 (R) | 5.367  11.04  8.203 (Ave F)  17.62 (R) |
| Average (± SD) |  |  | 0.57 ± 0.48 (F)  2.29 ± 1.94 (R) | 17.4 ± 13.8 (Fsa)  22.9 ± 19.4 (Rsa) |
|  |  |  |  |  |
| **Succinate Dehydrogenase** |  |  |  |  |
| Sdh1 | YKL148C | 9915 (F Ho)  6537 (F Mo)  33739 (R Mo) | 0.3920  0.2584  0.3252 (Ave F)  1.334 (R) | 11.88  7.83  9.855 (Ave F)  13.34 (R) |
| Sdh2 | YLL041C | 8535 (F Ho)  11429(F Mo)  85568 (R Mo) | 0.3374  0.4519  0.3946 (Ave F)  3.3832 (R) | 10.224  13.693  11.96 (Ave F)  33.832 (R) |
| Sdh3 | YKL141W | 4719 (F Ho)  2665(F Mo)  18383 (R Mo) | 0.1866  0.1054  0.1460 (Ave F)  0.7268 (R) | 5.654  3.194  4.424 (Ave F)  7.268 (R) |
| Sdh4 | YDR178W | 3115 (F Ho)  3377(F Mo)  12815 (R Mo) | 0.1232  0.1335  0.1284 (Ave F)  0.5067 (R) | 3.733  4.045  3.889 (Ave F)  5.067 (R) |
| Average (± SD) |  |  | 0.248 ± 0.114 (F)  1.49 ± 1.14 (R) | 7.53 ± 3.46 (Fsa)  14.9 ± 11.4 (Rsa) |

**Table S5:** **Comparison of simulated vs. observed iron content of fermenting and respiring mitochondria under iron-replete conditions.** Concentrations are in µM. Percentages and concentrations have been rounded.

| **Isolated Mitochondria** | **Simulation** | | **Observed** | |
| --- | --- | --- | --- | --- |
| **Proteins only** | **Fsa** | **Rsa** | **F** | **R** |
| [Fe]_cell_ + LFePs | 1030 µM | 750 µM | 770 µM [128] and 480 µM -840 µM [136] | 800 ± 200 µM [10] and 720-840 µM[128] and 690 µM [11] |
| Central Doublet | 71% | 73% | 25%[128] and 20% [134] | 50% – 60%[128] and 57% (390 µM) [11] |
| [Fe_2_S_2_] | 23% | 18% | 0%[128] and 24%[136] | 10% – 17%[128] and 11% (80 µM) [11] |
| HS Heme Fe^II^ | 2.2% | 2.1% | 4%[128] | 4% – 7%[128] and 2% (10 µM) [11] |
| NHHS Fe^II^ | 2% | 1.2% | 20% (150 µM) [128, 134] | 2% – 3%[128] and 3% – 7% [134] and 30% (210 µM) [11] |
| Nanoparticles/unassigned | N.I. | N.I. | 40% [134] | 0%[11] |
| NHHS Fe^III^ | 0 | 0 | 20% (150 µM) [134] | 0%[11] |
| Cytochrome c oxidase | 7.9 µM | 11 µM | 8 µM [128] | 6-20 µM[10]; 30–35 µM [128] |
| Succinate dehydrogenase | 7.5 µM | 15 µM | 2 µM [128] | 5 – 10 µM [128] |
| Cytochrome bc1 | 17 µM | 23 µM | 6 µM [128] | 10 – 20 µM [128] |
| “other [Fe_4_S_4_]” (aconitase) | 100 µM | 62 µM | 13 µM [128] | 36 – 55 µM [128] |
| [Heme Fe^II^] | 160 µM | 210 µM | 114 µM [128] | 223-276 µM [128] |
|  |  |  |  |  |
| **Whole Cells** | **Simulation** | | **Observed** | |
| **Proteins + LFePs** | **Fsa** | **Rsa** | **F** | **R** |
| [Fe]_cell_ + LFePs | 300 µM | 340 µM | 400–470 µM [136] and 440 µM [148] | 480-880 µM [11] |
| NHHS Fe^III^ | 67% (200 µM) | 59% (200 µM) | 75%-80% (300-380 µM [136] and 73% (330 µM) [148] | 75% [134] and 39%-52% (190-460 µM) [11] |
| Central Doublet | 12% (57 µM) | 17% (88 µM) | 10% -18% [136] and 5% (120 µM) [148] | 25%-39% (190-220 µM) [11] |
| NHHS Fe^II^ | 8% (24 µM) | 7% (24 µM) | 5% – 7% (23 – 26 µM) [136] and 10% (240 µM) [148] | 8%-11% (50-70 µM) [11] |
| [Fe_2_S_2_] | 2% (10 µM) | 3% (15 µM) | --- | 6%-9% (30-80 µM) [11] |
| HS Heme Fe^II^ | 1% (3 µM) | 1% (4 µM) | --- | 5%-6% (20-50 µM) [11] |
| nanoparticles/unassigned | N.I. | N.I. | 12% [148] | 0%[11] |

**Table S6:** Assumed thermodynamic reduction potentials for various cellular compartments.

| Compartment | E’compartment (mV vs NHE) | Ref. |
| --- | --- | --- |
| Vacuoles | -180 |  |
| ER | -220 | [131, 149 – 152] |
| Mitochondrial IMS | -250 | [153] |
| Nucleus | -290 | [132] |
| Cytosol | -300 | [132, 153 – 155] |
| Mitochondrial matrix | -320 | [10] |
| Peroxisomes | -380, pH 8 | [156] |

**Table S7: Mössbauer and Redox properties of each iron center in yeast.** E^0^’_ox/red_ values and MB parameters are estimates based on published results. In cases when the required information was unavailable, corresponding properties of homologs from other organisms were assumed. Other assumptions should be considered *informed hypotheses*. Due to the large number of entries, occasional errors are possible, so readers should refer to original articles to verify parameters and/or obtain additional relevant information or caveats. Please send corrections to [Lindahl@chem.tamu.edu](mailto:Lindahl@chem.tamu.edu). Very recently recognized iron-containing proteins (as indicated) were excluded in this table and in corresponding Mössbauer simulations, resulting in errors of 0.2%, 0.8%, and 0.4% of spectral intensity for cytosol, mitochondria, and nuclei, respectively.

| **Protein** | **Iron center(s)** | **Redox Properties (pH 7)**  **Potentials are mV vs NHE** | **Mössbauer properties (for 5 K, 0.05 T spectrum)** | **Assumptions** | **Reference** |
| --- | --- | --- | --- | --- | --- |
| **Cytosol** |  | E^0^’_cyt_ = -300 mV |  |  |  |
| Aft1 | [Fe_2_S_2_](S)_4_ | E^0^’[Fe_2_S_2_]^2+/1+^ = -600 mV (est) | Site 1: 50% S = 0; δ = 0.29 mm/s; ΔE_Q_ = 0.62 mm/s η = 0.75  Site 2: 50% S = 0; δ = 0.28 mm/s; ΔE_Q_ = 0.44 mm/s η = 0.75 | 100% S = 0 [Fe_2_S_2_]^2+^  0% S = ½ [Fe_2_S_2_]^1+^  Redox-inactive in the [Fe_2_S_2_]^2+^ state. | [21, 157, 158] |
| Aft2 | [Fe_2_S_2_](S)_4_ | E^0^’[Fe_2_S_2_]^2+/1+^ = -600 mV (est) | Site 1: 50% S = 0; δ = 0.29 mm/s; ΔE_Q_ = 0.62 mm/s η = 0.75  Site 2: 50% S = 0; δ = 0.28 mm/s; ΔE_Q_ = 0.44 mm/s η = 0.75 | 100% S = 0 [Fe_2_S_2_]^2+^  0% S = ½ [Fe_2_S_2_]^1+^  Redox-inactive in the [Fe_2_S_2_]^2+^ state. | [21, 157, 158] |
| Apd1 | [Fe_2_S_2_](S)_2_(N)_2_ | E^0^’ = -164 mV | Site 1: 50% S = 0; δ = 0.24 mm/s; ΔE_Q_ = -0.54 mm/s η = 0.6  Site 2: 50% S = 0; δ = 0.35 mm/s; ΔE_Q_ = 1.06 mm/s η = 0.3  Dithionite reduced  g = 2.009, 1.906, 1.861  Site 1: 50% S = 1/2; δ = 0.32 mm/s; ΔE_Q_ = +0.81 mm/s η = 0  Site 2: 50% S = 1/2; δ = 0.75 mm/s; ΔE_Q_ = -3.16 mm/s η = +3.0 |  | [22] |
| Bna1 | Fe^II^ | E^0’^(Fe^III^/Fe^II^) for TauD 2OG dioxygenase shows strong (468 mV) redox hysteresis;  E^0^ for Fe^III^ 🡪 Fe^II^ = -272 mV  E^0^ for Fe^II^ 🡪 Fe^III^ = + 196 mV | Site 1: 100% S = 2; δ = 1.28 mm/s; ΔE_Q_ = 2.22 mm/s. | 100% reduced Fe^II^ | [50, 159 – 162] |
| Bol2 | [Fe_2_S_2_](S)_2_(N)_2_ | The cluster in the BolA:Grx3 heterodimer can be reduced by dithionite.  E^0^’[Fe_2_S_2_]^2+/1+^ = -400 mV (est) | Site 1: 50%; δ = 0.30 mm/s; ΔE_Q_ = 0.50 mm/s; Γ = 0.32 mm/s  Site 2: 50%; δ = 0.30 mm/s; ΔE_Q_ = 0.82 mm/s; Γ = 0.32 mm/s  Extra: Dithionite reduced S = ½ [Fe_2_S_2_]^1+^ g = 2.01, 1.92, 1.87 | 100%[Fe_2_S_2_]^2+^ | [21, 74, 163 – 166] |
| Cfd1 | [Fe_4_S_4_](S)_4_ | Cluster can be reduced by dithionite to generate a bleached UV-vis spectrum and axial EPR signal with g = 2.041 and 1.93.  E^0’^[Fe_4_S_4_]^2+/1+^ = -400 mV (est) | Site 1: 100% S = 0 δ = 0.45 mm/s; ΔE_Q_ = 1.15 mm/s; Γ = 0.32 mm/s  Extra: Reduced S = ½ [Fe4S4]1+ cluster:  Site 1: 50%; δ = 0.52 mm/s; ΔE_Q_ = 1.0 mm/s; η = 0.6; A_xyz_ = -37 MHz; -25 MHz; -25 MHz  Site 2: 50%; δ = 0.58 mm/s; ΔE_Q_ = 1.6 mm/s; η = 0.5 A_xyz_ = +22 MHz; +17 MHz; +10 MHz | 100%[Fe_4_S_4_]^2+^ | [14] |
| Cta1 (peroxisomes) | HS heme b | E^0^ < -500 mV (est)  E(peroxisomes) = -380 mV | Site 1: 100% S = 5/2; δ = 0.40 mm/s; ΔE_Q_ = 1.00 mm/s; A_xyz_/(g_n_β_n_) = 21, 19, 18 T; D = 10 cm^-1^, g = 2.0, E/D = 0.01, Γ = 0.3 mm/s. | 100% [Fe^III^] | [167 – 171] |
| Ctt1 | HS heme b | E^0^ < -500 mV (est) | Site 1: 100% S = 5/2; δ = 0.40 mm/s; ΔE_Q_ = 1.00 mm/s; A_xyz_/(g_n_β_n_) = 21, 19, 18 T; D = 10 cm^-1^, g = 2.0, E/D = 0.01, Γ = 0.3 mm/s. | 100% [Fe^III^] | [167, 172, 173] |
| Dbr1 (not included) |  |  |  |  |  |
| Dph1 | [Fe_4_S_4_](S)_3_ R-SAM | The cluster can be reduced with dithionite.  E^0^ [Fe_4_S_4_]^2+/1+^ (for Epl3) = -430 mV to -540 mV (ave = -485 mV) | Site 1: 100% S = 0; δ = 0.44 mm/s, ΔE_Q_ = 1.13 mm/s | 100% [Fe_4_S_4_]^2+^ | [42, 45] |
| Dph2 | [Fe_4_S_4_](S)_3_ R-SAM | The cluster can be reduced with dithionite.  E^0^ [Fe_4_S_4_]^2+/1+^ (for Epl3) = -430 mV to -540 mV (ave = -485 mV) | Site 1: 100% S = 0; δ = 0.44 mm/s, ΔE_Q_ = 1.13 mm/s | 100% [Fe_4_S_4_]^2+^ | [42, 45, 174] |
| Dph3 | Fe^II^(S)_4_ | E0 (rubredoxins) = -80 to +40 mV; Ave = -20 mV (est) | Site 1: 100% S = 2; δ = 0.70 mm/s; ΔE_Q_ = -3.25 mm/s;  A_xyz_ = -20.1; -11.3; -33.4 MHz  D = 5.7 cm^-1^; E/D = 0.25;η= 0.75. | 100% [Fe^II^] | [40, 175 – 177] |
| Dph4 | Fe^II^(S)_4_ | E^0^ (rubredoxins) = -80 to +40 mV; Ave = -20 mV (est) | Site 1: 100% S = 2; δ = 0.70 mm/s; ΔE_Q_ = -3.25 mm/s;  A_xyz_ = -20.1; -11.3; -33.4 MHz  D = 5.7 cm^-1^; E/D = 0.25;η= 0.75. | 100% [Fe^II^] | [178] |
| Dre2 | M1/A site: [Fe_2_S_2_](S)_4_  M2/B site: [Fe_2_S_2_](S)_4_ or [Fe_4_S_4_](S)_4_  M2/B site could be [Fe_4_S_4_] based on EPR temp. dependence or [Fe_2_S_2_] based on Mossbauer. | M1/A cluster: E^0^’[Fe_2_S_2_]^2+/1+^  = -260 mV (est)  M2/B cluster:  E^0^’[Fe_X_S_X_]^2+/1+^  < -400 mV (est) | M1/A Site 1: 8.5% S = 0; δ = 0.26 mm/s; ΔE_Q_ = 0.57 mm/s Γ = 0.30 mm/s  M1/A Site 2: 20.75% S = ½ δ = 0.35 mm/s; ΔE_Q_ = 0.91 mm/s Γ = 0.30 mm/s η= 0.34; A_xyz_ = -41.1, -37.1, and -26.9 T  M1/A Site 3: 20.75% S = ½ δ = 0.70 mm/s; ΔE_Q_ = -3.24 mm/s Γ = 0.30 mm/s η = -3.2; A_xyz_ = +6.2, +19.8, +26.2 T  M2/B Site 4: 50% S = 0; δ = 0.28 mm/s; ΔE_Q_ = 0.39 mm/s Γ = 0.30 mm/s  Extra: M2/B site reduced: S = ½  Site 1: δ = 0.37 mm/s; ΔE_Q_ = 1.17 mm/s Γ = 0.30 mm/s eta = 0; Axyz = -35, -8.3, and -19.2 T  Site 2: δ = 0.71 mm/s; ΔE_Q_ = -3.57 mm/s Γ = 0.30 mm/s eta = 1 Axyz = +5.6, +16.1, +25.8 | M1/A cluster:  17% [ox]  83% [red]  M2/B Cluster  100% [ox] | [23, 179-182] |
| Elp3 | [Fe_4_S_4_] (S)_3_ R-SAM | E^0^ [Fe_4_S_4_]^2+/1+^ = -430 mV to -540 mV (ave = -485 mV)  Cluster reducible by dithionite. | Site 1: 100% S = 0; δ = 0.44 mm/s, ΔE_Q_ = 1.13 mm/s | 100% [Fe_4_S_4_]^2+^ | [183-188] |
| Fre1 | LS heme b (2 His)  (e.g. cytochrome b561)  Hemes are on opposite sides of the lipid bilayer | E^0^ = -250 mV | Site 1: 88% S = 0; δ = 0.4 mm/s; ΔE_Q_ = 1.1 mm/s.  Site 2: 12% S = ½; δ = 0.20 mm/s; ΔE_Q_ = 1.77 mm/s; η = -2; Ax,y,z = -40.3, +19.3, +53.5 MHz; g_xyx_ = 1.44, 2.27, 3.00. (same as Ccp1) | 88% [Fe^II^]  12% [Fe^III^] | [54, 189 – 192] |
| Fre2 | LS heme b (2 His)  (e.g. cytochrome b561)  Hemes are on opposite sides of the lipid bilayer | E^0^ = -250 mV | Site 1: 88% S = 0; δ = 0.4 mm/s; ΔE_Q_ = 1.1 mm/s.  Site 2: 12% S = ½; δ = 0.20 mm/s; ΔE_Q_ = 1.77 mm/s; η = -2; Ax,y,z = -40.3, +19.3, +53.5 MHz; g_xyx_ = 1.44, 2.27, 3.00. (same as Ccp1) | 88% [Fe^II^]  12% [Fe^III^] | [54, 189 – 192] |
| Fre3 | LS heme b (2 His)  (e.g. cytochrome b561)  Hemes are on opposite sides of the lipid bilayer | E^0^ = -250 mV (164) | Site 1: 88% S = 0; δ = 0.4 mm/s; ΔE_Q_ = 1.1 mm/s.  Site 2: 12% S = ½; S = ½; δ = 0.20 mm/s; ΔE_Q_ = 1.77 mm/s; η = -2; Ax,y,z = -40.3, +19.3, +53.5 MHz; g_xyx_ = 1.44, 2.27, 3.00. (same as Ccp1) | 88% [Fe^II^]  12% [Fe^III^] | [54, 189 – 192] |
| Fre4 | LS heme b (2 His)  (e.g. cytochrome b561)  Hemes are on opposite sides of the lipid bilayer | E^0^ = -250 mV (164) | Site 1: 88% S = 0; δ = 0.4 mm/s; ΔE_Q_ = 1.1 mm/s.  Site 2: 12% S = ½; S = ½; δ = 0.20 mm/s; ΔE_Q_ = 1.77 mm/s; η = -2; Ax,y,z = -40.3, +19.3, +53.5 MHz; g_xyx_ = 1.44, 2.27, 3.00. (same as Ccp1) | 88% [Fe^II^]  12% [Fe^III^] | [54, 189-192] |
| Fre7 | LS heme b (2 His)  (e.g. cytochrome b561)  Hemes on opposite sides of the lipid bilayer | E^0^ = -250 mV (164) | Site 1: 88% S = 0; δ = 0.4 mm/s; ΔE_Q_ = 1.1 mm/s.  Site 2: 12% S = ½; δ = 0.20 mm/s; ΔE_Q_ = 1.77 mm/s; η = -2; Ax,y,z = -40.3, +19.3, +53.5 MHz; g_xyx_ = 1.44, 2.27, 3.00. (same as Ccp1) | 88% [Fe^II^]  12% [Fe^III^] | [54, 189-192] |
| Glt1 | 2[Fe_4_S_4_] + 1 [Fe_3_S_4_] | E^0’^[Fe_3_S_4_]^1+/0^ = +460 mV (est)  E^0’^[Fe_4_S_4_]^2+/1+^ = -300 mV (est)  E^0’^[Fe_4_S_4_]^2+/1+^ = -600 mV (est) | [Fe_3_S_4_]^0^ cluster  Site 1: 18% (delocalized Fe^II^ Fe^III^ pair) S = 2; δ = 0.46 mm/s; ΔE_Q_ = 1.47 mm/s. η = 0.4; A_xyz_ = -20.5; -20.5, -16.4 MHz  Site 2: 9% (Fe^III^) S = 2; δ = 0.32 mm/s; ΔE_Q_ = -0.52 mm/s; η = -2; A_xyz_ = +13.7, +15.8, +15.6 MHz  [Fe_4_S_4_]^2+/1+^ cluster  Site 3: 18% S = 0; δ = 0.44 mm/s; ΔE_Q_ = 1.15 mm/sec.  Site 4: 9% S = ½ δ = 0.52 mm/s; ΔE_Q_ = 1.0 mm/s; η = 0.6;  A_xyz_ = -37 MHz; -25 MHz; -25 MHz  Site 5: 9% S = ½ δ = 0.58 mm/s; ΔE_Q_ = 1.6 mm/s; η = 0.5;  A_xyz_ = +22 MHz; +17 MHz; +10 MHz  [Fe_4_S_4_]^2+^ cluster  Site 6: 36% S = 0 18% S = 0; δ = 0.44 mm/s; ΔE_Q_ = 1.15 mm/sec. | 100% [Fe_3_S_4_]^0^  50% [Fe_4_S_4_]^2+^  50% [Fe_4_S_4_]^1+^  100% [Fe_4_S_4_]^2+^ | [28, 30, 193 – 197] |
| Grx3 | [Fe_2_S_2_](S)_2_(N)_2_ | E^0^’[Fe_2_S_2_]^2+/1+^ = -400 mV (est) | Site 1: 50%; S = 0; δ = 0.30 mm/s; ΔE_Q_ = 0.50 mm/s; Γ = 0.32 mm/s  Site 2: 50%; S = 0; δ = 0.30 mm/s; ΔE_Q_ = 0.82 mm/s; Γ = 0.32 mm/s  Extra: S = ½ [Fe_2_S_2_]^1+^ g = 2.01, 1.92, and 1.87 | 100%[Fe_2_S_2_]^2+^ | [21, 74, 163-165, 198, 199] |
| Grx4 | [Fe_2_S_2_](S)_2_(N)_2_ | E^0^’[Fe_2_S_2_]^2+/1+^ = -400 mV (est) | Site 1: 50%; S = 0; δ = 0.30 mm/s; ΔE_Q_ = 0.50 mm/s; Γ = 0.32 mm/s  Site 2: 50%; S = 0; δ = 0.30 mm/s; ΔE_Q_ = 0.82 mm/s; Γ = 0.32 mm/s  Extra: S = ½ [Fe_2_S_2_]^1+^ g = 2.01, 1.92, and 1.87 | 100%[Fe_2_S_2_]^2+^ | [21, 74, 163-165, 198, 199] |
| Leu1 | [Fe_4_S_4_] (S)_3_ | E0[Fe_4_S_4_]^2+/1+^ = -450 mV (aconitase) | Assume 50% citrate bound; 50% unbound  Site 1: 87.5% S = 0; δ = 0.45 mm/s; ΔE_Q_ = 1.30 mm/s.  Site 2; 6.25% S = 0; δ = 0.84 mm/s; ΔE_Q_ = 1.26 mm/s  Site 3: 6.25% ; S = 0; δ = 0.89 mm/s; ΔE_Q_ = 1.83 mm/s | 100% [Fe_4_S_4_]^2+^ | [26, 27, 200] |
| Lia1 | FeII(O/N) | Assume reduced | Assume 100% reduced; same parameters as AsqJ  S = 2; δ = 1.25 mm/s; ΔE_Q_ = 2.54 mm/s; Γ = 0.33 mm/s. | 100% Fe^II^_(O/N)_ | [47, 201] |
| Met5 | HS Fe^II^ siroheme+[Fe_4_S_4_] | E^0^(Siroheme) = -340 mV  E^0^ for Cluster = -405 mV | Site 1: 3.4%; S = 2; δ = 0.62 mm/s; ΔE_Q_ = 1.92 mm/s  Site 2: 16.6%; S = 5/2; δ = 0.45 mm/s; ΔE_Q_ = +1.0 mm/s;  A_0_ = -27 MHz; η = 0; D = +8 cm^-1^; E/D = 0.027.  Site 3: 13.6%%; S = 0; δ = 0.45 mm/s; ΔE_Q_ = 1.0 mm/s  Site 4: 33.2%; S = 5/2; δ = 0.45 mm/s; ΔE_Q_ = 1.0 mm/s  A0 = +6.4 MHz and η = -20  Site 5: 33.2%; S = 5/2; δ = 0.45 mm/s; ΔE_Q_ = 1.0 mm/s  A0 = -6.2 MHz and η = +30 | Siroheme:  83% [HS Fe^III^ heme]  17% [HS Fe^II^ heme]  100% [Fe_4_S_4_]^2+^ | [7, 31-33, 203-205] |
| Nar1 | [Fe_4_S_4_] | E^0’^[Fe_4_S_4_]^2+/1+^ = -400 mV (est) | Site 1: 100% S = 0 δ = 0.45 mm/s; ΔE_Q_ = 1.15 mm/s; Γ = 0.32 mm/s  Extra: S = ½ [Fe4S4]1+  50% Site 1: δ = 0.52 mm/s; ΔE_Q_ = 1.0 mm/s; n = 0.6; Axyz = -37 MHz; -25 MHz; -25 MHz  50% Site 2: δ = 0.58 mm/s; ΔE_Q_ = 1.6 mm/s; n = 0.5; Axyz = +22 MHz; +17 MHz; +10 MHz | 100% [Fe_4_S_4_]^2+^ | [206 – 208] |
| Nbp35 | [Fe_4_S_4_] (S)_4_ | E0’[Fe_4_S_4_]^2+/1+^ = -400 mV (est) | Site 1: 100% δ = 0.45 mm/s; ΔE_Q_ = 1.15 mm/s; Γ = 0.32 mm/s | 100% [Fe_4_S_4_]^2+^ | [209, 210] |
| Ncs6 | [Fe_3_S_4_] | Assumed like [Fe_3_S_4_] in Sdh2  E^0^ [Fe_3_S_4_]^1+/0^ = +62 mV | Site 1: 67% S = 2; δ = 0.46 mm/s; ΔEQ = 1.47 mm/s. eta = 0.4; Axyz = -20.5; -20.5, -16.4 MHz  Site 2 33%; S = 2; δ = 0.32 mm/s; ΔE_Q_ = -0.52 mm/s; eta = -2; Axyz = +13.7, +15.8, +15.6 | 100%[Fe_3_S_4_]^0^ | [46] |
| Rli1 | [Fe_4_S_4_] (S)_4_ | E^0’^[Fe_4_S_4_]^2+/1+^ = -600 mV (est) | Site 1: 50% S = 0; δ = 0.43 mm/s ΔEQ = 1.32 mm/s  Site 2: 50% S = 0; δ = 0.42 mms/s; ΔEQ = -0.86 mm/s | 100% [ox] | [34-36] |
| Rnr2 | [Fe-O-Fe] | E^0^ = -110 mV. | Site 1: S = 4; δ = 1.26 mm/s; ΔE_Q_ = 3.13 mm/s | 100% [Fe^II^] | [49, 211, 212] |
| Tdh3 | heme b (2 His) | E^0^ = -250 mV. | Site 1: 100% S = 0; δ = 0.4 mm/s; ΔE_Q_ = 1.1 mm/s. | 100% [Fe^II^] | [55, 192] |
| Tyw1 | [Fe_4_S_4_] (S)_3_ R-SAM | E^0’^[Fe_4_S_4_]^2+/1+^ = -485 mV (est)  E^0’^[Fe_4_S_4_]^2+/1+^ = -400 mV (est) | Site 1 (RS) 50% S = 0; δ = 0.44 mm/s; ΔEQ = 1.13 mm/s  Site 2 (P) 25% S = 0; δ = 0.46 mm/s; ΔE_Q_ = 1.07 mm/s  Site 3 (P) 12.5% S = 0; δ = 0.39 mm/s; ΔE_Q_ = 0.92 mm/s.  Site 4 (P) 12.5% S = 0; δ = 0.82 mm/s; ΔE_Q_ = 2.12 mm/s.  Extra: g = 2.05, 1.93, 1.84; and g = 2.05, 1.93, 1.83 | 100% [Fe_4_S_4_]^2+^  100% [Fe_4_S_4_]^2+^ | [45] |
| Yhb1 | Fe^II^ heme b | E^0^ = -280 mV (est) | Site 1: 69% S = 2; δ = 0.94 mm/s; ΔE_Q_ = 2.35 mm/s.  Site 2: 31% S = 5/2; δ = 0.40 mm/s, ΔE_Q_ = 1.00 mm/s; A_xyz_/(g_n_β_n_) = 21, 19, 18 T, D = 10 cm^-1^, g = 2.0, E/D = 0.01, g = 2.0, Γ = 0.3 mm/s. | 31% [Fe^III^]  69% [Fe^II^] | [52, 213, 214] |
|  |  |  |  |  |  |
| **Mitochondria** |  |  |  |  |  |
| **Protein** | **Iron center(s) per monomer** | **E’_mito-matrix_ = -320 mV**  **E’_mito-IMS_ = -250 mV** | **Mössbauer properties** |  | **Reference** |
| Aco1 | 1[Fe_4_S_4_] | E^0^ = -450 mV (matrix) | Assume 50% citrate bound; 50% unbound  Site 1: 87.5% S = 0; δ = 0.45 mm/s; ΔE_Q_ = 1.30 mm/s.  Site 2; 6.25% S = 0; δ = 0.84 mm/s; ΔE_Q_ = 1.26 mm/s  Site 3: 6.25% ; S = 0; δ = 0.89 mm/s; ΔE_Q_ = 1.83 mm/s | 100% [Fe_4_S_4_]^2+^ | [27, 63, 215] |
| Aco2 | [Fe_4_S_4_] | Same as Aco1  E^0^ = -450 mV (matrix) | Same as Aco1 | 100% [Fe_4_S_4_]^2+^ | [216] |
| Aim32  (not included) | [Fe_2_S_2_](S)_2_(N)_2_ | E^0^’ = -164 mV | Site 1: 50% S = 0; δ = 0.24 mm/s; ΔE_Q_ = -0.54 mm/s η = 0.6  Site 2: 50% S = 0; δ = 0.35 mm/s; ΔE_Q_ = 1.06 mm/s η = 0.3  Dithionite reduced  g = 2.011, 1.903, 1.860  Site 1: 50% S = 1/2; δ = 0.32 mm/s; ΔE_Q_ = +0.81 mm/s η = 0  Site 2: 50% S = 1/2; δ = 0.75 mm/s; ΔE_Q_ = -3.16 mm/s η = +3.0 | Same as Apd1 except g values | [22] |
| Bio2 | 1[Fe_4_S_4_] + 1[Fe_2_S_2_] | E^0^ [Fe_4_S_4_]^2+/1+^ = -440 mV (matrix)  E^0^ [Fe_2_S_2_] = -140 mV | [Fe_4_S_4_]^2+^  Site 1: 33% S = 0; δ = 0.45 mm/s; ΔE_Q_ = 1.28 mm/s  Site 2: 33% S = 0; δ = 0.44 mm/s; ΔE_Q_ = 1.03 mm/s  [Fe_2_S_2_]^2+^ cluster Site 3: 33% S = 0; δ = 0.28 mm/s; ΔE_Q_ = 0.50 mm/s | 100% [Fe_4_S_4_]^2+^  100% [Fe_2_S_2_]^2+^ | [75, 217] |
| Bol1 | [Fe_2_S_2_] | Assume same as Rip1  E^0^ = +285 mV (uncertain) | Site 1: 25%; S = ½; δ = 0.31 mm/s ΔE_Q_ = 0.63 mm/s; η = 0; A_xyz_ = 55, 50, and 43 MHz.  Site 2: 25% S = ½ δ = 0.74 mm/s ΔE_Q_ = 3.05 mm/s; η = 0;  A_xyz_ = 11, 14, and 33 MHz.  Site 3: 25% S = 0; δ = 0.24 mm/s ΔE_Q_ = 0.52 mm/s  Site 4: 25% S = 0; δ = 0.32 mm/s ΔE_Q_ = 0.91 mm/s | 50% [Fe_2_S_2_]^1+^  50% [Fe_2_S_2_]^2+^ | [69, 74, 218, 219] |
| Bol3 | [Fe_2_S_2_] | Assume same as Rip1  E^0^ = +285 mV (uncertain) | Same as Bol1:  Site 1: 25%; S = ½; δ = 0.31 mm/s ΔE_Q_ = 0.63 mm/s; η = 0; A_xyz_ = 55, 50, and 43 MHz.  Site 2: 25% S = ½ δ = 0.74 mm/s ΔE_Q_ = 3.05 mm/s; η = 0;  A_xyz_ = 11, 14, and 33 MHz.  Site 3: 25% S = 0; δ = 0.24 mm/s ΔE_Q_ = 0.52 mm/s  Site 4: 25% S = 0; δ = 0.32 mm/s ΔE_Q_ = 0.91 mm/s | 50% [Fe_2_S_2_]^1+^  50% [Fe_2_S_2_]^2+^ | [69, 74, 218, 219] |
| Ccp1 | heme c | E^0^ = -182 mV (in IMS) | Site 1: 50%; S = ½; δ = 0.26 mm/s; ΔE_Q_ = 2.50 mm/s; η = -2; Ax,y,z = -40.3, +19.3, +53.5 MHz; gxyx = 1.44, 2.27, 3.00.  Site 2: 50%; S = 0; δ = 0.45 mm/s; ΔE_Q_ = 1.2 mm/s | 50% [Fe^III^]  50% [Fe^II^] | [220, 221] |
| Cob1 | 2 heme b  Low spin  Bis heme | E^0^ (bH) ≈ -5 mV  E^0^ (bL) ≈ -150 mV  (connected to IMS) | Site 1: 75% S = 0; δ = 0.43 mm/s; ΔE_Q_ = 1.12 mm/s  Site 2: 25% S = ½ δ = 0.20 mm/s; ΔE_Q_ = 1.77 mm/s; η = -2; Ax,y,z = -40.3, +19.3, +53.5 MHz; gxyx = 1.44, 2.27, 3.00. (same as Ccp1) | 100% [Fe^II^] b_H_  50% [Fe^III^] b_L_  50% [Fe^II^] b_L_ | [60, 222 – 226] |
| Coq7 | [Fe-O-Fe] | E^0^ = + 3 mV (IM facing matrix). | Site 1: 100%; S = 4; δ = 1.30 mm/s; ΔE_Q_ = 3.14 mm/s | 100% [Fe^II^-O-Fe^II^] | [227 – 232] |
| Cox1 | 1 heme a; 1 heme a3 | E^0^ (a) = +255 mV  E^0^ (a_3_) = +350 mV  Assumed to be in redox equilibrium with the IMS | Site 1: 50% S = 2; δ = 0.93 mm/s; ΔE_Q_ = 1.85 mm/s  Site 2: 50%; S = 0; δ = 0.43 mm/s; ΔE_Q_ = 1.03 mm/s | 100% Fe^II^ (a)  100% Fe^II^ (a3) | [56, 57, 233 – 235] |
| Cox10 | 1 heme o | E0 = +160 mV (same as Cox15) | Site 1: 100% S = 2; δ = 0.93 mm/s; ΔE_Q_ = 1.85 mm/s  (uncertain) | 100% [Fe^II^] |  |
| Cox15 | 1 heme b + 1 heme a | E0 = +160 mV (uncertain) | Site 1: 100% S = 2; δ = 0.93 mm/s; ΔE_Q_ = 1.85 mm/s  (uncertain) | 100% [Fe^II^] | [140, 236] |
| Cyb2 | 1 LS heme b | E^0^ = -13 mV | Site 1: 100% S = 0; δ = 0.45 mm/s; ΔE_Q_ = 1.17 mm/s | 100% [Fe^II^] | [66, 237 – 244] |
| Cyc1 | 1 heme c | E^0^ = +290 mV | Site 1: 100%; S = 0; δ = 0.45 mm/s; ΔE_Q_ = 1.2 mm/s  Extra: S = ½ Fe^III^ heme: δ = 0.30 mm/s; ΔE_Q_ = 1.77 mm/s; n = 0; A/g_n_β_n_ (T) = -26.1; -4.0; 92.6 | 100% [Fe^II^] | [64, 245-247] |
| Cyc7 | 1 heme c | E0 = +286 | (Same as Cyc1)  Site 1: 100%; S = 0; δ = 0.45 mm/s; ΔE_Q_ = 1.2 mm/s | 100% [Fe^II^] | [65] |
| Cyt1 | 1 heme c. | E^0^ = +230 mV | LS Fe^II^ heme: δ = 0.45 mm/s; ΔE_Q_ = 1.17 mm/s | 100% [Fe^II^] | [60, 248-250] |
| Exo5 | 1 [Fe_4_S_4_] (4 cys) | E^0^ = – 700 mV | Site 1: 100% S = 0; δ = 0.44 mm/s; ΔE_Q_ = 1.13 mm/s | 100% [Fe_4_S_4_]^2+^ | [85] |
| Fre5 | 1 heme b | E^0^ = -250 mV | Same as Fre1  Site 1: 12% S = ½; δ = 0.20 mm/s; ΔE_Q_ = 1.77 mm/s; η = -2; Ax,y,z = -40.3, +19.3, +53.5 MHz; gxyx = 1.44, 2.27, 3.00. (same as Ccp1)  Site 2: 88% S = 0; δ = 0.4 mm/s; ΔE_Q_ = 1.1 mm/s. | 12% [FeIII]  88% [FeII] | [54, 189-192] |
| Grx5 | 0.5[Fe_4_S_4_] + 1.5[Fe_2_S_2_] | E^0^ = – 700 mV  Homodimer cluster not reducible. | Site 1: 40% S = 0; δ = 0.44 mm/s; ΔEQ = 1.13 mm/s  Site 2: 60% S = 0; δ = 0.28 mm/s; ΔE_Q_ = 0.50 mm/s | 100% [Fe_4_S_4_]^2+^  100% [Fe_2_S_2_]^2+^ | [72, 163, 198, 251] |
| Hem15 | Fe^II^_O/N_ | E^0’^= -160 mV (uncertain) | Site 1: 100% δ = 1.36 mm/s; ΔE_Q_ = 3.04 mm/s; | 100% [Fe^II^] | [83, 252, 253] |
| Ilv3 | [Fe_2_S_2_] | E^0^ = -400 mV (uncertain) | Site 1: 100% S = 0 δ = 0.28 mm/s; ΔE_Q_ = 0.50 mm/s | 100% [Fe_2_S_2_]^2+^ | [68, 81, 147, 254, 255] |
| Isa1 | 0.5 [Fe_4_S_4_] (S)_4_ and  0.5 [Fe_2_S_2_] (S)_4_ | E^0^ ≈ -700 mV (uncertain) | Site 1: 33% S = 0; δ = 0.46 mm/s; ΔE_Q_ = 1.25 mm/s  Site 2: 33% S = 0; δ = 0.44 mm/s; ΔE_Q_ = 1.04 mm/s  Site 3: 33% S = 0; δ = 0.26 mm/s; ΔE_Q_ = 0.55 mm/s | 100% [Fe_4_S_4_]^2+^  100% [Fe_2_S_2_]^2+^ | [256-261] |
| Isa2 | 0.5 [Fe_4_S_4_] and  0.5 [Fe_2_S_2_] | E^0^ ≈ -700 mV (uncertain; same as Isa1) | Site 1: 33% S = 0; δ = 0.46 mm/s; ΔE_Q_ = 1.25 mm/s  Site 2: 33% S = 0; δ = 0.44 mm/s; ΔE_Q_ = 1.04 mm/s  Site 3: 33% S = 0; δ = 0.26 mm/s; ΔE_Q_ = 0.55 mm/s | 100% [Fe_4_S_4_]^2+^  100% [Fe_2_S_2_]^2+^ | [256-261] |
| Isu1 | 1 [Fe_2_S_2_] | E^0^ ≈ -700 mV (uncertain) | Site 1: 50% S = 0; δ = 0.26 mm/s; ΔE_Q_ = 0.64 mm/s  Site 2: 50% S = 0; δ = 0.32 mm/s; ΔE_Q_ = 0.91 mm/s | 100% [Fe_2_S_2_]^2+^ | [67, 262] |
| Isu2 | 1 [Fe_2_S_2_] | E^0^ ≈ -700 mV (uncertain; same as Isu1) | Site 1: 50% S = 0; δ = 0.26 mm/s; ΔE_Q_ = 0.64 mm/s  Site 2: 50% S = 0; δ = 0.32 mm/s; ΔE_Q_ = 0.91 mm/s  (same as Isu1) | 100% [Fe_2_S_2_]^2+^ | [67, 262] |
| Lip5 | 2 [Fe_4_S_4_] | E^0^ = -505 mV (1st cluster)  E^0^ = -430 mV (2^nd^ cluster) | Site 1: 50% δ = 0.45mm/s ΔE_Q_ = 0.98 mm/s; η = 0.5  Site 2: 50% δ = 0.46 mm/s; ΔE_Q_ = 1.30 mm/s; η = 0.6 | 100% [Fe_4_S_4_]^2+^ | [76, 263] |
| Lys4 | 1 [Fe_4_S_4_] | E^0^ = -450 mV (matrix)  (Same as aconitase) | Same as aconitase  Site 1: 87.5% S = 0; δ = 0.45 mm/s; ΔE_Q_ = 1.30 mm/s.  Site 2; 6.25% S = 0; δ = 0.84 mm/s; ΔE_Q_ = 1.26 mm/s  Site 3: 6.25% ; S = 0; δ = 0.89 mm/s; ΔE_Q_ = 1.83 mm/s | 100% [Fe_4_S_4_]^2+^ | Same as aconitase |
| Mss51 | 2 heme b at 50% occupancy | E^0^ = +160 mV (same as Cox15) | Site 1: 100%; δ = 0.93 mm/s; ΔE_Q_ = 1.85 mm/s  (uncertain) | 100% [Fe^II^] | [59] |
| Nfu1 | 1 [Fe_4_S_4_] | E^0^ ≈ -700 mV (uncertain) | Site 1: 50% S = 0; δ = 0.45 mm/s ΔE_Q_ = 1.13 mm/s;  Site 2: 50% S = 0 δ = 0.47 mm/s ΔE_Q_ = 1.09 mm/s; | 100% [Fe_4_S_4_]^2+^ | [251, 264 – 266] |
| Rip1 | 1 [Fe_2_S_2_] (His)_2_ (Cys)_2_ | E^0^ = +285 mV | Site 1: 50% S = ½; δ = 0.31 mm/s ΔE_Q_ = 0.63 mm/s; η = 0; A_xyz_ = -55, -50, and -43 MHz. (values should be negative)  Site 2: 50% δ = 0.74 mm/s ΔE_Q_ = 3.05 mm/s; η = 0;  A_xyz_ = 11, 14, and 33 MHz.  Extra EPR = g = 2.02, 1.90, 1.80.  S = 0 [Fe_2_S_2_]^2+^  Site 1: δ = 0.24 mm/s ΔE_Q_ = 0.52 mm/s  Site 2: δ = 0.32 mm/s ΔE_Q_ = 0.91 mm/s | 100% [Fe_2_S_2_]^1+^ | [67, 267, 268] |
| Sdh2 | 1 [Fe_4_S_4_], 1[Fe_3_S_4_], 1[Fe_2_S_2_] | E^0^ [Fe_4_S_4_]^2+/1+^ = -220 mV  E^0^ [Fe_3_S_4_]^1+/0^ = +62 mV  E^0^ [Fe_2_S_2_]^2+/1+^ = +5 mV | S = 0 [Fe_4_S_4_]^2+^ per dimer  Site 1: 5% S = 0; δ ≈ 0.45 mm/s; ΔE_Q_ ≈ 1.1 mm/s  Site 2: 19,5% S = ½ ; δ = 0.52 mm/s; ΔE_Q_ = 1.0 mm/s; η = 0.6;  A_xyz_ = -37 MHz; -25 MHz; -25 MHz  Site 3: 19.5%; δ = 0.58 mm/s; ΔE_Q_ = 1.6 mm/s; η = 0.5  A_xyz_ = +22 MHz; +17 MHz; +10 MHz  Site 4: 22% S = 2; δ = 0.46 mm/s; ΔEQ = 1.47 mm/s. eta = 0.4; Axyz = -20.5; -20.5, -16.4 MHz  Site 5 11%; S = 2; δ = 0.32 mm/s; ΔE_Q_ = -0.52 mm/s; eta = -2; Axyz = +13.7, +15.8, +15.6  Site 6: 22% S = 0; δ ≈ 0.28 mm/s; ΔE_Q_ ≈ 0.64 mm/s | 12%[Fe_4_S_4_]^2+^  88%[Fe_4_S_4_]^1+^  100%[Fe_3_S_4_]^0^  100%[Fe_2_S_2_]^1+^ | [269 – 271] |
| Sdh3 | 1 LS heme b | E^0^ = +48 mV | Site 1: 100% S = 0; δ = 0.45 mm/s; ΔE_Q_ = 1.17 mm/s | 100%[Fe^II^] | [27, 272 – 275] |
| Sdh4 | 1 LS heme b | E^0^ = +48 mV | Site 1: 100% S = 0; δ = 0.45 mm/s; ΔE_Q_ = 1.17 mm/s | 100%[Fe^II^] | [272 – 275] |
| Thi4 (not included) | Fe^II^ (His)(Asp)(Glu) | E0 ≈ +20 mV (ignoring hysteresis)  Extra:  E^0^ for Fe^III^ 🡪 Fe^II^ = -127 mV  E^0^ Fe^II^ 🡪 Fe^III^ = +171 mV | Same as TauD  Site 1: 50% S = 2; δ = 1.27 mm/s; ΔE_Q_ = 3.06 mm/s  Site 2: 50%; S = 2; δ = 1.16 mm/s; ΔEQ = 2.76 mm/s  (in present of 2OG) | 100%[Fe^II^] | [78, 79] |
| Thi5 (not included) | Fe^II^ (His)(Asp)(Glu) | E0 ≈ +20 mV (ignoring hysteresis)  Extra:  E^0^ for Fe^III^ 🡪 Fe^II^ = -127 mV  E^0^ Fe^II^ 🡪 Fe^III^ = +171 mV | Same as TauD  Site 1: 50% S = 2; δ = 1.27 mm/s; ΔE_Q_ = 3.06 mm/s  Site 2: 50%; S = 2; δ = 1.16 mm/s; ΔEQ = 2.76 mm/s  (in present of 2OG) | 100%[Fe^II^] | [78, 79] |
| Yah1 | 1 [Fe_2_S_2_] | E^0^ = -353 mV | Site 1: 78% S = 0; δ ≈ 0.28 mm/s; ΔE_Q_ ≈ 0.64 mm/s  Site 2: 11% S = ½ δ = 0.32 mm/s; ΔE_Q_ = 0.8 mm/s; η = -0.4; A_xyz_ = -56, -50, -42 (MHz)  Site 3: 11% S = ½ ; δ = 0.65 mm/s; ΔE_Q_ = 2.9 mm/s; η = 0.4; A_xyz_ = 24, 13, 35 (MHz) | 78%[Fe_2_S_2_]^2+^  22%[Fe_2_S_2_]^1+^ | [70, 157, 276] |
| Yfh1 | 1 Fe^II^_O/N_ | E^0^ = -110 mV (uncertain) | Site 1: S = 2; δ ≈ 1.3 mm/s; ΔE_Q_ ≈ 3.0 mm/s | 100% [Fe^II^] | [277 – 280] |
|  |  |  |  |  |  |
| **Nucleus** |  |  |  |  |  |
| **Protein** | **Iron center(s) per monomer** | **E’_Nuc_ = -290 mV** | **Mössbauer properties** |  | **Reference** |
| Chl1 | 1[Fe_4_S_4_](S)_4_ | E^0^ = -700 mV | Site 1; 100%; S = 0; δ = 0.45 mm/s; ΔE_Q_ = 1.15 mm/s | 100%[Fe_4_S_4_]^2+^ | [91, 281] |
| Dna2 | 1[Fe_4_S_4_](S)_4_ | E^0^ = -700 mV | Site 1; 100%; S = 0; δ = 0.45 mm/s; ΔE_Q_ = 1.15 mm/s | 100%[Fe_4_S_4_]^2+^ | [92, 282, 283] |
| Hap1 | 1 heme b with cys coordination | E^0^ = -170 mV (HS) and  E^0^ = -270 mV (LS). | Site 1; 50%; S = 5/2 δ = 0.44 mm/s; ΔE_Q_ = 0.78 mm/s; η = 0.59  Axyz = -723, -361, -163 (kG) gxyz = 8, 4, 1.8  Site 2; 50%; S = 0; δ = 0.83 mm/s; ΔE_Q_ = 2.46 mm/s | 100%[Fe^II^] HS  100%[Fe^II^] LS | [167, 284, 285] |
| Hap4 (not included) |  |  | Similar to Hap1 |  | [100] |
| Ntg2 | 1[Fe_4_S_4_](S)_4_ | E^0^ = -700 mV | Site 1; 100%; S = 0; δ = 0.45 mm/s; ΔE_Q_ = 1.15 mm/s | 100%[Fe_4_S_4_]^2+^ | [172, 286, 287] |
| Pol1 | 1[Fe_4_S_4_](S)_4_ | E^0^ = -700 mV | Site 1; 100%; S = 0; δ = 0.45 mm/s; ΔE_Q_ = 1.15 mm/s | 100%[Fe_4_S_4_]^2+^ | [288, 289] |
| Pol2 | 1[Fe_4_S_4_](S)_4_ | E^0^ = -700 mV | Site 1; 100%; S = 0; δ = 0.45 mm/s; ΔE_Q_ = 1.15 mm/s | 100%[Fe_4_S_4_]^2+^ | [94] |
| Pol3 | 1[Fe_4_S_4_](S)_4_ | E^0^ = -700 mV | Site 1; 100%; S = 0; δ = 0.45 mm/s; ΔE_Q_ = 1.15 mm/s | 100%[Fe_4_S_4_]^2+^ | [95] |
| Pri2 | 1[Fe_4_S_4_](S)_4_ | E^0^ = -700 mV | Site 1; 100%; S = 0; δ = 0.45 mm/s; ΔE_Q_ = 1.15 mm/s | 100%[Fe_4_S_4_]^2+^ | [96] |
| Rad3 | 1[Fe_4_S_4_](S)_4_ | E^0^ = -700 mV | Site 1; 100%; S = 0; δ = 0.45 mm/s; ΔE_Q_ = 1.15 mm/s | 100%[Fe_4_S_4_]^2+^ | [97] |
| Rev3 | 1[Fe_4_S_4_](S)_4_ | E^0^ = -700 mV | Site 1; 100%; S = 0; δ = 0.45 mm/s; ΔE_Q_ = 1.15 mm/s | 100%[Fe_4_S_4_]^2+^ | [288] |
| Tpa1 | Fe^II^ (His)(Asp)(Glu) | E0 ≈ +20 mV (ignoring hysteresis)  Extra:  E^0^ for Fe^III^ 🡪 Fe^II^ = -127 mV  E^0^ Fe^II^ 🡪 Fe^III^ = +171 mV | Same as TauD  Site 1: 50% S = 2; δ = 1.27 mm/s; ΔE_Q_ = 3.06 mm/s  Site 2: 50%; S = 2; δ = 1.16 mm/s; ΔEQ = 2.76 mm/s  (in present of 2OG) | 100%[Fe^II^] | [99, 161, 290] |
| Yap5 | 1.5 [Fe_2_S_2_]; 0.5 [Fe_4_S_4_] | E^0^ = -400 mV (uncertain) | Site 1 (N-CRD): 60% S = 0; δ = 0.31 mm/s; ΔE_Q_ = 0.50 mm/s  Site 2 (C-CRD); 40% δ = 0.45 mm/s ΔE_Q_ = 0.96 mm/s | 100%[Fe_X_S_X_]^2+^ | [102, 291] |
|  |  |  |  |  |  |
| **Endoplasmic Reticulum** |  |  |  |  |  |
| **Protein** | **Iron center(s) per monomer** | **E’_ER_ = -220 mV** | **Mössbauer properties** |  | **Reference** |
| Cyb5 | LS heme (His)_2_ | E0 = -160 mV | Site 1: 10% S = ½; δ = 0.20 mm/s; ΔE_Q_ = 1.77 mm/s; η = -2; Ax,y,z = -40.3, +19.3, +53.5 MHz; gxyx = 1.44, 2.27, 3.00. (same as Ccp1)  Site 2: 90%; S = 0; δ = 0.45 mm/s; ΔE_Q_ = 1.15 mm/s | 10% [Fe^III^]  90% [Fe^II^] | [106, 292, 293] |
| Cyp51 | P450 heme (cys)_1_ | E^0^ = -170 mV (high spin) E^0^ = -270 mV (low spin). | Site 1; 6%; S = 5/2; δ = 0.44 mm/s; ΔE_Q_ = 0.78 mm/s; η = 0.59;  A_xyz_/g_n_β_n_ = -723, -361, -163 kG  g_xyz_ = 8, 4, 1.8  Site 2: 44% S = ½ δ = 0.38 mm/s; ΔE_Q_ = 2.85 mm/s; η = 0.59;  A_xyz_/g_n_β_n_ = -450, -102, -191 kG  g_xyz_ = 2.45, 2.26, 1.91  Site 3: 50% S = 2; δ = 0.83 mm/s; ΔE_Q_ = 2.46 mm/s | High spin  6%[ox]  50%[red]  Low spin  44%[ox] | [284, 294 – 296] |
| Dap1 | Heme b (tyrosine)_1_ | E^0^ < -500 mV (uncertain; like catalase) | Site 1: 100% S = 5/2; δ = 0.40 mm/s; ΔE_Q_ = 1.00 mm/s; A_xyz_/(g_n_β_n_) = 21, 19, 18 T  D = 10 cm^-1^, g = 2.0, E/D = 0.01, Γ = 0.3 mm/s.  (uncertain; like catalase) | 100%[Fe^III^] | [107, 152, 297 – 301] |
| Erg3 | [Fe-O-Fe] | E0 = -110 mV. | Site 1: 50%; S = 2; δ = 1.30 mm/s; ΔE_Q_ = 3.04 mm/s  Site 2: 50%; S = 2; δ = 1.30 mm/s; ΔE_Q_ = 3.36 mm/s  (like sterol delta 5,6-desaturase)  Extra:  S = 0 FeIII-O-FeIII  Site 1: d = 0.53 mm/s; DEQ = 1.54  Site 2: d = 0.50 mm/s DEQ = 0.74  Mo¨ssbauer of resting 9D showed that both oxo- and hydroxo-bridged diiron centers were present  (oxo, ¢EQ1 ) DEQ = 1.53 mm/s, d = 0.54 mm/s, 72%; hydroxo, ΔEQ2 ) DEQ = 0.72 mm/s, d = 0.49 mm/s, 21%) | 100%[FeII] | [109, 302, 303] |
| Erg5 | Heme (P450) | E^0^ = -170 mV (high spin) E^0^ = -270 mV (low spin). | Site 1; 6%; S = 5/2; δ = 0.44 mm/s; ΔE_Q_ = 0.78 mm/s; η = 0.59;  A_xyz_/g_n_β_n_ = -723, -361, -163 kG  g_xyz_ = 8, 4, 1.8  Site 2: 44% S = ½ δ = 0.38 mm/s; ΔE_Q_ = 2.85 mm/s; η = 0.59;  A_xyz_/g_n_β_n_ = -450, -102, -191 kG  g_xyz_ = 2.45, 2.26, 1.91  Site 3: 50% S = 2; δ = 0.83 mm/s; ΔE_Q_ = 2.46 mm/s | High spin  6%[ox]  50%[red]  Low spin  44%[ox] | [304, 305] |
| Erg25 | [Fe-O-Fe] | E^0^ = -110 mV.  (same as Erg3) | Site 1: 50%; S = 2; δ = 1.30 mm/s; ΔE_Q_ = 3.04 mm/s  Site 2: 50%; S = 2; δ = 1.30 mm/s; ΔE_Q_ = 3.36 mm/s  (like sterol delta 5,6-desaturase and Erg3) | 100%[FeII] | [108, 212, 302, 303, 306, 307] |
| Fre8 | 2 LS Heme b (His)_2_  (same as Fre1) | E^0^ = -250 mV. (same as Fre1) | Site 1: 24% S = 0; δ = 0.4 mm/s; ΔE_Q_ = 1.1 mm/s.  Site 2: 76% S = ½; δ = 0.20 mm/s; ΔE_Q_ = 1.77 mm/s | 24% [Fe^II^]  76% [Fe^III^] | [54, 189 – 192] |
| Grx6 | [Fe_2_S_2_](S)_2_(N)_2_ | E^0^’[Fe_2_S_2_]^2+/1+^ = -400 mV (same as Grx3/4) | Site 1: 50%; S = 0; δ = 0.30 mm/s; ΔE_Q_ = 0.50 mm/s; Γ = 0.32 mm/s  Site 2: 50%; S = 0; δ = 0.30 mm/s; ΔE_Q_ = 0.82 mm/s; Γ = 0.32 mm/s | 100%[Fe_2_S_2_]^2+^ | [21, 74, 163 – 165, 308 – 311] |
| Hmx1 | Heme b | E^0^ = -65 mV | Site 1: 100%; S = 2; δ = 0.92 mm/s; ΔE_Q_ = -2.24 mm/s; η = 0.9; Axyz/gnBn = -6.4, -20, -10 T; D = -10 cm^-1^; E/D = 1/3. | 100% [Fe^II^] | [117, 304 – 307] |
| Mpo1 | 1 Fe^II^_O/N_ | Same as Bna1 (TauD) | Site 1: 100% S = 2; δ = 1.28 mm/s; ΔE_Q_ = 2.22 mm/s. | 100% [Fe^II^] |  |
| Ole1 | [Fe-O-Fe] | E^0^ = -110 mV. | Site 1: 50%; S = 2; δ = 1.30 mm/s; ΔE_Q_ = 3.04 mm/s  Site 2: 50%; S = 2; δ = 1.30 mm/s; ΔE_Q_ = 3.36 mm/s  (like sterol delta 5,6-desaturase) | 100%[Fe^II^] | [212, 303, 312] |
| Scs7 | 1 [Fe-O-Fe]; 1 heme b | Same as Erg3 E^0^ = -110  Same as Cyb5 E^0^ = -160 mV | Site 1: 33%; S = 2; δ = 1.30 mm/s; ΔE_Q_ = 3.04 mm/s  Site 2: 33%; S = 2; δ = 1.30 mm/s; ΔE_Q_ = 3.36 mm/s  Site 3: 33% S = 0 δ = 0.45 mm/s; ΔE_Q_ = 1.17 mm/s. | 100%[Fe^II^-O-Fe^II^]  100%[Fe^II^ heme] | [303, 313] |
| Sfh5 | Heme b (tyrosine)_1_ | E^0^ < -500 mV  (like catalase) | Site 1: 59%; S = 5/2; δ = 0.40 mm/s; ΔE_Q_ = 0.55 mm/s; η = 0.83; D = 2.5 cm-1; E/D = 0.072; Axyz/gnBn = -57, -171, -216 kG; Γ = 0.48  Site 2: 41%; S = 5/2; δ = 0.37 mm/s; ΔE_Q_ = 0.91 mm/s; η = 0; D = 2.0 cm-1; Axyz/gnBn = -121, -58, -14 kG; Γ = 0.45 | 100%[Fe^III^] | [116] |
| Sur2 | 1 [Fe-O-Fe]; | E^0^ = -110 mV. | Site 1: 50%; S = 2; δ = 1.30 mm/s; ΔE_Q_ = 3.04 mm/s  Site 2: 50%; S = 2; δ = 1.30 mm/s; ΔE_Q_ = 3.36 mm/s  (like sterol delta 5,6-desaturase) | 100%[Fe^II^] | [303, 313] |
| Yno1 | 2 heme b His coordination Low spin | E^0^ = -250 mV | Site 1: 75% S = ½; δ = 0.20 mm/s; ΔE_Q_ = 1.77 mm/s η = -2; Ax,y,z = -40.3, +19.3, +53.5 MHz; gxyx = 1.44, 2.27, 3.00. (same as Ccp1)  Site 2: 25% S = 0; δ = 0.4 mm/s; ΔE_Q_ = 1.1 mm/s. | 76%[Fe^III^]  24%[Fe^II^] | [192, 314, 315] |
| **Vacuoles** |  |  |  |  |  |
| **Protein** | **Iron center(s) per monomer** | **E’_vac_ = -180** | **Mössbauer properties** |  | **Reference** |
| Fre6 | 2 LS S = 0 Fe^II^ hemes with 2 His ligands | E^0^ = -250 mV | Site 1: 94% S = ½; δ = 0.20 mm/s; ΔE_Q_ = 1.77 mm/s; η = -2; Ax,y,z = -40.3, +19.3, +53.5 MHz; gxyx = 1.44, 2.27, 3.00. (same as Ccp1 and Fre1)  Site 2: 6%; S = 0; δ = 0.4 mm/s; ΔE_Q_ = 1.1 mm/s. | 94%[Fe^III^]  6%[Fe^II^] | [54, 189- 192] |

**Table S8. Local iron concentrations and fractions of mitochondrial iron due to individual proteins under fermenting (Fsa) and respiring (Rsa) conditions.**

|  | **Mitochondria** | **Local Iron concentration** | **Fraction of mito iron** | **Local Iron concentration** | **Fraction of mito iron** |
| --- | --- | --- | --- | --- | --- |
| **Number** | **Protein** | **Fsa** |  | **Rsa** |  |
| 1 | Aco1 | 326.4 | 0.318 | 240.44 | 0.320 |
| 2 | Aco2 | 79.2 | 0.077 | 6.50 | 0.009 |
| 3 | Aim32 | 1.64 | 0.002 | 0.40 | 0.001 |
| 4 | Bio2 | 19.62 | 0.019 | 2.34 | 0.003 |
| 5 | Bol1 | 6.09 | 0.006 | 5.52 | 0.007 |
| 6 | Bol3 | 2.34 | 0.002 | 0.60 | 0.001 |
| 7 | Ccp1 | 20.32 | 0.020 | 14.38 | 0.019 |
| 8 | Cob1 | 34.8 | 0.034 | 45.8 | 0.061 |
| 9 | Coq7 | 4.36 | 0.004 | 0.29 | 0.000 |
| 10 | Cox1 | 15.86 | 0.015 | 22.8 | 0.030 |
| 11 | Cox10 | 0.88 | 0.001 | 0.88 | 0.001 |
| 12 | Cox15 | 9.72 | 0.009 | 2.44 | 0.003 |
| 13 | Cyb2 | 7.40 | 0.007 | 19.21 | 0.026 |
| 14 | Cyc1 | 37.97 | 0.037 | 61.04 | 0.081 |
| 15 | Cyc7 | 4.06 | 0.004 | 1.50 | 0.002 |
| 16 | Cyt1 | 17.4 | 0.017 | 22.9 | 0.030 |
| 17 | Exo5 | 8.26 | 0.008 | 8.26 | 0.011 |
| 18 | Fre5 | 2.65 | 0.003 | 2.65 | 0.004 |
| 19 | Grx5 | 59.60 | 0.058 | 35.17 | 0.047 |
| 20 | Hem15 | 10.36 | 0.010 | 4.47 | 0.006 |
| 21 | Ilv3 | 107.96 | 0.105 | 28.02 | 0.037 |
| 22 | Isa1 | 5.55 | 0.005 | 5.55 | 0.007 |
| 23 | Isa2 | 5.19 | 0.005 | 0.39 | 0.001 |
| 24 | Isu1 | 10.50 | 0.010 | 1.33 | 0.002 |
| 25 | Isu2 | 5.58 | 0.005 | 0.04 | 0.000 |
| 26 | Lip5 | 22.96 | 0.022 | 2.77 | 0.004 |
| 27 | Lys4 | 52.72 | 0.051 | 4.84 | 0.006 |
| 28 | Mss51 | 5.21 | 0.005 | 1.32 | 0.002 |
| 29 | Nfu1 | 21.80 | 0.021 | 9.88 | 0.013 |
| 30 | Rip1 | 34.8 | 0.034 | 45.8 | 0.061 |
| 31 | Sdh2 | 67.77 | 0.066 | 134.1 | 0.178 |
| 32 | Sdh3 | 3.76 | 0.004 | 7.45 | 0.010 |
| 33 | Sdh4 | 3.76 | 0.004 | 7.45 | 0.010 |
| 34 | Thia4 | 3.13 | 0.003 | 3.13 | 0.004 |
| 35 | Thia5 | 1.26 | 0.001 | 1.26 | 0.002 |
| 36 | Yah1 | 5.26 | 0.005 | 0.44 | 0.001 |
| 37 | Yfh1 | 1.40 | 0.001 | 0.29 | 0.000 |
| total |  | 1027.54 | 1.000 | 751.65 | 1.000 |


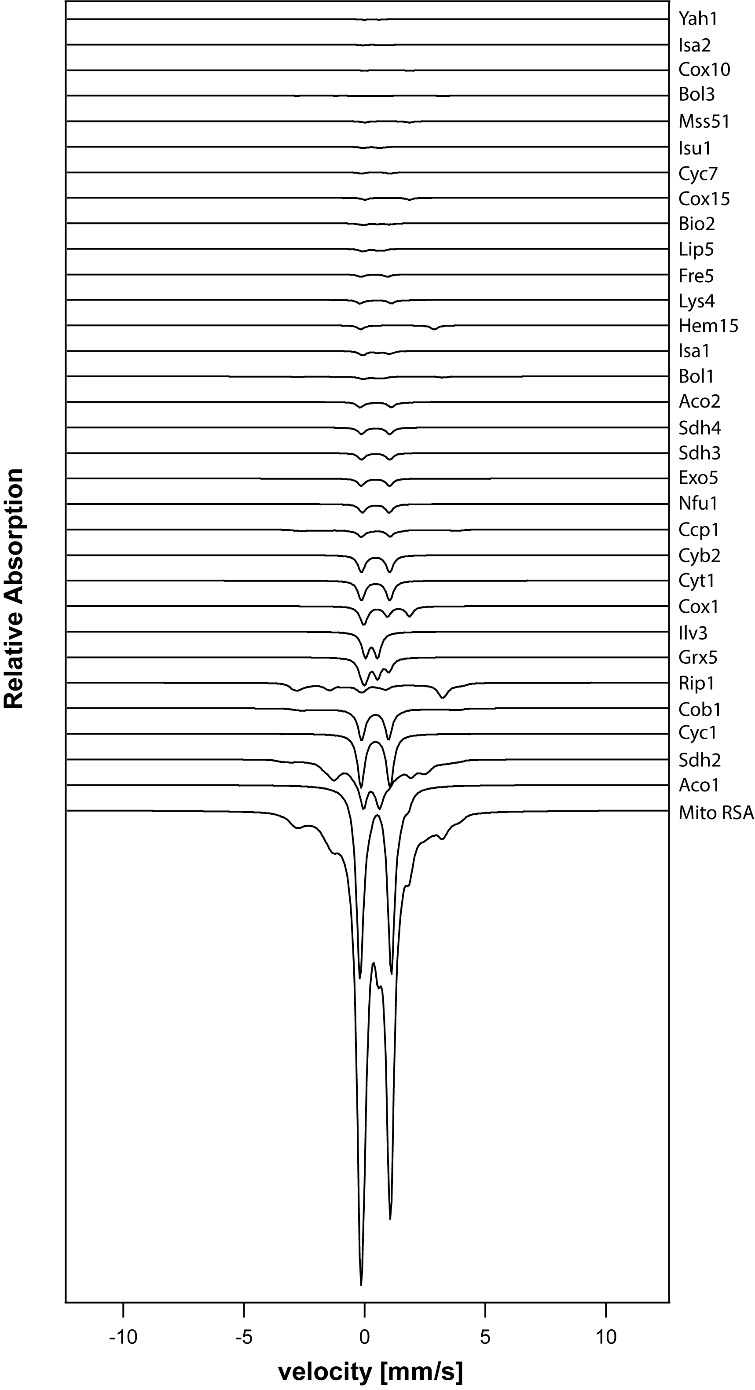
**Figure S2: Simulated Mössbauer spectrum of mitochondria isolated from respiring cells, obtained by summing the iron contributions from individual mitochondrial proteins.** Spectral contributions from Thi4, Thi5 and Aim32 were not included. Collectively these proteins contribute 0.7% of the total iron in the organelle.


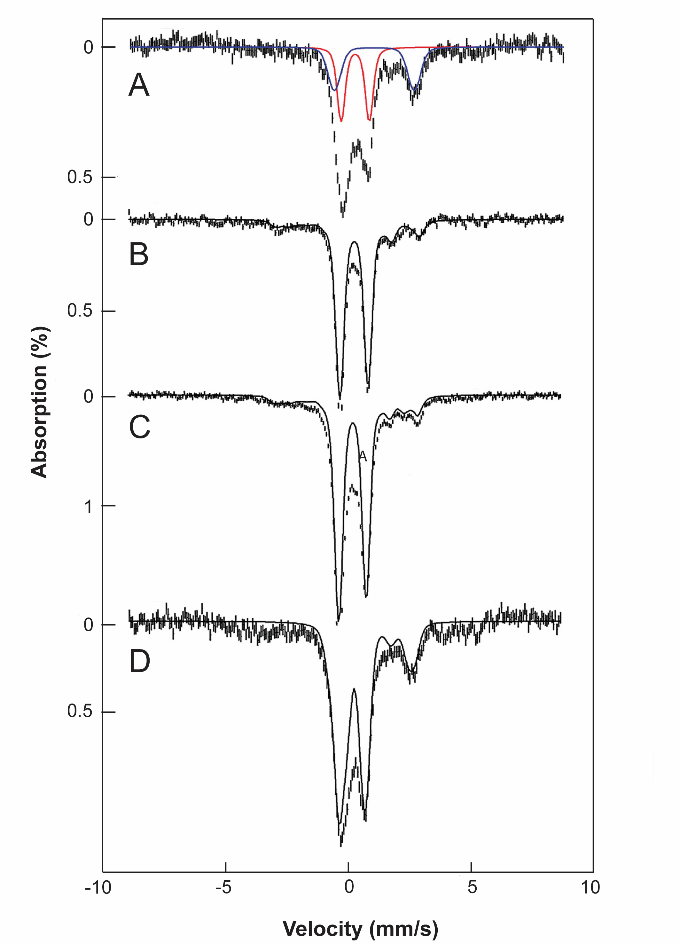
**Figure S3: Experimental Mössbauer spectra of mitochondria.** A: EGTA-washed fermenting mitochondria. Solid lines simulate the contribution of NHHS FeII blue and central doublet (red); B: mitochondria isolated from respiring cells. The solid black line is the simulation for central doublet, HS Fe^II^ hemes, S = 1/2 [Fe_2_S_2_]^+^ and NHHS Fe^II^; C: mitochondria from respirofermenting cells. The solid black line is the simulation; D: mitochondria from fermenting cells. The solid black line is the simulation. All the spectra were collected at ~5 K with 0.05 T field applied parallel to the gamma radiation. Images were extracted with permission from the publishers [128, 134]. Parameters for simulations are given in the original papers.

**Figure S4: Experimental Mössbauer spectra of isolated vacuoles (A), fermenting whole yeast cells (B), and respiring yeast cells (C).** Images were extracted with permission from the publishers [5, 11, 148]. The red line in A is a simulation of HS S = 5/2 Fe^III^ [5]. That in B was a composite with percentages 73% HS Fe^III^, 5% CD, 10% NHHS Fe^II^, and 12% Fe^III^ nanoparticles [148]. The red line in C was also a composite, with 52% HS Fe^III^, 25% CD, 8% NHHS Fe^II^, 6% HS Fe^II^ hemes, and 9% [Fe_2_S_2_]^2+^ clusters [11].


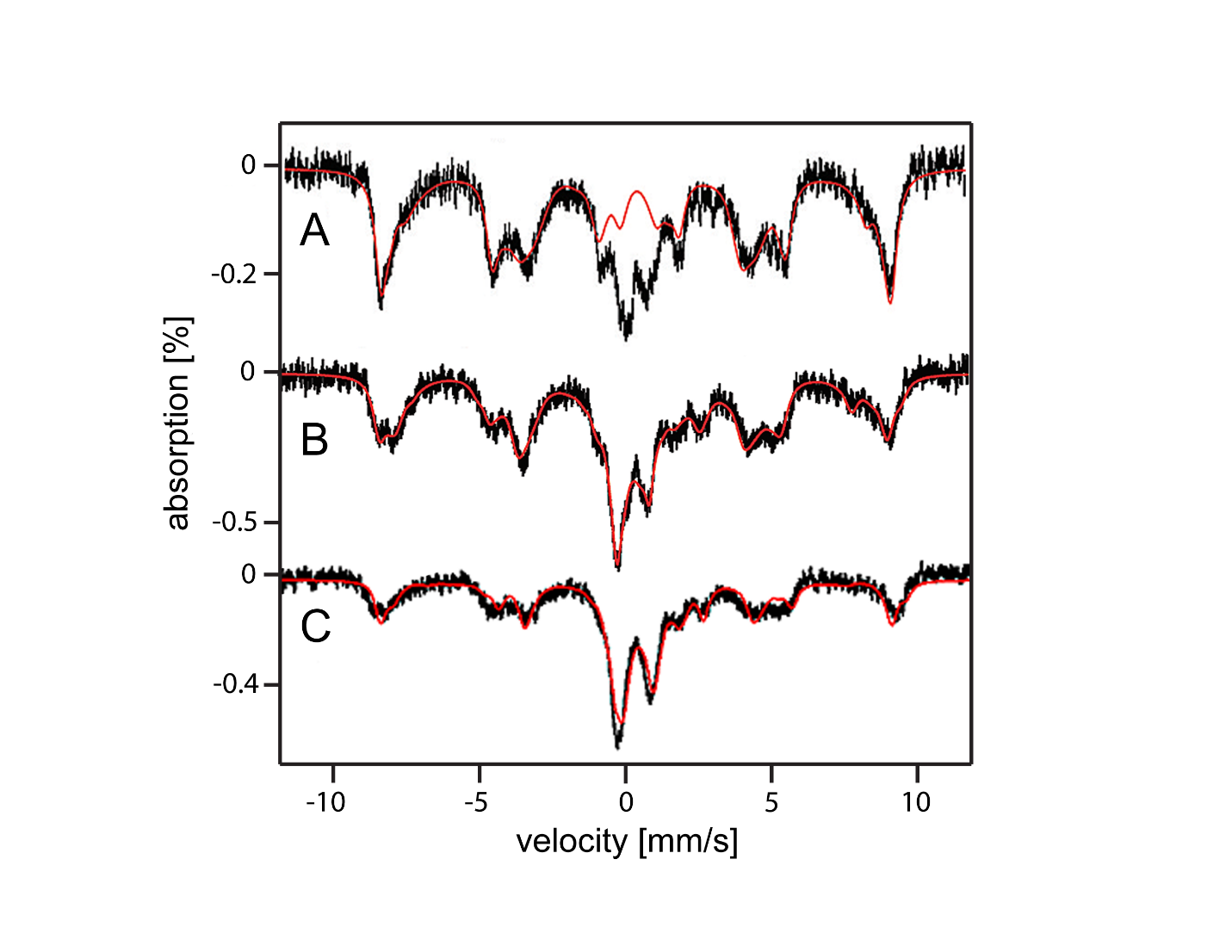


**Figure S5: Confocal microscopic images of isolated nuclei.** A, Contrast microscopy image of the isolated nuclei showing the presence of intact nuclei. B, Fluorescence microscopy image of an isolated nucleus stained left-to-right with DAPI, Dil2C, and GFP. The merge is shown on the far right.


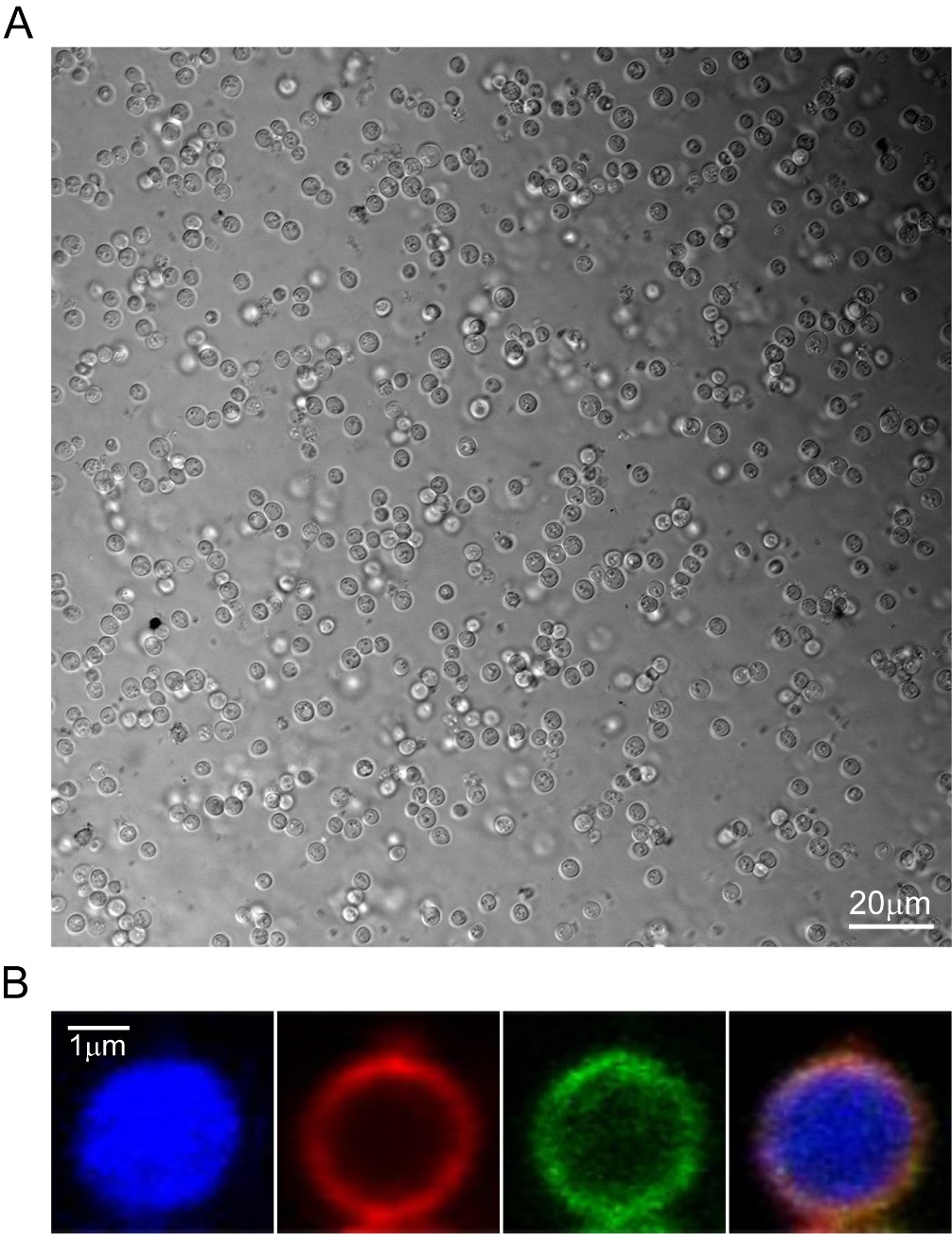


**Figure S6: Individual Mössbauer spectra of isolated nuclei.** Spectra (i) and (ii) are from nuclei isolated from cells grown in 40 µM ^57^Fe. Spectra (iii) and (iv) are from nuclei isolated from cells grown in 1 µM ^57^Fe. Arrows indicate non-heme high-spin Fe^III^ species. Each spectrum was collected for 250-350 hours. The parameters for the simulations are provided in the text. The percentage contributions of CD are 50% (i), 65 (ii), 30 (iii) and, 35 (iv). Fe^II^ contributions are 50% (i), 35% (ii), 30% (iii) and, 15% (iv). Fe^III^ contributions are 30% (iii) and 40% (iv). Nanoparticles contributions are 10% each for (iii) and (iv).


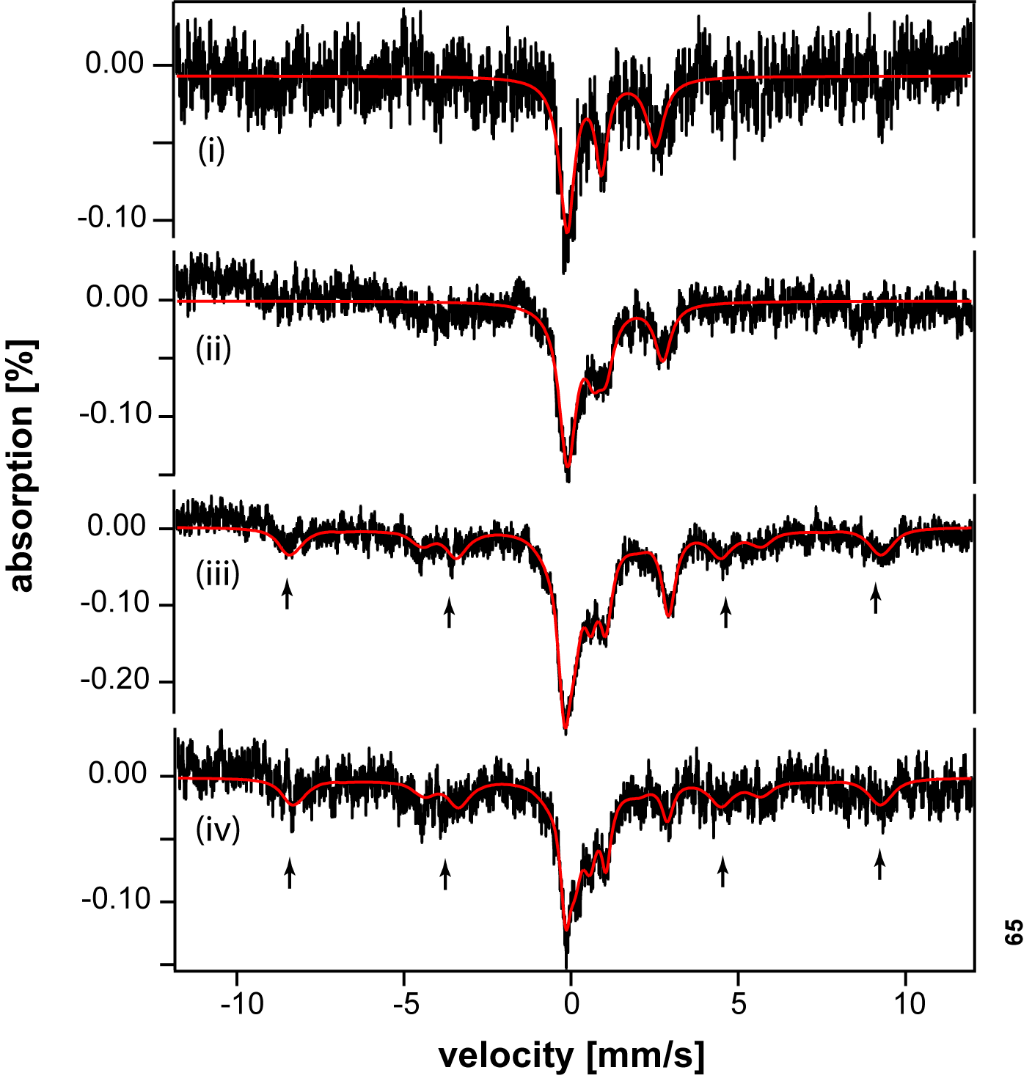

Supplement: mfac080_Supplemental_File [file mfac080_supplemental_file.docx]
